# Genome-wide imputation study identifies novel HLA locus for pulmonary fibrosis and potential role for auto-immunity in fibrotic idiopathic interstitial pneumonia

**DOI:** 10.1186/s12863-016-0377-2

**Published:** 2016-06-07

**Authors:** Tasha E. Fingerlin, Weiming Zhang, Ivana V. Yang, Hannah C. Ainsworth, Pamela H. Russell, Rachel Z. Blumhagen, Marvin I. Schwarz, Kevin K. Brown, Mark P. Steele, James E. Loyd, Gregory P. Cosgrove, David A. Lynch, Steve Groshong, Harold R. Collard, Paul J. Wolters, Williamson Z. Bradford, Karl Kossen, Scott D. Seiwert, Roland M. du Bois, Christine Kim Garcia, Megan S. Devine, Gunnar Gudmundsson, Helgi J. Isaksson, Naftali Kaminski, Yingze Zhang, Kevin F. Gibson, Lisa H. Lancaster, Toby M. Maher, Philip L. Molyneaux, Athol U. Wells, Miriam F. Moffatt, Moises Selman, Annie Pardo, Dong Soon Kim, James D. Crapo, Barry J. Make, Elizabeth A. Regan, Dinesha S. Walek, Jerry J. Daniel, Yoichiro Kamatani, Diana Zelenika, Elissa Murphy, Keith Smith, David McKean, Brent S. Pedersen, Janet Talbert, Julia Powers, Cheryl R. Markin, Kenneth B. Beckman, Mark Lathrop, Brian Freed, Carl D. Langefeld, David A. Schwartz

**Affiliations:** Center for Genes, Environment and Health, National Jewish Health, Denver, CO USA; Department of Biostatistics and Informatics, University of Colorado Denver, Aurora, CO USA; Department of Medicine, School of Medicine, University of Colorado Denver, Aurora, CO USA; Center for Public Health Genomics and Department of Biostatistical Sciences, Wake Forest School of Medicine, Winston-Salem, NC USA; Department of Biochemistry and Molecular Genetics, School of Medicine, University of Colorado Denver, Aurora, CO USA; Department of Medicine, Vanderbilt University School of Medicine, Nashville, TN USA; Department of Medicine, University of California San Francisco, San Francisco, CA USA; InterMune, Brisbane, CA USA; National Heart and Lung Institute, Imperial College, London, UK; National Institute for Health Research Biomedical Research Unit, Royal Brompton Hospital, London, UK; Department of Medicine, University of Texas Southwestern, Dallas, TX USA; Landspitali University Hospital and University of Iceland Faculty of Medicine, Reykjavik, Iceland; Department of Medicine, University of Pittsburgh, Pittsburgh, PA USA; Instituto Nacional de Enfermedades Respiratorias, Mexico City, Mexico; Universidad Nacional Autonoma de Mexico, Mexico City, Mexico; Asan Medical Center, University of Ulsan College of Medicine, Seoul, Korea; University of Minnesota Genomics Center, University of Minnesota, Minneapolis, MN USA; Fondation Jean Dausset, Centre d’Étude du Polymorphisme Humain, Paris, France; Commissariat à l’Energie Atomique, Institut Génomique, Centre National de Génotypage, Evry, France; Department of Immunology, School of Medicine, University of Colorado Denver, Aurora, CO USA

**Keywords:** Pulmonary fibrosis, HLA association, Imputation, Gene expression, RNA-Seq

## Abstract

**Background:**

Fibrotic idiopathic interstitial pneumonias (fIIP) are a group of fatal lung diseases with largely unknown etiology and without definitive treatment other than lung transplant to prolong life. There is strong evidence for the importance of both rare and common genetic risk alleles in familial and sporadic disease. We have previously used genome-wide single nucleotide polymorphism data to identify 10 risk loci for fIIP. Here we extend that work to imputed genome-wide genotypes and conduct new RNA sequencing studies of lung tissue to identify and characterize new fIIP risk loci.

**Results:**

We performed genome-wide genotype imputation association analyses in 1616 non-Hispanic white (NHW) cases and 4683 NHW controls followed by validation and replication (878 cases, 2017 controls) genotyping and targeted gene expression in lung tissue. Following meta-analysis of the discovery and replication populations, we identified a novel fIIP locus in the HLA region of chromosome 6 (rs7887 *P*_*meta*_ = 3.7 × 10^−09^). Imputation of classic HLA alleles identified two in high linkage disequilibrium that are associated with fIIP (DRB1*15:01 *P* = 1.3 × 10^−7^ and DQB1*06:02 *P* = 6.1 × 10^−8^). Targeted RNA-sequencing of the HLA locus identified 21 genes differentially expressed between fibrotic and control lung tissue (*Q* < 0.001), many of which are involved in immune and inflammatory response regulation. In addition, the putative risk alleles, DRB1*15:01 and DQB1*06:02, are associated with expression of the *DQB1* gene among fIIP cases (Q < 1 × 10^−16^).

**Conclusions:**

We have identified a genome-wide significant association between the HLA region and fIIP. Two HLA alleles are associated with fIIP and affect expression of HLA genes in lung tissue, indicating that the potential genetic risk due to HLA alleles may involve gene regulation in addition to altered protein structure. These studies reveal the importance of the HLA region for risk of fIIP and a basis for the potential etiologic role of auto-immunity in fIIP.

**Electronic supplementary material:**

The online version of this article (doi:10.1186/s12863-016-0377-2) contains supplementary material, which is available to authorized users.

## Background

The fibrotic idiopathic interstitial pneumonias (fIIPs) are a group of lung diseases characterized by progressive fibrosis of the alveolar interstitium that leads to significant morbidity and mortality; median survival of the most common form, idiopathic pulmonary fibrosis, is 2–3 years after diagnosis. Although pirfenidone [1] and nintedanib [2] have recently been shown to slow the progression of IPF, only lung transplantation has been proven to prolong survival. There is evidence for the importance of environmental factors [3–13] and both rare [14–21] and common genetic variation [22–29] in risk of fIIP. Cigarette smoking is the strongest known environmental risk factor for fIIP [30, 31], but up to one third of individuals with fIIP do not have a history of cigarette smoking.

Previously, we reported 10 genetic risk loci for fIIP based on a genome-wide association study; in aggregate, the genome-wide genotyped SNPs account for 31–33 % of the variation in risk of developing this disease [32]. The functions of the genes implicated to date in risk of fIIP indicate that host defense from inhaled insults, barrier function of the alveolar epithelium, and telomere maintenance are compromised in at least a subset of individuals with fIIP. While these studies have been revealing, the majority of risk for fIIP remains unexplained, suggesting that additional studies to identify genetic variation are warranted. In the current study, we attempt to identify additional fIIP genetic risk variants via genome-wide imputation association analyses using data from 1616 cases and 4683 out-of-study controls from our genome-wide association study (GWAS) [32]. We identify the human leukocyte antigen (HLA) region as a genetic risk locus for fIIP, demonstrating a potential role of auto-immunity in fIIP.

## Results and discussion

**Fig. 1:**
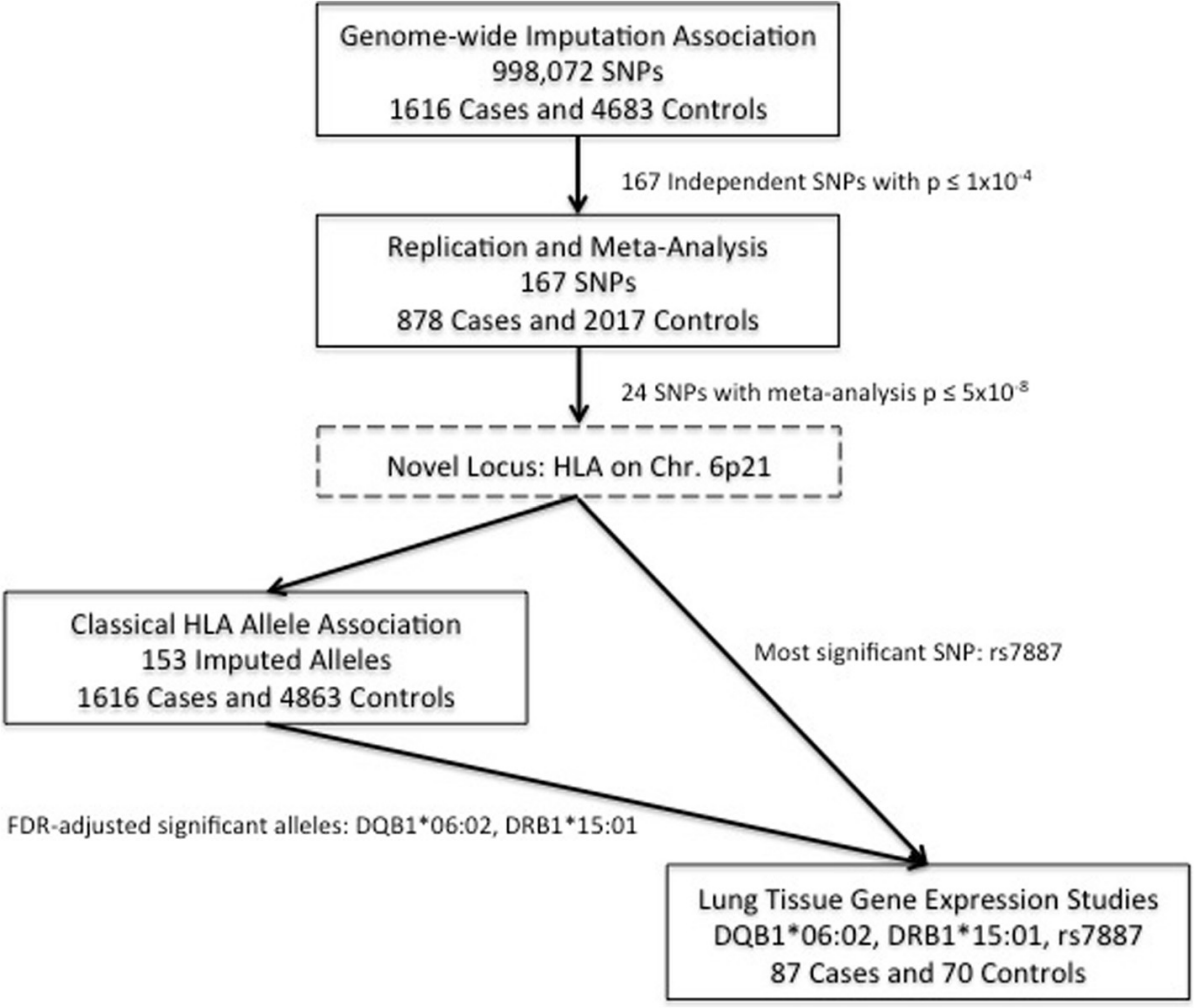
Overview of Study Design. A discovery imputation GWAS among 1616 cases and 4683 controls was followed by validation and replication genotyping in 878 cases and 2017 controls. One novel locus was identified on Chromosome 6p21. Classical HLA alleles were imputed using genotyped SNPs among the GWAS cases and controls and were tested for association with fIIP. Lung tissue gene expression was compared between a subset of GWAS cases and non-GWAS controls and across genotypes for the most significant 6p21 SNP, rs7887. Gene expression was also compared across genotypes of the most significant HLA alleles (DQB1*06:02, DRB1*15:01) within the cases with lung tissue expression data

An overview of the study design is shown in Fig. 1.

### Imputation study

We imputed genotypes using the HapMap Phase 3 integrated panel [33] using IMPUTE2 [34, 35] and genotypes at 439,828 SNPs from the Illumina 660 Quad beadchip that met strict quality control criteria [32]. We tested for association at 998,072 imputed SNPs under an additive model for each SNP with imputation information as measured by .info > 0.5 using SNPTEST v2 [36]. We adjusted the *p*-values (divided each test statistic by 1.08, then recomputed p-values) for these imputed SNPs based on the inflation in the imputation test statistics (Additional file 1: Figure S1) observed among the 439,828 genotyped SNPs compared to the imputation test statistics for those SNPs obtained from an exact mixed model that accounts for subtle stratification and cryptic relatedness among the cases and controls [37]. After this adjustment and removal of the regions known to be associated with fIIP [32], neither the Q-Q plot of p-values (Additional file 1: Figure S2) nor the genomic control inflation factor (λ = 1.04) suggested systematic departures from the null hypothesis.

We identified 205 SNPs (Additional file 1: Table S1) associated with fIIP at a genome-wide level of significance (Additional file 1: Figure S3); 204 of these SNPs are in eight fIIP risk loci (3q26, 4q22, 5p15, 6p24, 7q22, 11p15, 15q14, and 17q21) we had previously reported. The remaining SNP, rs2169877 (*P*_*imputed*_ = 2.8 × 10^−08^), is on chromosome 15q25 in an intron of the *AKAP13* gene. We selected 15 of the genome-wide significant SNPs (including rs2169877) in addition to 152 SNPs among the 337 SNPs with 5 × 10^−08^ < *P*_*imputed*_ <0.0001 for validation and replication genotyping (Additional file 1: Table S2); to avoid redundancy with known associated SNPs and among the SNPs selected for follow-up, we selected SNPs in weak LD (*r*^2^ < 0.5) with previously-known genome-wide significant SNPs [32] and chose the most strongly-associated SNP among potential replication SNPs with pair-wise *r*^2^ > 0.8. After genotype quality control and removal of putative duplicate samples between the GWAS and replication cases (see Methods), we successfully genotyped 1498 of the discovery GWAS cases for validation (we do not have access to GWAS out-of-study control DNA) and 878 cases and 2017 controls for replication at 148 of the SNPs.

**Fig. 2:**
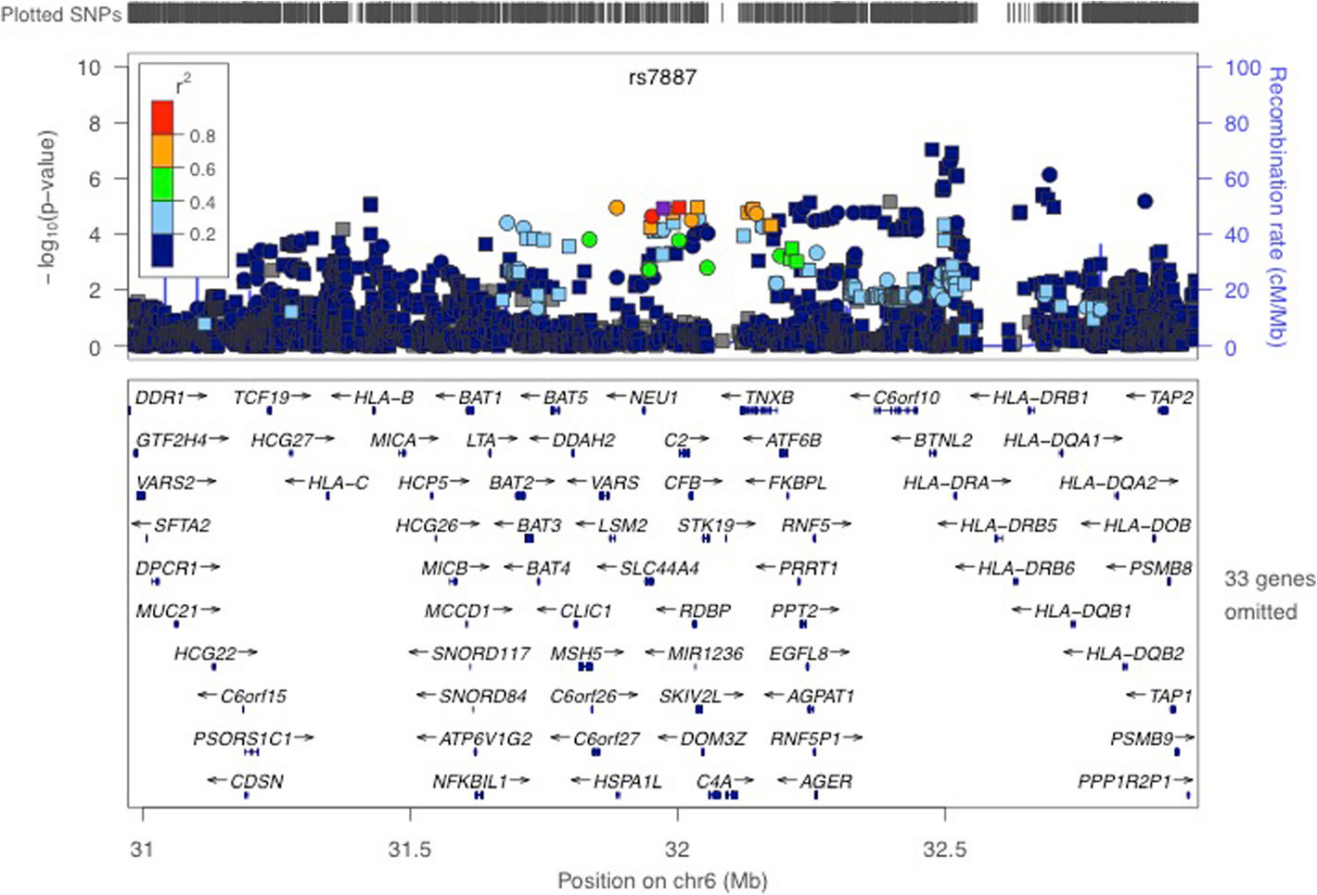
Locus-specific plot for HLA region corresponding to discovery imputation GWAS results. The –log_10_ *P* values (*y* axis) of the SNPs are shown according to their chromosomal positions (*x* axis). The estimated recombination rates (cM/Mb) from the HapMap Project (NCBI Build 36) are shown as light blue lines, and the genomic locations of genes within the regions of interest in the NCBI Build 36 human assembly are shown as arrows. SNPs shown in red, orange, green, light blue and blue have *r* ^2^ ≥ 0.8, *r* ^2^ ≥ 0.6, *r* ^2^ ≥ 0.4, *r* ^2^ ≥ 0.2 and *r* ^2^ < 0.2 with the most highly-associated SNP, respectively. SNPs with no *r* ^2^ information with most-highly associated SNP shown in grey. Circles correspond to genotyped SNPs, squares correspond to imputed SNPs. P-values correspond to discovery cohort statistical evidence only; meta-analysis p-values can be found in Table 1 and Additional file 1: Tables S1-S3

After meta-analysis of the imputation and replication phases, 24 of the 148 SNPs were associated with fIIP at a genome-wide level of significance (*P*_*Meta*_ ≤ 2.8 × 10^−08^; Additional file 1: Table S3). Among these, 6 SNPs (rs614549, rs7887, rs2844452, rs3020644, rs2280774, and rs3117116) represented 1 novel locus for fIIP in the HLA region at Chromosome 6p21; rs7887 in *EHMT2* was the most strongly-associated SNP (*P*_*Meta*_ = 3.7 × 10^−09^, Table 1; regional association shown in Fig. 2). All 6 SNPs had imputation.info scores >0.98. Rs2169877 at 15q25 was not genome-wide significant in the meta-analysis (*P*_*Meta*_ =1.6 × 10^−7^).

**Table 1:** Genome-wide Significant SNPs from Novel HLA Locus Identified in Imputation GWAS

SNP	Position	Minor allele	Nearest gene	*P* imputed^a^	Rep case *MAF* ^b^	Rep Cont. *MAF* ^c^	*P* replication^d^	*P* meta^e^	OR (95 % CI) combined^f^	*P* adjusted^g^
rs614549	31948604	C	*SLC44A4*	5.74 × 10^−05^	0.32	0.38	5.87 ×10^−05^	2.09 × 10^−08^	0.80 (0.73, 0.87)	0.26
rs7887	31972526	A	*EHMT2*	1.18 × 10^−05^	0.31	0.37	5.84 ×10^−05^	3.70 × 10^−09^	0.78 (0.71, 0.86)	NA
rs2844452	31990003	C	*C2*	1.69 × 10^−05^	0.42	0.47	0.00071	4.55 × 10^−08^	0.81 (0.74, 0.88)	0.26
rs3020644	32002605	G	*C2*	1.13 × 10^−05^	0.36	0.41	0.00062	2.68 × 10^−08^	0.80 (0.73, 0.87)	0.28
rs2280774	32036670	T	*NELFE*	1.11 × 10^−05^	0.29	0.34	0.00096	3.89 × 10^−08^	0.81 (0.74, 0.89)	0.83
rs3117116	32474995	C	*BTNL2*	9.15 × 10^−08^	0.15	0.13	0.038	2.65 × 10^−08^	1.25 (1.10, 1.41)	0.02

^a^ *P*-value from imputation analysis under additive model using discovery GWAS samples

^b^Minor allele frequency among replication cases (878)

^c^Minor allele frequency among replication controls (2017)

^d^ *P*-value from additive model among replication cases and controls

^e^ *P*-value from meta-analysis of discovery imputation and replication

^f^Odds ratio (OR) and 95 % CI for joint analysis comparing subset of GWAS cases (1498) and replication cases (878) to replication controls (2017) based on observed genotypes; GWAS cases were genotyped at same time as replication cases and controls

^g^ *P*-value from joint analysis as in (f) with adjustment for rs7887

### Identification of independent associations within each locus

Among the genome-wide significant SNPs in regions previously known to be associated with fIIP, we compared the evidence for association between the newly-identified SNPs (*N* = 24) and those we reported previously (*N* = 46). Based on both the meta-analysis p-values and joint analysis of the combined GWAS and replication cases compared to replication controls, the top SNP remained the same as originally reported except for the 3q26 and 4q22 regions (Additional file 1: Table S3). At 3q26, rs12696304 near *TERC* (*P*_*Meta*_ = 8.2 × 10^−14^) was more significant than rs6793295 (*P*_*Meta*_ = 8.3 × 10^−13^), and at 4q22 in *FAM13A*, rs2609261 (*P*_*Meta*_ = 2.4 × 10^−12^) was more significant than rs2609255 (*P*_*Meta*_ = 2.2 × 10^−11^).

To assess the evidence for multiple independent association signals within each region accounting for our original GWAS (46 genome-wide significant SNPs) and the 24 SNPs identified in this study, we tested for association with each SNP in a given region after adjusting for the most significant SNP in that region based on the meta-analysis. We compared the combined case group (GWAS discovery + replication) to the replication control group. There was no strong evidence for independent signals within each region (Additional file 1: Table S4). In particular, in the HLA region, no other SNP was associated with fIIP after adjustment for rs7887 (all *P* >0.02; Table 1).

### Classic HLA allele associations

To further interrogate the HLA region of association, we imputed classic HLA alleles for the discovery cohort using the HLA genotype imputation with attribute bagging (HIBAG) [38] pre-computed imputation probabilities for *HLA-A*, *HLA-B*, *HLA-C*, *HLA-DRB1*, *HLA-DQ* (*A1*, *B1*), and *HLA-DPB1*. The median posterior probabilities for the imputed alleles for each gene were >0.92 for all but the DRB1 gene (Additional file 1: Table S5). We tested for association under an additive (on the log-odds scale) model for each allele (Additional file 1: Table S6) and with each amino acid. Three alleles were associated with fIIP at a nominal *p*-value < 1 × 10^−5^, the level of association demonstrated by rs7887 in the GWAS discovery: DQA1*01:02 (*P* = 4.8 × 10^−6^), DQB1*06:02 (*P* = 6.1 × 10^−8^), and DRB1*15:01 (*P* = 1.3 × 10^−7^); DQB1*06:02 and DRB1*15:01 are very in high LD. While not genome-wide significant using the standard Bonferroni adjustment to achieve a study-wide α = .05 (5 × 10^−8^ corresponds to .05 divided by 1 million) based on the discovery set, both alleles are significant after false discovery rate adjustment for all the genome-wide SNP and HLA imputation statistical tests we conducted (FDR adjusted *p*-values = 0.015 and .029 for DQB1*06:02 and DRB1*15:01, respectively). After a step-wise analysis to delineate the conditionally independent HLA allele associations (see Methods), only DQB1*06:02 remained nominally significant. In a model with both the DQB1*06:02 allele and rs7887, both remained nominally significant (*P*_adj_ = 4.8 × 10^−6^, and 1.0 × 10^−3^, respectively; Table 2), indicating the potential for more than one independent signal in the region (*r*^*2*^ between rs7887 and DQB1*06:02/DRB1*15:01 = 0.22). Since the *DRB1* alleles were imputed with less confidence than the *DQB1* and *DQA1* alleles (median posterior probability 0.89, 0.97 and 0.94, respectively), and each of the three HLA alleles are nominally significant after adjustment for rs7887 (Table 2), it is possible that the *DQB1* alleles appear to be the only independent association due to less precision in allele definition for *DRB1*.

**Table 2:** HLA Allele Associations from GWAS Discovery

	Dosage frequency^a^		Best guess count^b^		Univariate results^c^		Adjusted^d^		rs7887^e^
Allele	Cases	Controls	Cases	Controls	OR (95 % CI)	*P*	OR (95 % CI) combined	*P* adjusted	*P* adjusted
DQA1*01:02	0.22	0.18	703	1725	1.28 (1.15, 1.42)	4.84 × 10^−06^	1.25 (1.12, 1.39)	5.55 × 10^−5^	1.56 × 10^−4^
DQB1*06:02	0.15	0.12	505	1131	1.40 (1.24, 1.58)	6.05 × 10^−08^	1.34 (1.18, 1.52)	4.84 × 10^−6^	1.03 × 10^−3^
DRB1*15:01	0.16	0.12	513	1151	1.37 (1.22, 1.54)	1.29 × 10^−07^	1.31 (1.16, 1.48)	9.94 × 10^−6^	9.90 × 10^−4^

^a^Estimated allele frequency based on posterior probabilities of allele assignments

^b^Estimated allele count based on posterior probabilities of allele assignments

^c^Odds ratio, 95 % Confidence Interval (CI), and *P*-value based on GWAS cases compared to GWAS controls for each allele alone

^d^Odds ratio, 95 % Confidence Interval (CI), and *P*-value based on GWAS cases compared to GWAS controls adjusted for rs7887

^e^ *P*-value for rs7887 based on GWAS cases compared to GWAS controls adjusted for HLA allele

### Lung tissue expression studies

**Fig. 3:**
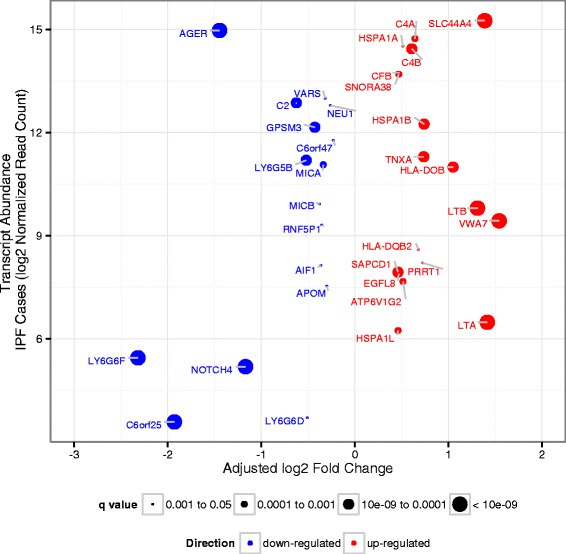
Targeted RNA-Seq Expression Differences between IPF and Control Lung. The size of the dots corresponds to q-value; larger dots have smaller q-values. The color of the dots corresponds to the direction of expression changes; genes in blue have lower expression in case lung tissue compared to control lung tissue, genes in red have higher expression

To begin to explore the biologic relevance of the association evidence, we compared lung tissue gene expression via amplicon-based targeted RNA-seq between 87 cases of idiopathic pulmonary fibrosis (IPF, the most common form of fIIP) and 70 unaffected subjects for 66 genes in the 1.54 Mb of DNA defined by HLA region association (Additional file 1: Table S7); of the 86 genes annotated in the region, 75 could be designed (*DQA1* and *DQB1* not among them) and 9 did not pass QC thresholds (see Methods). We identified 34 genes that were differentially expressed in lung tissue from patients with IPF vs. controls at an FDR of 0.05 (Additional file 1: Table S8); 21 were differentially expressed at an FDR of <1 × 10^−3^ (Fig. 3; Additional file 1: Table S8). Eight of these 21 genes have a known immune or inflammatory function (*C6orf25*, *LTA*, *LTB*, *AGER*, *HLA-DOB*, *C4A*, *C4B*, and *MICA*). In addition, two of the genes (*HSPA1L* and *HSPA1B*) encode heat-shock proteins important for mediating protein folding, a mechanism thought to be involved in the pathogenesis of IPF [39].

To determine which of the gene expression changes are more likely important for development of disease as opposed to a result of the disease process, we explored whether either rs7887 or the HLA risk alleles are expression quantitative trait loci for genes in the region. We tested whether the alleles at rs7887 were associated with expression among the cases and controls separately. The alleles at rs7887 were not associated with expression of any gene after FDR correction in either group (Additional file 1: Table S9). Among cases, both the DRB1*15:01 and DQB1*06:02 alleles were associated with expression of the *AIF1* gene (*Q* = 1.2 × 10^−5^ and *Q* = 1.2 × 10^−5^, respectively, Additional file 1: Tables S10 and S11); we were not able to infer HLA alleles for the controls in the RNA-seq study since they did not have genome-wide genotyping available. Several HLA genes, including the *DQB1* gene, were not represented in our RNA-seq expression data, but were represented in existing Affymetrix gene array expression data for 66 IPF cases. The DRB1*15:01 and DQB1*06:02 alleles were associated with increased expression of the *DRB5* (*Q* = 6.7 × 10^−22^ and *Q* = 1.06 × 10^−21^, respectively) and *DQB1* (*Q* = 8.7 × 10^−10^ and *Q* = 1.93 × 10^−17^, respectively) genes, suggesting that the alleles may have etiologic effects through more than just the structure of their respective binding proteins.

### Discussion

Similar to our other GWAS loci, the HLA fIIP risk locus will require follow-up resequencing in a large number of cases and controls to define the independent variant(s) contributing to fIIP risk. Several other small studies have implicated the HLA region in fIIP with little consensus on the alleles responsible [40–43]. Of particular interest given our findings, the DRB1*15:01 allele has been reported to be associated with IPF among a small sample from China [41]. In the time since we conducted the imputation analyses and conducted replication genotyping, more comprehensive imputation panels have been made available. We have conducted further imputation analyses based on the 1000 genomes panel; while there are no additional genome-wide signals in the absence of replication genotyping, there are additional SNPs that would be candidates for follow-up based on the screening approach we used in this study (Additional file 1: Table S12).

Our findings should be understood in the context of fibrotic interstitial lung disease, which is a relatively common manifestation of several immune-related disorders, including scleroderma, rheumatoid arthritis, and systemic lupus erythematosis. Interestingly, the DRB1:15:01 allele has been reported to be protective for development of systemic sclerosis among White and Hispanic subjects [44]. While we explicitly excluded cases with recognized connective tissue disease-associated or other known syndromic forms of fIIP, our findings highlight the concept that auto-immunity may drive the development of fIIP in a subset of patients with this disease. A recent case series indicated that 29 % of biopsy-confirmed IPF patients (that is, those without a characterized connective tissue disease or other known cause of their ILD) who underwent serology testing had a positive serology for auto-immunity [45]. In addition, the DRB1*15 and DQB1*06 alleles have been associated with interstitial lung disease in a sample of rheumatoid arthritis patients in Japan [43].

## Conclusions

We have identified the HLA region as associated with fIIP. Two strongly-linked HLA alleles are associated with fIIP and affect expression of HLA genes in lung tissue, indicating that the potential genetic risk due to HLA alleles may involve gene regulation in addition to altered protein structure. In aggregate, the genome-wide genotyped and imputed SNPs are estimated to account for 35 % of the variability in risk of fIIP, a disease that was previously thought to be idiopathic. Importantly, the HLA locus and other risk loci will facilitate the identification of genes and processes that are involved in the etiology of fIIP that may lead to breakthroughs in disease pathogenesis and intervention. In particular for the HLA locus, linking the putative risk alleles to differential expression in tissue from the lung is one way to narrow the list of expression differences between cases and controls to those that are more likely to be drivers of disease risk as compared to consequences of the disease process. In addition, the association between the putative HLA risk alleles and transcript abundance of HLA genes provides alternative avenues of investigation into the functional role of the alleles.

## Methods

### Study populations, ethics, permission and consent

#### Case definition

We used standard criteria established by the American Thoracic Society/European Respiratory Society [46] to determine diagnostic classification of all patients in the discovery and replication phases. We excluded cases with known explanations for development of fibrotic FIIP including infections, systemic disorders, or relevant exposures (e.g. asbestos). To maximize power and minimize potential confounding by ancestry, we included only non-Hispanic white (NHW) participants that had been recruited as part of existing studies. All subjects gave written informed consent as part of IRB-approved protocols for their recruitment at each site (National Jewish Health, InterMune, Vanderbilt University, National Heart Lung and Blood Institute, Duke University, University of Pittsburgh, University of Texas Southwestern, Centre National de Génotypage, National Institute for Health Research Biomedical Research Unit, Royal Brompton Hospital, University of California San Francisco) and the GWAS study was approved by the National Jewish Health IRB and Colorado Combined Institutional Review Board (COMIRB).

#### GWAS discovery

We genotyped 1914 patients with fIIP from 7 cohorts (familial interstitial pneumonia [*n* = 566], National Jewish Health FIIP population [*n* = 238], InterMune IPF trials [*n* = 720], UCSF [*n* = 66], Vanderbilt University FIIP population [*n* = 105], and the National Heart Lung and Blood Institute Lung Tissue Research Consortium [*n* = 219]) and compared them to genotypes from 4683 out-of-study controls. After genotype quality control, we included 1616 cases in analyses.

A family with familial interstitial pneumonia (FIP) is defined by the presence of at least 2 cases of definite or probable IIP in individuals who are 3^rd^ degree relatives or closer. Recruitment of families based at three major referral centers (Vanderbilt University, Duke University and National Jewish Health) has been ongoing since 1999. We included only 1 fIIP case among first degree relatives. The National Jewish Health fIIP cohort consists of patients with sporadic fIIP who were clinically evaluated and enrolled at National Jewish Health as part of ongoing natural history studies. Details of the recruitment criteria for the cases from the Intermune IPF γ-Interferon Intervention Trial have been described in detail [47]. Briefly, eligible patients had IPF, were 40 to 79 years old with clinical symptoms for at least 3 months and evidence of disease progression within the previous 12 months. We included all available cases regardless of treatment assignment. The National Heart Lung and Blood Institute Lung Tissue Research Consortium (NHLBI LTRC) was established to provide lung tissue and DNA for the research community. We included DNA from those subjects with a diagnosis of fIIP after receiving permission from the NHLBI LRTC.

We used de-identified control genotypes generated at Centre d’Étude du Polymorphisme Humain (CEPH) as part of other studies. Potential controls were those who self-reported NHW, had been genotyped on the same platform as our cases, and were appropriately approved for use as controls in other studies. We selected a subset of controls, corresponding to approximately 3 controls for 1 case, based on genetic similarity to the cases which passed our genotyping quality control thresholds as previously described [32].

#### Replication

We genotyped a total of 978 NHW fIIP cases and 2052 NHW controls for replication of the top SNPs from the GWAS. The replication controls were a subset (*n* = 1926) of the controls from the Chronic Obstructive Pulmonary Disease (COPD) Gene Study [48] and 126 controls from the University of Pittsburgh. We selected controls to be frequency matched to the replication cases based on age and gender. To exclude putative duplicate cases within the replication and between the GWAS and replication case groups, we calculated the kinship coefficient for each pair of individuals using the 329 SNPs constituting the combined replication genotyping from the original (181 SNPs) and imputation (148 SNPs) GWAS studies. There were no duplicate individuals within the GWAS; we removed 39 replication cases with kinship coefficient > .45 with either a GWAS case or another replication case. After duplicate removal and genotype quality control (see below), we included 878 cases and 2017 controls in any analyses that included replication samples.

#### Expression

We measured gene expression on a subset of Lung Tissue Research Consortium (*n* = 50) and National Jewish Health fIIP cases from the GWAS (*n* = 37) and National Jewish Health controls (*n* = 70). Whole-lung samples were obtained from International Institute for the Advancement of Medicine (Edison, NJ). Eligible cases and controls had sufficient RNA from lung tissue biopsy available for assay; cases with IPF were preferentially chosen over other fIIP diagnoses. Treatment status of IPF cases were not available for the 37 National Jewish Health cases; of the 50 LTRC IPF patients included in the RNA expression analysis, 33 (66 %) were on systemic steroid treatment, 14 (28 %) were not on systemic steroid treatment and 3 (6 %) did not have information on treatment status.

### Genotyping

Genome-wide genotyping was carried out at CEPH using the Illumina 660 Quad beadchip (www.illumina.com). Details of the quality control and experiments have been reported in detail previously [32]. Replication genotyping was carried out at the Biomedical Genomics Center at the University of Minnesota. For validation and replication genotyping of imputation association results, we attempted to genotype 167 SNPs with adjusted imputation P-values less than 0.0001 (see Statistical Analyses) in 1027 independent cases and 2052 replication controls. In addition, to allow follow-up joint statistical tests (using raw genotypes from both GWAS cases and replication cases and controls) and validate the imputation genotypes, we also genotyped a large subset of GWAS cases (*N* = 1578). Details of the validation assays are described below. After genotyping quality control, we included 878 cases and 2017 controls in the replication, meta- and joint analyses and 1498 of the GWAS cases in the joint analyses.

Prior to genotyping, all samples were quality controlled by real-time Q-PCR quantitation (“QC1”) and uniplex genotyping using Taqman (“QC2”). Samples that failed QC1 or QC2, although carried forward through genotyping, were later removed from analysis.

Validation genotyping was accomplished with a combination of multiplexed (Sequenom iPLEX) and uniplex (Taqman) assays. First, assay design for multiplexed Sequenom iPLEX genotyping was performed on an input set of 167 SNPs (Additional file 1: Table S2), using a combination of web-based (AssayDesigner Suite, www.sequenom.com) and desktop (AssayDesigner) software tools (Sequenom, San Diego). Sequenom iPLEX genotyping is based on multiplexed locus-specific PCR amplification, multiplexed single-based extension (SBE) from locus-specific amplicons, and multiplexed resolution of SBE products base calling using matrix-assisted laser desorption/ionization time-of-flight (MALDI-TOD) mass spectrometry.

Primers for the Sequenom assay were purchased from IDT (Coralville, Iowa), and all steps of the iPLEX procedure were carried out using reagents and methods from Sequenom (San Diego, CA) according to the manufacturer’s instructions. Reactions were carried out in 384-well plates and analyzed using the Sequenom MassARRAY Analyzer 4 system with iPLEX Gold reagents and SpectroCHIP arrays. Results were analyzed using a combination of commercial software (Typer 4, Sequenom) and custom tools for data management. Of 167 assays in 6 multiplexes, 148 were successful in generating usable genotyping data.

### Gene expression

Total RNA was isolated from approximately 30 mg of snap-frozen or RNA-later preserved lung tissue using the Ambion mirVana kit (Life Technologies). RNA concentration was determined by Nanodrop ND-1000 (Thermo Scientific) and RNA integrity was determined using the 2100 Bioanalyzer (Agilent). cDNA single strand conversions were performed using the Superscript III.

### Statistical analyses

Statistical analyses for the discovery GWAS based on observed genotypes, including selection of out-of-study controls, removal of genetic outliers, SNP quality control and tests for association have been described previously in detail [32].

#### Imputation

We imputed genotypes using the combined case and control discovery samples for all HapMap SNPs not on the Illumina 660 Quad beadchip across the entire genome. We used the multi-population reference panel data from HapMap3 for pre-phasing using Shapeit with appropriate default parameters [49, 50]. We performed imputation using IMPUTE2 [34, 35] and tested for association at only those SNPs with imputation information as measured by .info > 0.5 using SNPTEST (v2; [36]) with multiple Newton–Raphson iterations to estimate parameters. We adjusted for sex in all models. We computed the inflation factor across all the SNPs as the ratio of the median of the observed 1 degree of freedom chi-square test statistic obtained from the SNPTEST p-value to the theoretical median of 1 degree of freedom chi-square random variable. We adjusted the imputed p-values based on a comparison between the imputation test statistics and exact mixed model test statistics (from GEMMA [37]) for SNPs that were genotyped. For 439,827 SNPs that were genotyped and also imputed with .info > 0.5 (see Additional file 1: Figure S4 for distribution of .info scores), we computed the inflation factor as the ratio of the median of the observed 1 degree of freedom chi-square test statistics obtained from SNPTEST p-value from genotyped snps and the theoretical median of 1 degree of freedom chi-square random variable. We then computed adjusted imputed p-values across the genome by dividing the observed chi-square test statistic by the inflation factor. We compared the distribution of these adjusted p-values obtained under the additive model to that expected under the null hypothesis of no association across the genome and report the quantile-quantile (Q-Q plot) and genomic inflation factor (λ) to verify the absence of systematic biases. We selected a subset of SNPs with an adjusted imputation p-value <0.0001 for follow-up in the replication populations. Within novel loci, we selected the top SNP within a 500 kb window, and then only selected another SNP if that SNP had an estimated *r*^*2*^ < 0.8 with the top SNP. We continued this process until all remaining SNPs had *r*^*2*^ ≥ 0.8 with at least one selected SNP. Within previously-known loci, we used the top SNP from our original GWAS, and then only selected another SNP if that SNP had an estimated *r*^*2*^ < .5 with the top SNP.

#### Replication association

We tested for association between each replication SNP and fIIP under an additive model (on the log-odds scale) in the replication cases and controls and estimated odds ratios and 95 % confidence intervals using PLINK. For SNPs with minor allele frequency < 5 %, we computed a permutation-based p-value with 100,000 permutations. The p-values were used in the meta-analysis of the GWAS and replication cohorts; based on the low level of imbalance in our study and the total count of minor alleles for the SNPs we tested, the meta-analysis should have appropriate maintenance of the type I error rate [51].

#### Meta-analysis

To obtain a joint measure of association between each of the 148 successfully genotyped SNPs in the replication set and fIIP, we performed a meta-analysis of the GWAS and replication results using the p-values from each analysis using METAL (http://www.sph.umich.edu/csg/abecasis/metal/). We used the weighted inverse normal method with weight equal to the square root of the total sample size in the *i*^*th*^ study. SNPs with *P*_Meta_ < 5 × 10^−8^ were considered genome-wide statistically significant. We created a locus-specific plot [52] of the discovery GWAS imputation results for the newly-identified chromosome 6 region that was genome-wide significant in the meta-analysis.

#### Multi-SNP models

To assess the independence of effects of the genome-wide significant SNPs from the meta-analysis, we used logistic regression models within each locus using the combined case group (GWAS and replication) and the replication controls. Specifically, within each locus with a genome-wide significant SNP, we tested for association between fIIP and each of the other validation panel SNPs within that locus after adjusting for the most significantly associated SNP in that locus (on chromosome 11p15, we adjusted for rs35705950).

#### HLA allele imputation and statistical analyses

Classical human leukocyte antigen (HLA) alleles at *HLA-A*,-*B*,-C*,-DPB1*,*-DQA1*,*-DQB1*, and *-DRBI* were imputed using the R-package HIBAG [38]. HIBAG uses an ensemble classifier and bagging techniques to arrive at an average posterior probability. The HIBAG-provided European-Ancestry reference panel was utilized for imputation. To account for imputation uncertainty, the allele dosage was utilized for all analyses. To filter-out extremely low frequency alleles, potentially yielding unstable estimates, a minimum best guess allele count of 10 was required in either the cases or controls for all alleles modeled. We tested for association between each of the alleles and fIIP using logistic regression, adjusting for the top three principal components of SNP variation, age and sex. We conducted a step-wise regression across all the imputed alleles, removing variables not independently associated with fIIP at the .01 level after adjustment for the other alleles in the model. Finally, we tested for association between pulmonary fibrosis and each of DRB1*15:01 and DQB1*06:02 adjusted for rs7887 and the first three principal components, age and sex, to determine the independence of effects.

#### RNA-seq expression analyses

After verifying that our amplicon-based targeted RNA-seq protocol achieved excellent enrichment for the small target regions, we used these regions (one per gene) to estimate gene expression. We used RSEM to assign reads to targeted regions using an EM algorithm. We removed one sample with fewer than 20,000 total reads and nine genes in the HLA region with fewer than 25 reads in more than 25 samples. We tested for differential gene expression in the lung between 87 cases and 70 controls with EdgeR^36^, using a negative binomial regression for the RNA-seq counts for each gene, adjusting for age and sex. A q-value < .05 was considered statistically significant. We used negative binomial regression, adjusting for age, sex, and total number of reads, to test for association between expression and rs7887 allele separately for cases and controls; we tested for association between alleles DRB1*15:01 and DQB1*06:02 and expression among cases only since these alleles could not be imputed for these controls without genome-wide SNP data.

## Abbreviations

CEPH, Centre d’Étude du Polymorphisme Humain; FIIP, Fibrotic Idiopathic Interstitial Pneumonia; FIP, Familial Interstitial Pneumonia; GWAS. Genome-wide Association Study; HIBAG, HLA genotype Imputation with attribute BAGging; HLA, Human Leukocyte Antigen; NHLBI-LTRC, National Heart Lung and Blood Institute - Lung Tissue Research Consortium; NHW, Non-Hispanic White.

## Additional file

Additional file 1: Tables S1. 205 SNPs Associated with IIP at 5 × 10^−8^ in Imputation Analysis. **Table S2:** SNPs with 5×10^−8^ < P_imputed-adjusted_ < .0001. **Table S3:** GWAS-Significant SNPs after Meta-Analysis in All Regions (Most Significant in Region in Bold Type). **Table S4:** Table of 70 SNPs (46 original, 24 from imputation) Adjusted for Top SNP in Region if More than One SNP in Region. Top SNP Defined in Bold Type in Table S3^a^. All Cases (Discovery and GWAS) Compared to Replication Controls. **Table S5:** HLA Allele Imputation Accuracy Summary. **Table S6:** HLA Allele Association with IIP. **Table S7:** Chromosome 6p21 genes studied via RNA-seq. **Table S8:** Differential Expression by Case–control Status. **Table S9:** Differential Expression by Genotype at rs7887 Among Controls. **Table S10:** Differential Expression by Imputed Number of Copies of DRB1*15:01 Among Cases (RNA-seq). **Table S11:** Differential Expression by Imputed Number of Copies of DQB1*06*02 Among Cases (RNA-seq). **Table S12:** 214 Novel SNPs Meeting Carry-forward Threshold at *P* < 1 × 10^−4^ in Imputation Analysis using 1000 Genomes Reference. **Figure S1:** Q-Q plots of *P*-values from SNPTest using Imputed Dosage for a) Genotyped SNPs and b) Imputed SNPs Prior to Genomic Control Adjustment Based on Genotyped SNP *P*-values from GEMMA analysis (see manuscript statistical methods for details). **Figure S2:** Q-Q plot of imputed (adjusted) p-values (inflation factor = 1.04) after genomic control correction. **Figure S3:** Imputation GWAS Results Fig. 1: Imputation GWAS results with 1616 cases and 4683 controls under additive model. SNPs above red line were genome-wide significant at *P* < 5 × 10^-8.^ A subset of these SNPs and SNPs between red and blue lines, corresponding to 5 × 10^−8^ < *P*-value < .0001, were selected for follow-up and genotyped in 878 cases and 2017 controls. **Figure S4:** Histogram of. INFO Scores from SNPTest. Only SNPs with .INFO >0.5 Included. **Figure S5:** Comparison of SNPTest *p*-values after genomic control (see manuscript statistical methods) and GEMMA dosage-values. GEMMA dosage p-values computed after initial study complete. (DOCX 8917 kb)
