# Supplementary material for: Genome-wide imputation study identifies novel HLA locus for pulmonary fibrosis and potential role for auto-immunity in fibrotic idiopathic interstitial pneumonia
Source: BMC Genet. 2016 Jun 7;17:74. doi: 10.1186/s12863-016-0377-2 (PMC4895966; doi:10.1186/s12863-016-0377-2)
Supplement: Additional file 1: Tables S1. — 205 SNPs Associated with IIP at 5 × 10^−8^ in Imputation Analysis. Table S2: SNPs with 5×10^−8^ < Pimputed-adjusted < .0001. Table S3: GWAS-Significant SNPs after Meta-Analysis in All Regions (Most Significant in Region in Bold Type). Table S4: Table of 70 SNPs (46 original, 24 from imputation) Adjusted for Top SNP in Region if More than One SNP in Region. Top SNP Defined in Bold Type in Table S3a. All Cases (Discovery and GWAS) Compared to Replication Controls. Table S5: HLA Allele Imputation Accuracy Summary. Table S6: HLA Allele Association with IIP. Table S7: Chromosome 6p21 genes studied via RNA-seq. Table S8: Differential Expression by Case–control Status. Table S9: Differential Expression by Genotype at rs7887 Among Controls. Table S10: Differential Expression by Imputed Number of Copies of DRB1*15:01 Among Cases (RNA-seq). Table S11: Differential Expression by Imputed Number of Copies of DQB1*06*02 Among Cases (RNA-seq). Table S12: 214 Novel SNPs Meeting Carry-forward Threshold at P < 1 × 10^−4^ in Imputation Analysis using 1000 Genomes Reference. Figure S1: Q-Q plots of P-values from SNPTest using Imputed Dosage for a) Genotyped SNPs and b) Imputed SNPs Prior to Genomic Control Adjustment Based on Genotyped SNP P-values from GEMMA analysis (see manuscript statistical methods for details). Figure S2: Q-Q plot of imputed (adjusted) p-values (inflation factor = 1.04) after genomic control correction. Figure S3: Imputation GWAS Results Fig. 1: Imputation GWAS results with 1616 cases and 4683 controls under additive model. SNPs above red line were genome-wide significant at P < 5 × 10^-8^. A subset of these SNPs and SNPs between red and blue lines, corresponding to 5 × 10^−8^ < P-value < .0001, were selected for follow-up and genotyped in 878 cases and 2017 controls. Figure S4: Histogram of. INFO Scores from SNPTest. Only SNPs with .INFO >0.5 Included. Figure S5: Comparison of SNPTest p-values after genomic control (see manuscript statistical methods) and GEMMA dosage-val [file 12863_2016_377_MOESM1_ESM.docx]

**SUPPLEMENTARY INFORMATION**

**Genome-wide imputation study identifies novel HLA locus for pulmonary fibrosis and potential role for auto-immunity in idiopathic interstitial pneumonia**

Tasha E. Fingerlin^1,2^, Weiming Zhang^2^, Ivana V. Yang^3^, Hannah C. Ainsworth^4^, Pamela H. Russell^5^, Rachel Z. Blumhagen^1,2^, Marvin I. Schwarz^3^, Kevin K. Brown^1^, Mark P. Steele^6^, James E. Loyd^6^, Gregory P. Cosgrove^1^, David A. Lynch^1^, Steve Groshong^1^, Harold R. Collard^7^, Paul J. Wolters^7^, Williamson Z. Bradford^8^, Karl Kossen^8^, Scott D. Seiwert^8^, Roland M. du Bois^9,10^, Christine Kim Garcia^11^, Megan S. Devine^11^, Gunnar Gudmundsson^12^, Helgi J. Isaksson^12^, Naftali Kaminski^13^, Yingze Zhang^13^, Kevin F. Gibson^13^, Lisa H. Lancaster^6^, Toby M. Maher^9,10^, Philip L. Molyneaux^9,10^, Athol U. Wells^9,10^, Miriam F. Moffatt^9,10^, Moises Selman^14^, Annie Pardo^15^, Dong Soon Kim^16^, James D. Crapo^1^, Barry J. Make^1^, Elizabeth A. Regan^1^, Dinesha S. Walek^17^, Jerry J. Daniel^17^, Yoichiro Kamatani^18^, Diana Zelenika^19^, Elissa Murphy^3^, Keith Smith^3^, David McKean^3^, Brent S. Pedersen^3^, Janet Talbert^3^, Julia Powers^3^, Cheryl R. Markin^6^, Kenneth B. Beckman^17^, Mark Lathrop^18,19^, Brian Freed^3^, Carl D. Langefeld^4^, David A. Schwartz^1,3,20^

^1^ Center for Genes, Environment and Health, National Jewish Health, Denver, CO

^2^ Department of Biostatistics and Informatics, University of Colorado Denver, Aurora, CO

^3^ Department of Medicine, School of Medicine, University of Colorado Denver Aurora, CO

^4^ Center for Public Health Genomics and Department of Biostatistical Sciences, Wake Forest School of Medicine, Winston-Salem NC

^5^  Department of Biochemistry and Molecular Genetics, School of Medicine, University of Colorado Denver, Aurora, CO

^6^ Department of Medicine, Vanderbilt University School of Medicine, Nashville, TN

^7^ Department of Medicine, University of California San Francisco, San Francisco, CA

^8^ InterMune, Brisbane, CA

^9^ National Heart and Lung Institute, Imperial College, London, UK

^10^ National Institute for Health Research Biomedical Research Unit, Royal Brompton Hospital, London, UK

^11^ Department of Medicine, University of Texas Southwestern, Dallas, TX

^12^ Landspitali University Hospital and University of Iceland Faculty of Medicine, Reykjavik, Iceland

^13^ Department of Medicine, University of Pittsburgh, Pittsburgh, PA

^14^ Instituto Nacional de Enfermedades Respiratorias, Mexico City, Mexico

^15^ Universidad Nacional Autonoma de Mexico, Mexico City, Mexico

^16^ Asan Medical Center, University of Ulsan College of Medicine, Seoul, Korea

Department of Biostatistics, Wake Forest University, Winston-Salem NC

^17^ University of Minnesota Genomics Center, University of Minnesota; Minneapolis, MN

^18^ Fondation Jean Dausset, Centre d’Étude du Polymorphisme Humain, Paris, France

^19^ Commissariat à l’Energie Atomique, Institut Génomique, Centre National de Génotypage, Evry, France

^20^ Department of Immunology, School of Medicine, University of Colorado Denver, Aurora, CO

**Supplementary Methods**

DNA preparation, storage, and quality control

Genomic DNA was isolated from both whole blood and biopsied lung tissue on either the Autopure LS (Qiagen) or Qiacube (Qiagen) automation platform, respectively. Prior to extraction on the Qiacube using the DNAeasy kit, fibrotic lung tissues were first homogenized using Lysing Matrix D tubes and a FastPrep-24 benchtop homogenizer (MPBiomedicals). Following isolation, all DNA was assayed for concentration and purity on the NanoDrop ND-1000 Spectrophotometer. Samples were excluded if DNA was < 50ng/ul or had an A260/A280 ratio outside of the 1.7-2.0 range.

For GWAS genotyping, prior to submission to the Centre National de Genotypage at CEPH, all samples were re-quantified using the Quant-iT PicoGreen dsDNA Assay Kit (Invitrogen), normalized with 1xTE, and aliquotted into individually barcoded screw-cap tubes. Due to volume limitations with liquid handling robots, an absolute minimum quantity for submission to the CNG was 30ul at 50ng/ul. If samples did not meet this minimum quantity, an alternate extraction was performed or the sample was withheld from the study.

For replication genotyping, upon receipt, samples were transferred into 96-well robotics compatible plates, quantified with PicoGreen, and normalized with 1xTE. 400ng of DNA was submitted for each member of the GWAS and the replication cohorts sent for replication genotyping. In an effort to minimize confounding by batch effects, samples were aliquotted into 96-well plates in a randomized fashion across all cohorts with two duplicates per plate using the Tecan Evo200 liquid handling robot.

**Supplementary Tables and Figures**

**Tables:**

**S1: 205 SNPs Associated with IIP at 5x10^-8^ in Imputation Analysi**s.

| **Chr** | **SNP** | **Position** | **Minor Allele** | **Case freq^a^** | **Control freq^b^** | **P Imputed^c^** | **Rep Case freq^d,e^** | **Rep Control freq^d,f^** | **P Replication^d,g^** | **OR**  **replication only^d,h^** | **P Meta^d,i^** |  |
| --- | --- | --- | --- | --- | --- | --- | --- | --- | --- | --- | --- | --- |
| 3 | rs12638862 | 170960200 | G | 0.31 | 0.26 | 3.04e-08 |  |  |  |  |  |  |
| 3 | rs12630450 | 170962898 | G | 0.32 | 0.27 | 1.24e-08 |  |  |  |  |  |  |
| 3 | rs12696304 | 1.71E+08 | G | 0.33 | 0.27 | 4.15e-09 | 0.32 | 0.26 | 3.84e-06 | 1.35 (1.19, 1.53) | 8.22e-14 |  |
| 3 | rs9860874 | 170968965 | A | 0.32 | 0.27 | 7.86e-09 |  |  |  |  |  |  |
| 3 | rs3821383 | 170972640 | G | 0.32 | 0.26 | 1.01e-08 |  |  |  |  |  |  |
| 3 | rs1920120 | 170984874 | C | 0.32 | 0.26 | 1.17e-08 |  |  |  |  |  |  |
| 3 | rs2141595 | 170986126 | T | 0.32 | 0.27 | 7.28e-09 |  |  |  |  |  |  |
| 3 | rs7625734 | 170991609 | C | 0.32 | 0.27 | 2.97e-08 |  |  |  |  |  |  |
| 3 | rs7633750 | 170991938 | A | 0.32 | 0.27 | 1.78e-08 |  |  |  |  |  |  |
| 3 | rs10936601 | 171011143 | T | 0.32 | 0.27 | 1.62e-08 |  |  |  |  |  |  |
| 3 | rs9831661 | 171011217 | T | 0.32 | 0.27 | 1.64e-08 |  |  |  |  |  |  |
| 4 | rs2904259 | 90104737 | C | 0.44 | 0.51 | 1.68e-08 |  |  |  |  |  |  |
| 5 | rs10069690 | 1332790 | T | 0.23 | 0.28 | 1.73e-10 | 0.19 | 0.25 | 1.31e-06 | 0.7 (0.61, 0.81) | 1.19e-15 |  |
| 5 | rs2853672 | 1345983 | A | 0.53 | 0.48 | 1.53e-08 |  |  |  |  |  |  |
| 6 | rs1358904 | 7509460 | T | 0.35 | 0.29 | 2.47e-09 |  |  |  |  |  |  |
| 7 | rs6974373 | 99428829 | G | 0.46 | 0.4 | 2.25e-09 |  |  |  |  |  |  |
| 7 | rs6963345 | 99456542 | A | 0.45 | 0.38 | 3.47e-12 |  |  |  |  |  |  |
| 11 | rs10902073 | 1050934 | A | 0.43 | 0.35 | 3.09e-13 |  |  |  |  |  |  |
| 11 | rs10794278 | 1051718 | T | 0.43 | 0.35 | 4.24e-13 |  |  |  |  |  |  |
| 11 | rs10794279 | 1051781 | T | 0.43 | 0.35 | 4.22e-13 |  |  |  |  |  |  |
| 11 | rs10794280 | 1052867 | T | 0.43 | 0.35 | 1.2e-13 |  |  |  |  |  |  |
| 11 | rs10794284 | 1057011 | A | 0.44 | 0.36 | 2.08e-13 |  |  |  |  |  |  |
| 11 | rs10902088 | 1077972 | T | 0.15 | 0.2 | 2.82e-08 | 0.18 | 0.19 | 0.2 | 0.91 (0.78, 1.05) | 1.39e-007 |  |
| 11 | rs2672794 | 1197581 | T | 0.36 | 0.42 | 3.5e-10 | 0.29 | 0.38 | 4.57e-09 | 0.69 (0.6, 0.78) | 1.77e-17 |  |
| 11 | rs2672812 | 1205948 | A | 0.43 | 0.5 | 2.12e-09 |  |  |  |  |  |  |
| 11 | rs2075859 | 1207064 | T | 0.3 | 0.37 | 7.93e-12 | 0.3 | 0.36 | 1.82e-05 | 0.76 (0.67, 0.86) | 7.86e--16 |  |
| 11 | rs2014486 | 1215379 | G | 0.44 | 0.5 | 4.91e-09 |  |  |  |  |  |  |
| 11 | rs2735733 | 1218216 | T | 0.38 | 0.45 | 9.33e-09 | 0.4 | 0.44 | 4.4e-03 | 0.84 (0.75, 0.95) | 2.41e-10 |  |
| 11 | rs4963059 | 1229434 | T | 0.26 | 0.31 | 6.09e-09 | 0.25 | 0.31 | 2.75e-05 | 0.75 (0.66, 0.86) | 7.6e-13 |  |
| 11 | rs5744034 | 1252813 | G | 0.25 | 0.17 | 2.46e-18 |  |  |  |  |  |  |
| 11 | rs3168046 | 1253225 | T | 0.53 | 0.46 | 8.03e-12 | 0.52 | 0.44 | 7.8e--08 | 1.38 (1.23, 1.56) | 3.81e-18 |  |
| 11 | rs3750920 | 1266532 | T | 0.5 | 0.44 | 5.79e-10 | 0.51 | 0.44 | 3.56e-06 | 1.32 (1.17, 1.48) | 1.03e-14 |  |
| 11 | rs11023590 | 1332536 | C | 0.34 | 0.4 | 2.53e-09 |  |  |  |  |  |  |
| 11 | rs12792005 | 1586753 | A | 0.06 | 0.04 | 3.57e-09 | 0.06 | 0.04 | 4.4e-05 | 1.72 (1.32, 2.23) | 7.04e-13 |  |
| 11 | rs35678986 | 1599178 | G | 0.05 | 0.02 | 6.16e-13 | 0.04 | 0.02 | 1.0e-05 | 2.08 (1.50, 2.88) | 3.56e-17 |  |
| 15 | rs1984793 | 38486665 | C | 0.3 | 0.36 | 2.44e-09 |  |  |  |  |  |  |
| 15 | rs2289331 | 38487232 | T | 0.3 | 0.36 | 2.73e-09 |  |  |  |  |  |  |
| 15 | rs11070270 | 38495714 | A | 0.3 | 0.36 | 4.05e-09 |  |  |  |  |  |  |
| 15 | rs1001528 | 38501066 | A | 0.42 | 0.49 | 8.46e-10 |  |  |  |  |  |  |
| 15 | rs12914710 | 38501141 | G | 0.3 | 0.36 | 3.48e-09 |  |  |  |  |  |  |
| 15 | rs12914315 | 38501170 | A | 0.3 | 0.36 | 3.51e-09 |  |  |  |  |  |  |
| 15 | rs8034217 | 38508992 | T | 0.3 | 0.36 | 2.28e-09 |  |  |  |  |  |  |
| 15 | rs11070272 | 38512555 | A | 0.3 | 0.36 | 2.5e-09 |  |  |  |  |  |  |
| 15 | rs1453184 | 38516692 | A | 0.4 | 0.46 | 1.04e-08 |  |  |  |  |  |  |
| 15 | rs2169877 | 83886806 | G | 0.33 | 0.28 | 2.84e-08 | 0.31 | 0.3 | 0.22 | 1.08 (0.95, 1.23) | 1.61e-07 |  |
| 17 | rs413778 | 41072668 | G | 0.17 | 0.22 | 3.29e-10 |  |  |  |  |  |  |
| 17 | rs389217 | 41072914 | T | 0.17 | 0.22 | 3.29e-10 |  |  |  |  |  |  |
| 17 | rs439558 | 41073586 | C | 0.17 | 0.22 | 3.67e-10 |  |  |  |  |  |  |
| 17 | rs453997 | 41082844 | T | 0.17 | 0.22 | 3.0e-10 |  |  |  |  |  |  |
| 17 | rs241033 | 41089766 | A | 0.17 | 0.22 | 2.75e-10 |  |  |  |  |  |  |
| 17 | rs241031 | 41090087 | C | 0.17 | 0.22 | 2.98e-10 |  |  |  |  |  |  |
| 17 | rs17760733 | 41102059 | T | 0.17 | 0.22 | 2.94e-10 |  |  |  |  |  |  |
| 17 | rs17687571 | 41105793 | A | 0.17 | 0.22 | 2.94e-10 |  |  |  |  |  |  |
| 17 | rs17687667 | 41109882 | A | 0.17 | 0.22 | 2.92e-10 |  |  |  |  |  |  |
| 17 | rs1724400 | 41111654 | A | 0.2 | 0.25 | 2.55e-08 |  |  |  |  |  |  |
| 17 | rs1635288 | 41112752 | A | 0.2 | 0.25 | 3.69e-08 |  |  |  |  |  |  |
| 17 | rs17687849 | 41115502 | G | 0.17 | 0.22 | 3.08e-10 |  |  |  |  |  |  |
| 17 | rs17761207 | 41118038 | C | 0.17 | 0.22 | 2.61e-10 |  |  |  |  |  |  |
| 17 | rs17688002 | 41118377 | T | 0.17 | 0.22 | 2.44e-10 |  |  |  |  |  |  |
| 17 | rs17688056 | 41119024 | T | 0.17 | 0.22 | 2.6e-10 |  |  |  |  |  |  |
| 17 | rs7207373 | 41126495 | T | 0.19 | 0.24 | 1.31e-08 |  |  |  |  |  |  |
| 17 | rs17688391 | 41127892 | A | 0.17 | 0.22 | 2.59e-10 |  |  |  |  |  |  |
| 17 | rs17688434 | 41128323 | A | 0.17 | 0.22 | 4.19e-10 |  |  |  |  |  |  |
| 17 | rs12150547 | 41131329 | G | 0.17 | 0.22 | 2.59e-10 |  |  |  |  |  |  |
| 17 | rs17688773 | 41133493 | C | 0.17 | 0.22 | 2.6e-10 |  |  |  |  |  |  |
| 17 | rs17762165 | 41134385 | T | 0.17 | 0.22 | 2.54e-10 |  |  |  |  |  |  |
| 17 | rs17688922 | 41135134 | A | 0.17 | 0.22 | 2.68e-10 |  |  |  |  |  |  |
| 17 | rs17688944 | 41135202 | A | 0.17 | 0.22 | 2.69e-10 |  |  |  |  |  |  |
| 17 | rs968027 | 41137033 | T | 0.17 | 0.22 | 3.09e-10 |  |  |  |  |  |  |
| 17 | rs17689104 | 41138275 | G | 0.17 | 0.22 | 4.33e-10 |  |  |  |  |  |  |
| 17 | rs17563501 | 41157478 | T | 0.17 | 0.22 | 2.85e-10 |  |  |  |  |  |  |
| 17 | rs2902662 | 41162708 | A | 0.17 | 0.22 | 3.08e-10 |  |  |  |  |  |  |
| 17 | rs17563599 | 41163726 | C | 0.17 | 0.22 | 3.08e-10 |  |  |  |  |  |  |
| 17 | rs17563787 | 41169023 | G | 0.17 | 0.22 | 3.08e-10 |  |  |  |  |  |  |
| 17 | rs17563800 | 41173230 | T | 0.17 | 0.22 | 2.95e-10 |  |  |  |  |  |  |
| 17 | rs17563827 | 41173993 | A | 0.17 | 0.22 | 2.95e-10 |  |  |  |  |  |  |
| 17 | rs12150672 | 41182408 | A | 0.17 | 0.22 | 2.95e-10 |  |  |  |  |  |  |
| 17 | rs17334894 | 41182980 | A | 0.17 | 0.22 | 2.67e-10 |  |  |  |  |  |  |
| 17 | rs17426195 | 41188138 | A | 0.17 | 0.22 | 2.82e-10 |  |  |  |  |  |  |
| 17 | rs11079724 | 41197680 | T | 0.17 | 0.22 | 3.69e-10 |  |  |  |  |  |  |
| 17 | rs4074462 | 41210994 | T | 0.17 | 0.22 | 4.59e-10 |  |  |  |  |  |  |
| 17 | rs17689471 | 41248753 | C | 0.17 | 0.22 | 3.62e-10 |  |  |  |  |  |  |
| 17 | rs17762769 | 41249183 | A | 0.17 | 0.22 | 3.62e-10 |  |  |  |  |  |  |
| 17 | rs8072451 | 41249496 | T | 0.17 | 0.22 | 3.06e-10 |  |  |  |  |  |  |
| 17 | rs4277389 | 41251434 | G | 0.17 | 0.22 | 3.68e-10 |  |  |  |  |  |  |
| 17 | rs4566211 | 41251477 | A | 0.17 | 0.22 | 3.68e-10 |  |  |  |  |  |  |
| 17 | rs17762954 | 41255567 | T | 0.17 | 0.22 | 3.72e-10 |  |  |  |  |  |  |
| 17 | rs1396862 | 41258778 | A | 0.17 | 0.22 | 3.76e-10 |  |  |  |  |  |  |
| 17 | rs17689824 | 41260178 | T | 0.17 | 0.22 | 3.77e-10 |  |  |  |  |  |  |
| 17 | rs17763086 | 41261262 | G | 0.17 | 0.22 | 3.78e-10 |  |  |  |  |  |  |
| 17 | rs17689882 | 41262609 | A | 0.17 | 0.22 | 7.44e-10 |  |  |  |  |  |  |
| 17 | rs16940665 | 41263677 | C | 0.17 | 0.22 | 3.81e-10 |  |  |  |  |  |  |
| 17 | rs17689918 | 41265869 | A | 0.17 | 0.22 | 4.47e-10 |  |  |  |  |  |  |
| 17 | rs1876829 | 41267224 | C | 0.17 | 0.22 | 4.01e-10 |  |  |  |  |  |  |
| 17 | rs17763533 | 41273970 | C | 0.17 | 0.22 | 3.91e-10 |  |  |  |  |  |  |
| 17 | rs17763596 | 41276990 | T | 0.17 | 0.22 | 3.98e-10 |  |  |  |  |  |  |
| 17 | rs17763634 | 41277534 | C | 0.17 | 0.22 | 3.99e-10 |  |  |  |  |  |  |
| 17 | rs12185268 | 41279463 | G | 0.17 | 0.22 | 4.04e-10 |  |  |  |  |  |  |
| 17 | rs12185235 | 41279483 | T | 0.17 | 0.22 | 5.98e-10 |  |  |  |  |  |  |
| 17 | rs12373123 | 41279853 | C | 0.17 | 0.22 | 4.05e-10 |  |  |  |  |  |  |
| 17 | rs12373168 | 41280117 | C | 0.17 | 0.23 | 1.23e-09 |  |  |  |  |  |  |
| 17 | rs17769490 | 41281385 | A | 0.17 | 0.22 | 3.7e-10 |  |  |  |  |  |  |
| 17 | rs34097347 | 41305238 | A | 0.17 | 0.22 | 4.3e-10 |  |  |  |  |  |  |
| 17 | rs916793 | 41310477 | A | 0.17 | 0.22 | 2.16e-10 |  |  |  |  |  |  |
| 17 | rs4441322 | 41310821 | G | 0.17 | 0.22 | 2.16e-10 |  |  |  |  |  |  |
| 17 | rs17691328 | 41311278 | T | 0.17 | 0.22 | 2.15e-10 |  |  |  |  |  |  |
| 17 | rs17691610 | 41326456 | T | 0.17 | 0.22 | 2.54e-10 |  |  |  |  |  |  |
| 17 | rs1864325 | 41333623 | T | 0.17 | 0.22 | 2.0e-10 |  |  |  |  |  |  |
| 17 | rs2082068 | 41335767 | T | 0.17 | 0.22 | 1.91e-10 |  |  |  |  |  |  |
| 17 | rs17649553 | 41350476 | T | 0.17 | 0.22 | 1.99e-10 |  |  |  |  |  |  |
| 17 | rs17649635 | 41351818 | G | 0.17 | 0.22 | 2.02e-10 |  |  |  |  |  |  |
| 17 | rs17649641 | 41353200 | C | 0.17 | 0.22 | 2.04e-10 |  |  |  |  |  |  |
| 17 | rs17564223 | 41353348 | T | 0.17 | 0.22 | 2.04e-10 |  |  |  |  |  |  |
| 17 | rs17649700 | 41353729 | C | 0.17 | 0.22 | 1.99e-10 |  |  |  |  |  |  |
| 17 | rs1467969 | 41354156 | T | 0.17 | 0.22 | 2.06e-10 |  |  |  |  |  |  |
| 17 | rs17564493 | 41357207 | T | 0.17 | 0.22 | 2.02e-10 |  |  |  |  |  |  |
| 17 | rs17649954 | 41357489 | G | 0.17 | 0.22 | 2.1e-10 |  |  |  |  |  |  |
| 17 | rs17564619 | 41357966 | G | 0.17 | 0.22 | 1.91e-10 |  |  |  |  |  |  |
| 17 | rs17650063 | 41358383 | G | 0.17 | 0.22 | 2.28e-10 |  |  |  |  |  |  |
| 17 | rs17564703 | 41358423 | T | 0.17 | 0.22 | 2.12e-10 |  |  |  |  |  |  |
| 17 | rs17564780 | 41361241 | G | 0.17 | 0.22 | 2.15e-10 |  |  |  |  |  |  |
| 17 | rs17564829 | 41362429 | C | 0.17 | 0.22 | 1.76e-10 |  |  |  |  |  |  |
| 17 | rs12150111 | 41369767 | G | 0.17 | 0.22 | 2.17e-10 |  |  |  |  |  |  |
| 17 | rs4327091 | 41377578 | A | 0.17 | 0.22 | 1.93e-10 |  |  |  |  |  |  |
| 17 | rs242559 | 41381748 | C | 0.18 | 0.24 | 2.49e-09 |  |  |  |  |  |  |
| 17 | rs17571718 | 41388634 | C | 0.17 | 0.22 | 1.75e-10 |  |  |  |  |  |  |
| 17 | rs17571739 | 41388781 | C | 0.17 | 0.22 | 1.74e-10 |  |  |  |  |  |  |
| 17 | rs17571857 | 41391544 | G | 0.17 | 0.22 | 1.68e-10 |  |  |  |  |  |  |
| 17 | rs17650860 | 41394844 | A | 0.17 | 0.22 | 1.35e-10 |  |  |  |  |  |  |
| 17 | rs17650872 | 41395352 | T | 0.17 | 0.22 | 1.61e-10 |  |  |  |  |  |  |
| 17 | rs17650901 | 41395527 | G | 0.17 | 0.22 | 1.61e-10 |  |  |  |  |  |  |
| 17 | rs17572169 | 41401810 | T | 0.17 | 0.22 | 1.54e-10 |  |  |  |  |  |  |
| 17 | rs1800547 | 41407682 | G | 0.17 | 0.22 | 1.53e-10 |  |  |  |  |  |  |
| 17 | rs17651213 | 41407760 | A | 0.17 | 0.23 | 4.06e-10 |  |  |  |  |  |  |
| 17 | rs17572361 | 41407845 | C | 0.17 | 0.22 | 1.44e-10 |  |  |  |  |  |  |
| 17 | rs17651549 | 41417115 | T | 0.17 | 0.22 | 1.5e-10 |  |  |  |  |  |  |
| 17 | rs17572851 | 41419603 | G | 0.17 | 0.22 | 1.51e-10 |  |  |  |  |  |  |
| 17 | rs17572893 | 41420045 | A | 0.17 | 0.22 | 1.5e-10 |  |  |  |  |  |  |
| 17 | rs1052551 | 41424761 | A | 0.17 | 0.22 | 1.2e-10 |  |  |  |  |  |  |
| 17 | rs17573175 | 41426926 | G | 0.17 | 0.22 | 1.2e-10 |  |  |  |  |  |  |
| 17 | rs1052553 | 41429726 | G | 0.17 | 0.22 | 1.22e-10 |  |  |  |  |  |  |
| 17 | rs17652121 | 41429810 | C | 0.17 | 0.22 | 1.2e-10 |  |  |  |  |  |  |
| 17 | rs1078269 | 41431674 | C | 0.17 | 0.22 | 1.2e-10 |  |  |  |  |  |  |
| 17 | rs1078268 | 41431738 | G | 0.17 | 0.22 | 1.21e-10 |  |  |  |  |  |  |
| 17 | rs17652449 | 41444774 | C | 0.17 | 0.22 | 1.52e-10 |  |  |  |  |  |  |
| 17 | rs12150506 | 41446373 | A | 0.18 | 0.23 | 5.87e-11 |  |  |  |  |  |  |
| 17 | rs17652502 | 41450308 | A | 0.17 | 0.22 | 1.53e-10 |  |  |  |  |  |  |
| 17 | rs1052587 | 41458449 | C | 0.17 | 0.22 | 1.53e-10 |  |  |  |  |  |  |
| 17 | rs17574228 | 41460355 | C | 0.17 | 0.22 | 1.53e-10 |  |  |  |  |  |  |
| 17 | rs7350928 | 41463947 | T | 0.17 | 0.22 | 1.53e-10 |  |  |  |  |  |  |
| 17 | rs17574361 | 41464049 | G | 0.17 | 0.22 | 1.53e-10 |  |  |  |  |  |  |
| 17 | rs17652961 | 41464202 | A | 0.17 | 0.22 | 1.53e-10 |  |  |  |  |  |  |
| 17 | rs1076222 | 41465616 | G | 0.17 | 0.22 | 1.54e-10 |  |  |  |  |  |  |
| 17 | rs7350980 | 41466118 | A | 0.17 | 0.23 | 5.93e-10 |  |  |  |  |  |  |
| 17 | rs4597358 | 41466517 | G | 0.17 | 0.23 | 5.31e-10 |  |  |  |  |  |  |
| 17 | rs17574604 | 41467460 | G | 0.17 | 0.22 | 1.6e-10 |  |  |  |  |  |  |
| 17 | rs17653162 | 41467674 | A | 0.17 | 0.22 | 1.6e-10 |  |  |  |  |  |  |
| 17 | rs17574824 | 41470954 | T | 0.17 | 0.22 | 1.49e-10 |  |  |  |  |  |  |
| 17 | rs11079729 | 41471416 | A | 0.17 | 0.22 | 1.42e-10 |  |  |  |  |  |  |
| 17 | rs12150090 | 41471733 | T | 0.17 | 0.22 | 1.61e-10 |  |  |  |  |  |  |
| 17 | rs41437445 | 41472085 | A | 0.17 | 0.22 | 1.61e-10 |  |  |  |  |  |  |
| 17 | rs7221390 | 41472797 | G | 0.17 | 0.23 | 5.81e-10 |  |  |  |  |  |  |
| 17 | rs41382552 | 41474846 | G | 0.17 | 0.22 | 1.61e-10 |  |  |  |  |  |  |
| 17 | rs41374248 | 41476292 | G | 0.17 | 0.23 | 3.44e-10 |  |  |  |  |  |  |
| 17 | rs12150447 | 41483977 | C | 0.17 | 0.22 | 1.78e-10 |  |  |  |  |  |  |
| 17 | rs17575556 | 41491663 | A | 0.17 | 0.22 | 1.75e-10 |  |  |  |  |  |  |
| 17 | rs41384744 | 41492888 | G | 0.17 | 0.22 | 1.91e-10 |  |  |  |  |  |  |
| 17 | rs17575850 | 41500209 | A | 0.17 | 0.23 | 4.13e-10 |  |  |  |  |  |  |
| 17 | rs1117253 | 41505119 | C | 0.17 | 0.22 | 1.78e-10 |  |  |  |  |  |  |
| 17 | rs2066899 | 41511550 | T | 0.17 | 0.23 | 6.21e-10 |  |  |  |  |  |  |
| 17 | rs17659881 | 41513416 | G | 0.17 | 0.22 | 1.8e-10 |  |  |  |  |  |  |
| 17 | rs17659953 | 41515544 | T | 0.17 | 0.22 | 1.81e-10 |  |  |  |  |  |  |
| 17 | rs17660065 | 41518102 | C | 0.17 | 0.22 | 1.82e-10 |  |  |  |  |  |  |
| 17 | rs8080583 | 41518415 | A | 0.17 | 0.23 | 6.28e-10 |  |  |  |  |  |  |
| 17 | rs17660132 | 41521621 | C | 0.17 | 0.22 | 1.83e-10 |  |  |  |  |  |  |
| 17 | rs17660464 | 41533806 | A | 0.17 | 0.22 | 1.86e-10 |  |  |  |  |  |  |
| 17 | rs17577094 | 41543275 | G | 0.17 | 0.22 | 1.95e-10 |  |  |  |  |  |  |
| 17 | rs1468241 | 41551932 | G | 0.17 | 0.22 | 1.95e-10 |  |  |  |  |  |  |
| 17 | rs2532316 | 41569489 | A | 0.17 | 0.22 | 1.94e-10 |  |  |  |  |  |  |
| 17 | rs2141299 | 41596763 | A | 0.17 | 0.22 | 1.77e-10 |  |  |  |  |  |  |
| 17 | rs1881194 | 41604591 | A | 0.17 | 0.22 | 1.78e-10 |  |  |  |  |  |  |
| 17 | rs17585974 | 41604976 | G | 0.14 | 0.18 | 2.75e-09 |  |  |  |  |  |  |
| 17 | rs2532270 | 41605577 | A | 0.17 | 0.22 | 1.82e-10 |  |  |  |  |  |  |
| 17 | rs2532234 | 41628043 | G | 0.17 | 0.22 | 1.75e-10 |  |  |  |  |  |  |
| 17 | rs2732674 | 41636474 | C | 0.15 | 0.2 | 1.09e-09 |  |  |  |  |  |  |
| 17 | rs2696455 | 41639348 | T | 0.15 | 0.19 | 1.98e-09 |  |  |  |  |  |  |
| 17 | rs2469933 | 41641308 | A | 0.17 | 0.22 | 1.69e-10 |  |  |  |  |  |  |
| 17 | rs2463524 | 41682022 | G | 0.15 | 0.19 | 1.04e-09 |  |  |  |  |  |  |
| 17 | rs2532373 | 41684998 | C | 0.15 | 0.2 | 6.42e-10 |  |  |  |  |  |  |
| 17 | rs2732711 | 41706070 | G | 0.17 | 0.22 | 5.57e-11 |  |  |  |  |  |  |
| 17 | rs2957297 | 41723989 | A | 0.14 | 0.18 | 5.56e-09 |  |  |  |  |  |  |
| 17 | rs199436 | 42144468 | G | 0.19 | 0.24 | 4.66e-08 |  |  |  |  |  |  |
| 17 | rs199438 | 42146826 | A | 0.19 | 0.24 | 4.42e-08 |  |  |  |  |  |  |
| 17 | rs199439 | 42148686 | G | 0.16 | 0.21 | 7.79e-10 |  |  |  |  |  |  |
| 17 | rs142167 | 42150418 | G | 0.19 | 0.24 | 3.95e-08 |  |  |  |  |  |  |
| 17 | rs199457 | 42150653 | T | 0.16 | 0.21 | 6.86e-10 |  |  |  |  |  |  |
| 17 | rs199456 | 42153103 | T | 0.16 | 0.21 | 8.04e-10 |  |  |  |  |  |  |
| 17 | rs199453 | 42156130 | T | 0.19 | 0.24 | 3.91e-08 |  |  |  |  |  |  |
| 17 | rs199452 | 42156524 | T | 0.19 | 0.24 | 4.79e-08 |  |  |  |  |  |  |
| 17 | rs199451 | 42156968 | A | 0.16 | 0.21 | 1.07e-09 |  |  |  |  |  |  |
| 17 | rs199448 | 42164185 | G | 0.16 | 0.21 | 1.57e-09 |  |  |  |  |  |  |
| 17 | rs199443 | 42174733 | T | 0.16 | 0.21 | 1.61e-09 |  |  |  |  |  |  |
| 17 | rs199535 | 42177829 | G | 0.16 | 0.21 | 1.55e-09 |  |  |  |  |  |  |
| 17 | rs199534 | 42179380 | G | 0.16 | 0.21 | 2.28e-09 |  |  |  |  |  |  |
| 17 | rs9896243 | 42181223 | G | 0.15 | 0.2 | 3.77e-10 |  |  |  |  |  |  |

**^a^Minor allele frequency among GWAS cases based on imputed counts**

**^b^Minor allele frequency among GWAS controls based on imputed counts**

**^c^P-value from imputation analysis under additive model using discovery GWAS samples**

**^d^Only SNPs sent for replication genotyping have these results; none of the Chromosome 17 SNPs were sent for replication genotyping since all in very high LD with known SNP from original GWAS (Fingerlin et al. 2013).**

**^e^Minor allele frequency among replication cases**

**^f^Minor allele frequency among replication controls**

**^g^P-value from additive model among replication cases and controls**

**^h^Odds ratio and 95% CI among replication cases and controls**

**^i^P-value from meta-analysis of discovery imputation and replication.**

**S2: SNPs with 5x10^-8^<P_imputed-adjusted_<.0001**

| **Chr** | **SNP** | **Position** | **Minor Allele** | **Case freq^a^** | **Control freq^b^** | **P Imputed^c^** | **Rep Case freq^d,e^** | **Rep Control freq^d,f^** | **P Replication^d,g^** | **OR**  **replication only^d,h^** | **P Meta^d,i^** |
| --- | --- | --- | --- | --- | --- | --- | --- | --- | --- | --- | --- |
| 1 | rs11589841 | 11570787 | A | 0.23 | 0.26 | 2.91e-05 | 0.3 | 0.29 | 0.39 | 1.06 (0.93, 1.2) | 3.56e-03 |
| 1 | rs967529 | 39368696 | A | 0.01 | 4.99e-03 | 9.91e-05 |  |  |  |  |  |
| 1 | rs667238 | 39463985 | G | 0.01 | 4.52e-03 | 5.44e-05 | 0.01 | 0.01 | 0.67 | 0.83 (0.39, 1.78) | 2.3e-03 |
| 1 | rs592264 | 39618209 | A | 0.01 | 2.97e-03 | 5.86e-05 | 0.01 | 0.01 | 0.71 | 0.85 (0.39, 1.85) | 2.17e-03 |
| 1 | rs783818 | 39671903 | A | 0.01 | 2.51e-03 | 7.31e-05 |  |  |  |  |  |
| 1 | rs813652 | 39704126 | A | 0.01 | 2.47e-03 | 8.25e-05 |  |  |  |  |  |
| 1 | rs698140 | 39727188 | C | 0.01 | 2.01e-03 | 8.2e-06 | 0.01 | 0.01 | 1.0 | 1.02 (0.48, 2.18) | 2.71e-04 |
| 1 | rs782869 | 39746233 | A | 0.01 | 2.50e-03 | 4.95e-05 |  |  |  |  |  |
| 1 | rs945795 | 75558306 | G | 0.01 | 1.66e-03 | 6.69e-05 | 0.01 | 0.01 | 1.0 | 1.01 (0.45, 2.27) | 1.13e-03 |
| 1 | rs10911738 | 183707166 | T | 0.01 | 0.02 | 1.63e-06 | 0.01 | 0.02 | 0.21 | 0.73 (0.44, 1.19) | 3.42e-06 |
| 1 | rs1998063 | 183712967 | G | 0.01 | 0.02 | 1.71e-05 |  |  |  |  |  |
| 1 | rs7518547 | 196806034 | T | 0.2 | 0.17 | 2.02e-05 | 0.17 | 0.2 | 0.12 | 0.89 (0.76, 1.03) | 9.81e-03 |
| 1 | rs4915303 | 196806651 | C | 0.21 | 0.18 | 4.63e-05 |  |  |  |  |  |
| 1 | rs4844639 | 206329103 | C | 0.24 | 0.28 | 8.52e-05 | 0.25 | 0.28 | 0.06 | 0.88 (0.77, 1.00) | 1.64e-05 |
| 1 | rs10863695 | 206330280 | A | 0.24 | 0.28 | 8.74e-05 |  |  |  |  |  |
| 1 | rs4844641 | 206348471 | C | 0.21 | 0.25 | 2.53e-05 | 0.23 | 0.25 | 0.04 | 0.86 (0.75, 0.99) | 3.27e-06 |
| 1 | rs4844413 | 206409309 | T | 0.13 | 0.16 | 4.73e-05 | 0.13 | 0.15 | 0.1 | 0.87 (0.73, 1.03) | 1.97e-05 |
| 1 | rs28481280 | 245869293 | T | 0.33 | 0.3 | 9.76e-05 | 0.33 | 0.33 | 0.98 | 1.0 (0.88, 1.13) | 1.54e-03 |
| 2 | rs11683666 | 6500273 | C | 0.17 | 0.2 | 9.88e-05 | 0.22 | 0.18 | 1.39e-04 | 1.32 (1.15, 1.52) | 0.33 |
| 2 | rs13401444 | 66485804 | C | 3.05e-03 | 4.43e-04 | 1.11e-05 | 2.31e-03 | 1.52e-03 | 0.71 | 1.34 (0.37, 4.90) | 1.42e-04 |
| 2 | rs17025339 | 97977053 | A | 1.87e-03 | 0.00e+00 | 7.05e-05 | 0.00e+00 | 2.54e-04 | 1.0 | 0.0 (0.0, inf) | 1.17e-03 |
| 2 | rs13389786 | 125848699 | G | 0.18 | 0.21 | 3.76e-05 |  |  |  |  |  |
| 2 | rs983969 | 125857537 | A | 0.18 | 0.21 | 3.6e-05 |  |  |  |  |  |
| 2 | rs13411646 | 125870558 | T | 0.18 | 0.21 | 5.09e-05 |  |  |  |  |  |
| 2 | rs12477582 | 125980403 | T | 0.22 | 0.25 | 1.46e-05 |  |  |  |  |  |
| 2 | rs10172193 | 125983936 | A | 0.22 | 0.25 | 1.46e-05 | 0.25 | 0.24 | 0.39 | 1.06 (0.93, 1.21) | 2.35e-03 |
| 2 | rs12692905 | 170012165 | T | 0.2 | 0.24 | 4.09e-05 | 0.22 | 0.23 | 0.17 | 0.91 (0.79, 1.04) | 3.38e-05 |
| 2 | rs2683450 | 170092868 | A | 0.37 | 0.41 | 9.33e-05 |  |  |  |  |  |
| 3 | rs2303859 | 32553241 | C | 0.03 | 0.02 | 7.21e-05 | 0.02 | 0.03 | 0.91 | 0.98 (0.67, 1.44) | 1.51e-03 |
| 3 | rs17631716 | 44890607 | T | 0.32 | 0.28 | 7.94e-05 |  |  |  |  |  |
| 3 | rs12632450 | 44892739 | G | 0.32 | 0.28 | 5.87e-05 | 0.32 | 0.29 | 0.03 | 1.15 (1.01, 1.30) | 6.68e-06 |
| 3 | rs6440080 | 143568000 | G | 3.33e-03 | 4.13e-04 | 4.94e-05 | 1.74e-03 | 7.62e-04 | 0.11 | 2.00 (0.45, 8.93) | 2.33e-05 |
| 3 | rs6796807 | 143577321 | C | 3.33e-03 | 4.07e-04 | 4.96e-05 | 1.74e-03 | 7.62e-04 | 0.11 | 2.00 (0.45, 8.95) | 2.34e-05 |
| 3 | rs16852327 | 143604834 | C | 3.25e-03 | 3.87e-04 | 5.64e-05 | 1.74e-03 | 7.63e-04 | 0.11 | 2.00 (0.45, 8.93) | 2.61e-05 |
| 3 | rs4462984 | 143623165 | C | 3.03e-03 | 3.71e-04 | 9.42e-05 |  |  |  |  |  |
| 3 | rs13097028 | 170947636 | T | 0.39 | 0.34 | 1.82e-07 | 0.38 | 0.34 | 6.6e-03 | 1.18 (1.05, 1.34) | 5.61e-09 |
| 3 | rs1317082 | 170980279 | G | 0.3 | 0.24 | 7.81e-08 |  |  |  |  |  |
| 3 | rs13069553 | 170990966 | G | 0.29 | 0.24 | 1.77e-07 |  |  |  |  |  |
| 3 | rs3796145 | 171007556 | C | 0.29 | 0.24 | 1.91e-07 |  |  |  |  |  |
| 3 | rs7647824 | 171035075 | G | 0.3 | 0.26 | 6.34e-05 | 0.3 | 0.25 | 3.13e-05 | 1.32 (1.16, 1.51) | 1.43e-08 |
| 3 | rs9833035 | 171036192 | T | 0.3 | 0.26 | 6.74e-05 |  |  |  |  |  |
| 4 | rs2629703 | 86564311 | G | 0.29 | 0.33 | 6.03e-05 | 0.34 | 0.33 | 0.57 | 1.04 (0.92, 1.17) | 3.24e-03 |
| 4 | rs340202 | 86572820 | C | 0.3 | 0.33 | 7.51e-05 |  |  |  |  |  |
| 4 | rs2085600 | 89998932 | A | 0.23 | 0.19 | 1.37e-05 | 0.22 | 0.19 | 1.7e-03 | 1.25 (1.09, 1.45) | 8.18e-08 |
| 4 | rs1379932 | 90019915 | T | 0.26 | 0.21 | 3.2e-07 | 0.27 | 0.21 | 4.39e-06 | 1.38 (1.20, 1.58) | 8.82e-12 |
| 4 | rs2464528 | 90036687 | C | 0.26 | 0.21 | 3.84e-07 |  |  |  |  |  |
| 4 | rs7668636 | 90038330 | C | 0.19 | 0.24 | 9.4e-07 | 0.22 | 0.23 | 0.32 | 0.93 (0.81, 1.07) | 4.74e-06 |
| 4 | rs2609265 | 90045989 | T | 0.26 | 0.22 | 4.71e-07 |  |  |  |  |  |
| 4 | rs2609264 | 90047103 | C | 0.26 | 0.21 | 4.21e-07 |  |  |  |  |  |
| 4 | rs2609262 | 90054461 | A | 0.26 | 0.21 | 3.65e-07 |  |  |  |  |  |
| 4 | rs2609261 | 90054508 | T | 0.26 | 0.21 | 3.66e-07 | 0.27 | 0.21 | 7.58e-07 | 1.42 (1.23, 1.63) | 2.42e-12 |
| 4 | rs2609260 | 90055842 | G | 0.21 | 0.17 | 1.44e-06 | 0.22 | 0.17 | 6.55e-05 | 1.35 (1.16, 1.56) | 4.38e-10 |
| 4 | rs4693976 | 90058863 | C | 0.19 | 0.24 | 1.09e-06 |  |  |  |  |  |
| 4 | rs6835019 | 90066678 | G | 0.19 | 0.24 | 1.09e-06 |  |  |  |  |  |
| 4 | rs1246642 | 90083469 | C | 0.26 | 0.21 | 3.68e-07 |  |  |  |  |  |
| 4 | rs2869966 | 90088101 | T | 0.35 | 0.41 | 6.95e-08 | 0.36 | 0.37 | 0.1 | 0.90 (0.80, 1.02) | 8.15e-08 |
| 4 | rs2045517 | 90089987 | T | 0.35 | 0.41 | 7.6e-08 |  |  |  |  |  |
| 4 | rs1903003 | 90105320 | C | 0.51 | 0.46 | 6.94e-08 |  |  |  |  |  |
| 4 | rs1458557 | 90106568 | G | 0.29 | 0.33 | 8.69e-06 |  |  |  |  |  |
| 4 | rs2178584 | 90110221 | T | 0.29 | 0.33 | 1.28e-05 |  |  |  |  |  |
| 4 | rs7686954 | 90123179 | C | 0.29 | 0.33 | 8.82e-06 |  |  |  |  |  |
| 4 | rs10033484 | 90124495 | A | 0.29 | 0.33 | 9.45e-06 |  |  |  |  |  |
| 4 | rs7697900 | 90127647 | C | 0.29 | 0.33 | 8.27e-06 |  |  |  |  |  |
| 4 | rs6835979 | 90131888 | G | 0.39 | 0.43 | 1.3e-05 | 0.41 | 0.43 | 0.19 | 0.92 (0.82, 1.04) | 1.63e-05 |
| 4 | rs2904261 | 90140097 | G | 0.18 | 0.21 | 7.66e-05 |  |  |  |  |  |
| 4 | rs6852928 | 90145216 | C | 0.36 | 0.41 | 1.96e-06 | 0.36 | 0.38 | 0.16 | 0.92 (0.81, 1.03) | 2.65e-06 |
| 4 | rs1795738 | 90149457 | G | 0.18 | 0.22 | 2.32e-06 | 0.2 | 0.21 | 0.42 | 0.94 (0.82, 1.09) | 1.52e-05 |
| 4 | rs1708670 | 90166277 | G | 0.27 | 0.3 | 2.98e-05 |  |  |  |  |  |
| 4 | rs1795727 | 90193745 | C | 0.18 | 0.21 | 2.9e-05 |  |  |  |  |  |
| 4 | rs12640018 | 90222749 | A | 0.18 | 0.21 | 2.85e-05 |  |  |  |  |  |
| 4 | rs17015027 | 90253709 | G | 0.18 | 0.21 | 3.17e-05 |  |  |  |  |  |
| 4 | rs7684332 | 90294407 | T | 0.42 | 0.46 | 4.41e-05 | 0.43 | 0.45 | 0.14 | 0.92 (0.81, 1.03) | 2.84e-05 |
| 4 | rs2903269 | 102669325 | T | 0.24 | 0.2 | 8.21e-05 |  |  |  |  |  |
| 5 | rs2853677 | 1340194 | C | 0.37 | 0.41 | 8.44e-07 | 0.37 | 0.44 | 3.83e-07 | 0.73 (0.65, 0.83) | 3.59e-12 |
| 5 | rs6554759 | 1370102 | G | 0.15 | 0.18 | 9.47e-05 | 0.15 | 0.16 | 0.29 | 0.92 (0.78, 1.08) | 1.48e-04 |
| 5 | rs10479334 | 104982227 | C | 0.06 | 0.08 | 4.02e-05 |  |  |  |  |  |
| 5 | rs6862436 | 134835282 | A | 0.03 | 0.02 | 8.68e-05 |  |  |  |  |  |
| 6 | rs12207400 | 7501961 | G | 0.19 | 0.16 | 4.36e-05 | 0.2 | 0.16 | 4.04e-04 | 1.31 (1.13, 1.53) | 7.45e-08 |
| 6 | rs3799525 | 7502247 | G | 0.2 | 0.25 | 1.05e-06 | 0.21 | 0.24 | 5.54e-03 | 0.82 (0.72, 0.94) | 2.3e-08 |
| 6 | rs2076296 | 7512969 | C | 0.19 | 0.23 | 6.68e-06 |  |  |  |  |  |
| 6 | rs2076301 | 7515308 | T | 0.19 | 0.23 | 7.63e-06 |  |  |  |  |  |
| 6 | rs926411 | 7516873 | G | 0.19 | 0.23 | 3.14e-06 | 0.19 | 0.22 | 0.01 | 0.84 (0.72, 0.96) | 1.8e-07 |
| 6 | rs4713213 | 29446941 | G | 0.48 | 0.44 | 6.5e-05 | 0.46 | 0.45 | 0.45 | 1.04 (0.93, 1.17) | 2.19e-04 |
| 6 | rs429479 | 29480302 | G | 0.12 | 0.09 | 4.71e-05 |  |  |  |  |  |
| 6 | rs406511 | 29484364 | G | 0.12 | 0.09 | 4.8e-05 |  |  |  |  |  |
| 6 | rs1233480 | 29585393 | A | 0.12 | 0.1 | 4.48e-05 | 0.12 | 0.1 | 0.1 | 1.16 (0.97, 1.39) | 1.94e-05 |
| 6 | rs16899202 | 31374278 | C | 1.72e-03 | 0.00e+00 | 6.6e-05 | 1.16e-03 | 7.61e-04 | 0.21 | 2.01 (0.44, 9.21) | 6.99e-05 |
| 6 | rs4540292 | 31425161 | G | 0.37 | 0.41 | 8.41e-06 |  |  |  |  |  |
| 6 | rs4394274 | 31426143 | A | 0.37 | 0.41 | 8.81e-06 |  |  |  |  |  |
| 6 | rs4394275 | 31426156 | A | 0.22 | 0.25 | 9.94e-05 | 0.24 | 0.25 | 0.62 | 0.97 (0.85, 1.10) | 5.38e-04 |
| 6 | rs614549 | 31948604 | C | 0.34 | 0.38 | 5.74e-05 | 0.32 | 0.38 | 5.87e-05 | 0.78 (0.69, 0.88) | 2.09e-08 |
| 6 | rs605203 | 31954991 | C | 0.35 | 0.3 | 7.82e-05 |  |  |  |  |  |
| 6 | rs589428 | 31956199 | T | 0.35 | 0.3 | 8.05e-05 |  |  |  |  |  |
| 6 | rs486416 | 31964049 | C | 0.35 | 0.3 | 6.84e-05 | 0.36 | 0.31 | 5.56e-04 | 1.24 (1.10, 1.4) | 1.57e-07 |
| 6 | rs535586 | 31968316 | T | 0.34 | 0.3 | 7.42e-05 |  |  |  |  |  |
| 6 | rs7887 | 31972526 | A | 0.32 | 0.36 | 1.18e-05 | 0.31 | 0.37 | 5.84e-05 | 0.78 (0.69, 0.88) | 3.7e-09 |
| 6 | rs2844452 | 31990003 | C | 0.42 | 0.46 | 1.69e-05 | 0.42 | 0.47 | 7.1e-04 | 0.82 (0.73, 0.92) | 4.55e-08 |
| 6 | rs644045 | 31991936 | T | 0.37 | 0.33 | 3.88e-05 | 0.38 | 0.33 | 1.51e-03 | 1.21 (1.08, 1.37) | 2.09e-07 |
| 6 | rs3020644 | 32002605 | G | 0.36 | 0.4 | 1.13e-05 | 0.36 | 0.41 | 6.17e-04 | 0.81 (0.72, 0.92) | 2.68e-08 |
| 6 | rs440454 | 32035321 | A | 0.31 | 0.27 | 6.26e-05 |  |  |  |  |  |
| 6 | rs2280774 | 32036670 | T | 0.3 | 0.34 | 1.11e-05 | 0.29 | 0.34 | 9.57e-04 | 0.81 (0.72, 0.92) | 3.89e-08 |
| 6 | rs419788 | 32036778 | A | 0.31 | 0.27 | 6.05e-05 | 0.31 | 0.27 | 0.02 | 1.17 (1.03, 1.32) | 3.35e-06 |
| 6 | rs410851 | 32044647 | T | 0.3 | 0.26 | 7.09e-05 |  |  |  |  |  |
| 6 | rs6902493 | 32130876 | T | 0.29 | 0.34 | 1.64e-05 |  |  |  |  |  |
| 6 | rs7766862 | 32140985 | A | 0.29 | 0.34 | 1.28e-05 |  |  |  |  |  |
| 6 | rs3130287 | 32158522 | C | 0.16 | 0.13 | 4.95e-05 |  |  |  |  |  |
| 6 | rs2071293 | 32170665 | A | 0.29 | 0.33 | 4.77e-05 |  |  |  |  |  |
| 6 | rs17421624 | 32174155 | C | 0.29 | 0.33 | 4.87e-05 |  |  |  |  |  |
| 6 | rs3130342 | 32188124 | A | 0.16 | 0.13 | 3.28e-05 |  |  |  |  |  |
| 6 | rs3130279 | 32220604 | A | 0.16 | 0.13 | 1.27e-05 |  |  |  |  |  |
| 6 | rs3131283 | 32227876 | T | 0.15 | 0.12 | 2.3e-05 |  |  |  |  |  |
| 6 | rs3130283 | 32246523 | A | 0.16 | 0.13 | 7.33e-06 | 0.15 | 0.13 | 0.12 | 1.14 (0.97, 1.34) | 5.36e-06 |
| 6 | rs3132940 | 32269374 | T | 0.15 | 0.12 | 3.21e-05 |  |  |  |  |  |
| 6 | rs1044506 | 32280043 | T | 0.15 | 0.12 | 2.86e-05 |  |  |  |  |  |
| 6 | rs3131294 | 32288124 | A | 0.15 | 0.12 | 2.51e-05 |  |  |  |  |  |
| 6 | rs9268103 | 32353348 | A | 0.19 | 0.16 | 1.63e-05 | 0.18 | 0.17 | 0.15 | 1.12 (0.96, 1.30) | 1.37e-05 |
| 6 | rs9268148 | 32367505 | G | 0.19 | 0.16 | 1.65e-05 |  |  |  |  |  |
| 6 | rs9268199 | 32386613 | G | 0.2 | 0.17 | 3.89e-05 | 0.18 | 0.18 | 0.67 | 1.03 (0.89, 1.20) | 3.15e-04 |
| 6 | rs6920338 | 32396067 | C | 0.17 | 0.13 | 7.01e-06 |  |  |  |  |  |
| 6 | rs3132958 | 32405879 | A | 0.26 | 0.22 | 7.0e-05 |  |  |  |  |  |
| 6 | rs3132959 | 32406920 | G | 0.26 | 0.22 | 7.0e-05 |  |  |  |  |  |
| 6 | rs6904320 | 32420060 | A | 0.26 | 0.23 | 5.29e-05 |  |  |  |  |  |
| 6 | rs3117119 | 32426588 | G | 0.19 | 0.15 | 1.91e-05 |  |  |  |  |  |
| 6 | rs3132963 | 32428131 | G | 0.19 | 0.15 | 1.98e-05 |  |  |  |  |  |
| 6 | rs3129907 | 32431723 | G | 0.26 | 0.22 | 6.67e-05 |  |  |  |  |  |
| 6 | rs2894249 | 32433813 | C | 0.26 | 0.22 | 7.0e-05 |  |  |  |  |  |
| 6 | rs3129931 | 32443494 | C | 0.26 | 0.22 | 4.52e-05 | 0.25 | 0.23 | 0.03 | 1.16 (1.01, 1.33) | 4.53e-06 |
| 6 | rs3129932 | 32444105 | G | 0.26 | 0.22 | 4.52e-05 |  |  |  |  |  |
| 6 | rs3117111 | 32446378 | C | 0.26 | 0.22 | 4.65e-05 |  |  |  |  |  |
| 6 | rs3117116 | 32474995 | C | 0.17 | 0.13 | 9.15e-08 | 0.15 | 0.13 | 0.04 | 1.19 (1.01, 1.40) | 2.65e-08 |
| 6 | rs3135377 | 32493377 | A | 0.25 | 0.2 | 2.54e-06 |  |  |  |  |  |
| 6 | rs2395165 | 32496122 | C | 0.25 | 0.2 | 2.53e-06 |  |  |  |  |  |
| 6 | rs3135365 | 32497233 | G | 0.24 | 0.2 | 1.92e-06 | 0.23 | 0.2 | 9.68e-03 | 1.20 (1.05, 1.38) | 7.38e-08 |
| 6 | rs9268557 | 32497283 | C | 0.47 | 0.51 | 4.65e-05 | 0.46 | 0.5 | 3.0e-03 | 0.84 (0.75, 0.94) | 4.69e-07 |
| 6 | rs3135352 | 32500884 | C | 0.16 | 0.12 | 4.5e-07 |  |  |  |  |  |
| 6 | rs3135350 | 32500959 | C | 0.16 | 0.12 | 4.42e-07 |  |  |  |  |  |
| 6 | rs3135341 | 32506726 | C | 0.22 | 0.18 | 3.49e-07 |  |  |  |  |  |
| 6 | rs984778 | 32508066 | C | 0.35 | 0.31 | 7.8e-05 |  |  |  |  |  |
| 6 | rs3129868 | 32512355 | A | 0.17 | 0.13 | 1.19e-07 |  |  |  |  |  |
| 6 | rs2395173 | 32512837 | A | 0.35 | 0.31 | 7.73e-05 | 0.35 | 0.31 | 3.69e-03 | 1.20 (1.06, 1.36) | 9.41e-07 |
| 6 | rs3135395 | 32513170 | T | 0.35 | 0.31 | 7.9e-05 |  |  |  |  |  |
| 6 | rs3129883 | 32518115 | T | 0.27 | 0.23 | 7.86e-05 | 0.26 | 0.23 | 0.01 | 1.19 (1.04, 1.35) | 3.09e-06 |
| 6 | rs3129886 | 32518554 | T | 0.27 | 0.23 | 7.99e-05 |  |  |  |  |  |
| 6 | rs3135391 | 32518965 | A | 0.16 | 0.12 | 8.12e-07 |  |  |  |  |  |
| 6 | rs3129888 | 32519704 | G | 0.21 | 0.18 | 5.09e-05 | 0.2 | 0.18 | 0.04 | 1.16 (1.0, 1.34) | 7.39e-06 |
| 6 | rs3135388 | 32521029 | A | 0.16 | 0.12 | 7.55e-07 |  |  |  |  |  |
| 6 | rs3129889 | 32521523 | G | 0.16 | 0.12 | 8.53e-07 |  |  |  |  |  |
| 6 | rs35464393 | 32638176 | T | 0.17 | 0.14 | 1.78e-05 |  |  |  |  |  |
| 6 | rs35366052 | 32639901 | G | 0.17 | 0.13 | 1.59e-05 |  |  |  |  |  |
| 6 | rs9270984 | 32681969 | T | 0.18 | 0.14 | 4.04e-06 |  |  |  |  |  |
| 6 | rs9270986 | 32682038 | A | 0.18 | 0.14 | 3.95e-06 |  |  |  |  |  |
| 6 | rs9271055 | 32683347 | G | 0.18 | 0.14 | 3.88e-06 |  |  |  |  |  |
| 6 | rs9271348 | 32691720 | G | 0.32 | 0.27 | 5.51e-06 | 0.32 | 0.29 | 9.42e-03 | 1.17 (1.04, 1.31) | 1.89e-07 |
| 6 | rs9271775 | 32702306 | C | 0.19 | 0.16 | 1.08e-05 |  |  |  |  |  |
| 6 | rs3748080 | 33695932 | A | 0.15 | 0.12 | 2.42e-05 | 0.13 | 0.11 | 0.03 | 1.22 (1.02, 1.45) | 2.14e-06 |
| 6 | rs2766533 | 35793468 | A | 0.43 | 0.48 | 1.12e-05 | 0.44 | 0.47 | 0.14 | 0.92 (0.82, 1.03) | 8.89e-06 |
| 6 | rs7760300 | 54444045 | G | 4.03e-03 | 5.41e-04 | 2.65e-05 | 2.32e-03 | 2.04e-03 | 0.58 | 1.30 (0.38, 4.48) | 1.76e-04 |
| 6 | rs2081511 | 54537141 | T | 3.41e-03 | 3.21e-04 | 2.56e-05 | 1.16e-03 | 1.27e-03 | 0.92 | 1.02 (0.19, 5.47) | 4.76e-04 |
| 6 | rs12664974 | 78469147 | G | 0.01 | 2.60e-03 | 3.64e-05 | 1.74e-03 | 2.03e-03 | 0.5 | 0.67 (0.17, 2.56) | 2.84e-03 |
| 6 | rs12664392 | 78597723 | T | 0.01 | 1.78e-03 | 8.95e-05 | 1.75e-03 | 1.54e-03 | 0.96 | 0.93 (0.23, 3.79) | 1.52e-03 |
| 6 | rs6935556 | 82261531 | A | 0.01 | 9.51e-04 | 8.35e-07 | 0.01 | 0.01 | 1.0 | 0.96 (0.43, 2.12) | 5.75e-05 |
| 6 | rs2180771 | 82284489 | A | 0.01 | 1.04e-03 | 5.29e-06 |  |  |  |  |  |
| 6 | rs9496769 | 100757108 | T | 0.31 | 0.27 | 1.68e-05 |  |  |  |  |  |
| 6 | rs6910107 | 100771560 | C | 0.31 | 0.27 | 1.37e-05 | 0.29 | 0.27 | 0.23 | 1.08 (0.95, 1.23) | 2.17e-05 |
| 6 | rs9498430 | 101477561 | T | 2.19e-03 | 0.00e+00 | 4.16e-05 | 1.16e-03 | 5.07e-04 | 0.08 | 3.20 (0.43, 23.71) | 1.29e-05 |
| 6 | rs6923980 | 108343458 | G | 2.39e-03 | 1.07e-04 | 1.96e-05 | 1.74e-03 | 5.08e-04 | 0.17 | 2.84 (0.46, 17.61) | 1.83e-05 |
| 7 | rs4540313 | 1815653 | T | 0.5 | 0.46 | 5.07e-06 | 0.48 | 0.44 | 0.05 | 1.12 (1.0, 1.26) | 1.28e-06 |
| 7 | rs7799807 | 1834618 | A | 0.34 | 0.38 | 3.99e-05 | 0.34 | 0.37 | 0.17 | 0.92 (0.81, 1.04) | 3.47e-05 |
| 7 | rs4721090 | 1839610 | C | 0.31 | 0.35 | 4.86e-05 |  |  |  |  |  |
| 7 | rs3889797 | 1844450 | C | 0.46 | 0.42 | 6.92e-05 | 0.43 | 0.42 | 0.76 | 1.02 (0.91, 1.15) | 6.15e-04 |
| 7 | rs6952808 | 1853061 | C | 0.31 | 0.35 | 7.2e-05 |  |  |  |  |  |
| 7 | rs6977733 | 1853251 | A | 0.31 | 0.35 | 6.84e-05 |  |  |  |  |  |
| 7 | rs6957894 | 1853888 | A | 0.31 | 0.35 | 5.45e-05 |  |  |  |  |  |
| 7 | rs10260585 | 1856047 | A | 0.31 | 0.35 | 7.86e-05 |  |  |  |  |  |
| 7 | rs4721121 | 1859091 | C | 0.31 | 0.35 | 8.49e-05 |  |  |  |  |  |
| 7 | rs4610628 | 1869626 | T | 0.32 | 0.36 | 4.99e-05 |  |  |  |  |  |
| 7 | rs2280550 | 1943082 | T | 0.34 | 0.38 | 7.25e-05 | 0.35 | 0.36 | 0.35 | 0.94 (0.84, 1.07) | 1.6e-04 |
| 7 | rs7804190 | 1960425 | A | 0.4 | 0.37 | 7.76e-05 | 0.38 | 0.37 | 0.38 | 1.05 (0.94, 1.19) | 1.91e-04 |
| 7 | rs1203577 | 40863529 | C | 2.42e-03 | 2.47e-04 | 2.03e-05 | 3.48e-03 | 1.02e-03 | 0.02 | 3.59 (0.98, 13.1) | 1.39e-06 |
| 7 | rs6962405 | 99395153 | C | 0.45 | 0.41 | 1.59e-05 | 0.45 | 0.42 | 0.04 | 1.13 (1.0, 1.27) | 2.78e-06 |
| 7 | rs2525556 | 99415680 | C | 0.45 | 0.39 | 1.23e-07 |  |  |  |  |  |
| 7 | rs2285886 | 99464231 | C | 0.26 | 0.3 | 2.03e-05 |  |  |  |  |  |
| 7 | rs7803317 | 99475780 | T | 0.33 | 0.29 | 4.06e-05 |  |  |  |  |  |
| 7 | rs4727445 | 99477336 | T | 0.33 | 0.29 | 2.54e-05 | 0.3 | 0.3 | 0.68 | 0.97 (0.86, 1.1) | 1.36e-03 |
| 7 | rs11558475 | 99492536 | G | 0.33 | 0.29 | 4.46e-05 |  |  |  |  |  |
| 7 | rs11558476 | 99492625 | A | 0.33 | 0.29 | 2.81e-05 |  |  |  |  |  |
| 7 | rs12532238 | 99501452 | G | 0.33 | 0.29 | 3.89e-05 |  |  |  |  |  |
| 7 | rs6592 | 99506043 | T | 0.33 | 0.29 | 3.05e-05 |  |  |  |  |  |
| 7 | rs12673441 | 99508264 | T | 0.29 | 0.25 | 1.72e-06 | 0.25 | 0.26 | 0.79 | 0.98 (0.86, 1.12) | 1.74e-04 |
| 7 | rs2028028 | 112106228 | A | 0.32 | 0.28 | 5.75e-05 |  |  |  |  |  |
| 7 | rs6970266 | 112126878 | C | 0.32 | 0.28 | 5.45e-05 |  |  |  |  |  |
| 7 | rs10216223 | 112140640 | T | 0.32 | 0.28 | 6.44e-05 |  |  |  |  |  |
| 7 | rs1615468 | 112163233 | C | 0.32 | 0.28 | 5.4e-05 | 0.26 | 0.29 | 5.23e-03 | 0.83 (0.73, 0.95) | 0.09 |
| 8 | rs6981011 | 22353566 | G | 1.95e-03 | 0.00e+00 | 3.36e-05 | 1.74e-03 | 7.63e-04 | 0.26 | 2.12 (0.41, 10.96) | 5.52e-05 |
| 8 | rs7000566 | 22448785 | A | 1.82e-03 | 0.00e+00 | 5.61e-05 | 1.74e-03 | 7.62e-04 | 0.07 | 2.5 (0.48, 13.01) | 1.54e-05 |
| 8 | rs1497035 | 61014695 | T | 0.35 | 0.39 | 9.01e-05 |  |  |  |  |  |
| 8 | rs11784289 | 61018492 | T | 0.35 | 0.39 | 9.83e-05 |  |  |  |  |  |
| 8 | rs11784406 | 61018884 | T | 0.35 | 0.39 | 9.81e-05 |  |  |  |  |  |
| 8 | rs12681623 | 120973037 | C | 0.4 | 0.44 | 8.46e-05 |  |  |  |  |  |
| 8 | rs13260933 | 120976431 | G | 0.4 | 0.44 | 8.01e-05 |  |  |  |  |  |
| 8 | rs6987580 | 120979561 | A | 0.4 | 0.44 | 3.57e-05 |  |  |  |  |  |
| 8 | rs7462302 | 120986952 | G | 0.4 | 0.44 | 3.44e-05 | 0.4 | 0.44 | 0.03 | 0.88 (0.78, 0.99) | 3.93e-06 |
| 8 | rs9918814 | 121020783 | G | 0.3 | 0.34 | 4.72e-05 | 0.3 | 0.34 | 0.02 | 0.86 (0.76, 0.97) | 2.72e-06 |
| 9 | rs6478389 | 120750495 | C | 0.02 | 0.01 | 5.96e-05 | 0.01 | 0.01 | 0.89 | 0.98 (0.57, 1.67) | 1.37e-03 |
| 9 | rs538324 | 137691723 | C | 0.41 | 0.37 | 9.0e-05 | 0.4 | 0.4 | 0.86 | 1.01 (0.90, 1.14) | 9.65e-04 |
| 10 | rs911547 | 105629411 | C | 0.12 | 0.15 | 2.66e-05 | 0.13 | 0.15 | 0.03 | 0.83 (0.70, 0.99) | 3.22e-06 |
| 10 | rs11191841 | 105629601 | T | 0.45 | 0.5 | 8.64e-07 |  |  |  |  |  |
| 10 | rs7100920 | 105630968 | C | 0.45 | 0.5 | 2.27e-07 |  |  |  |  |  |
| 10 | rs9325507 | 105635612 | C | 0.45 | 0.5 | 2.0e-07 | 0.45 | 0.49 | 4.72e-03 | 0.85 (0.75, 0.95) | 4.19e-09 |
| 10 | rs10883942 | 105641376 | T | 0.45 | 0.51 | 2.37e-07 |  |  |  |  |  |
| 10 | rs4918069 | 105644381 | G | 0.31 | 0.26 | 5.71e-06 |  |  |  |  |  |
| 10 | rs12765878 | 105659612 | T | 0.45 | 0.51 | 2.35e-07 |  |  |  |  |  |
| 10 | rs1265165 | 105661517 | T | 0.07 | 0.1 | 2.74e-05 |  |  |  |  |  |
| 10 | rs2756116 | 105661794 | T | 0.08 | 0.1 | 7.47e-05 |  |  |  |  |  |
| 10 | rs1265164 | 105664844 | T | 0.12 | 0.16 | 1.25e-05 | 0.12 | 0.14 | 0.06 | 0.84 (0.71, 1.00) | 3.01e-06 |
| 10 | rs7902587 | 105684291 | T | 0.08 | 0.1 | 1.54e-05 | 0.09 | 0.1 | 0.19 | 0.87 (0.71, 1.07) | 1.82e-05 |
| 10 | rs7099625 | 105693788 | T | 0.31 | 0.27 | 2.36e-05 | 0.31 | 0.29 | 0.26 | 1.07 (0.95, 1.22) | 4.08e-05 |
| 10 | rs1342212 | 105698803 | G | 0.39 | 0.35 | 4.74e-06 | 0.36 | 0.35 | 0.45 | 1.05 (0.93, 1.19) | 2.95e-05 |
| 10 | rs7079220 | 105700137 | C | 0.33 | 0.28 | 1.41e-05 |  |  |  |  |  |
| 11 | rs11245920 | 1052519 | C | 0.05 | 0.07 | 3.29e-05 | 0.06 | 0.06 | 0.93 | 1.01 (0.79, 1.29) | 8.35e-04 |
| 11 | rs10794281 | 1053149 | C | 0.48 | 0.43 | 3.39e-07 |  |  |  |  |  |
| 11 | rs10751638 | 1053247 | G | 0.53 | 0.49 | 1.85e-05 |  |  |  |  |  |
| 11 | rs10902075 | 1055791 | T | 0.49 | 0.43 | 1.8e-07 |  |  |  |  |  |
| 11 | rs2856082 | 1061562 | G | 0.1 | 0.13 | 2.52e-06 | 0.11 | 0.12 | 0.31 | 0.91 (0.76, 1.09) | 9.21e-06 |
| 11 | rs11245936 | 1074362 | A | 0.06 | 0.08 | 7.61e-06 | 0.07 | 0.09 | 0.03 | 0.78 (0.63, 0.98) | 9.2e-07 |
| 11 | rs11245954 | 1091078 | G | 0.06 | 0.08 | 2.65e-06 |  |  |  |  |  |
| 11 | rs7927765 | 1094626 | T | 0.06 | 0.09 | 9.85e-07 | 0.08 | 0.08 | 0.48 | 0.93 (0.75, 1.15) | 1.05e-05 |
| 11 | rs3924453 | 1095806 | T | 0.24 | 0.28 | 1.42e-05 |  |  |  |  |  |
| 11 | rs7949616 | 1097989 | G | 0.06 | 0.09 | 1.58e-06 |  |  |  |  |  |
| 11 | rs5743890 | 1282405 | C | 0.12 | 0.15 | 3.11e-06 |  |  |  |  |  |
| 11 | rs1033198 | 1303378 | T | 0.41 | 0.47 | 9.96e-08 |  |  |  |  |  |
| 11 | rs7118481 | 1304914 | G | 0.41 | 0.47 | 1.09e-07 |  |  |  |  |  |
| 11 | rs10832253 | 1321818 | C | 0.27 | 0.31 | 9.87e-07 |  |  |  |  |  |
| 11 | rs11023369 | 1326427 | G | 0.28 | 0.32 | 2.75e-06 |  |  |  |  |  |
| 11 | rs6578295 | 1346787 | C | 0.28 | 0.33 | 2.93e-07 |  |  |  |  |  |
| 11 | rs4247481 | 1359205 | A | 0.4 | 0.45 | 1.06e-06 |  |  |  |  |  |
| 11 | rs7394830 | 1365468 | G | 0.34 | 0.3 | 2.94e-05 |  |  |  |  |  |
| 11 | rs10833086 | 1370392 | G | 0.35 | 0.31 | 1.64e-05 | 0.36 | 0.31 | 2.37e-03 | 1.21 (1.07, 1.37) | 1.34e-07 |
| 11 | rs12801744 | 1449708 | T | 0.11 | 0.08 | 1.53e-05 | 0.09 | 0.08 | 0.07 | 1.20 (0.98, 1.47) | 4.94e-06 |
| 12 | rs12580363 | 44440217 | T | 2.90e-03 | 0.01 | 6.55e-05 |  |  |  |  |  |
| 12 | rs11833585 | 77918510 | G | 0.13 | 0.1 | 5.93e-05 | 0.1 | 0.11 | 0.47 | 0.93 (0.78, 1.12) | 4.22e-03 |
| 13 | rs1408180 | 38896559 | T | 3.70e-03 | 3.02e-04 | 1.19e-05 | 2.89e-03 | 2.28e-03 | 0.57 | 1.44 (0.47, 4.45) | 9.33e-05 |
| 13 | rs1323939 | 38897420 | A | 3.70e-03 | 3.01e-04 | 1.18e-05 | 2.90e-03 | 2.29e-03 | 0.57 | 1.44 (0.47, 4.44) | 9.28e-05 |
| 13 | rs7323436 | 40248979 | G | 0.33 | 0.28 | 1.7e-05 | 0.31 | 0.31 | 0.46 | 1.05 (0.92, 1.19) | 8.28e-05 |
| 13 | rs3783164 | 40278363 | C | 0.32 | 0.28 | 5.12e-05 |  |  |  |  |  |
| 13 | rs7999955 | 96241462 | A | 2.10e-03 | 0.00e+00 | 3.0e-05 | 5.79e-04 | 0.00e+00 | 0.31 | 2502000000.0 (0.0, inf) | NA |
| 14 | rs1953852 | 44161413 | A | 0.41 | 0.45 | 2.95e-05 | 0.45 | 0.43 | 0.11 | 1.10 (0.98, 1.23) | 0.01 |
| 14 | rs4326955 | 44180078 | C | 0.42 | 0.46 | 6.96e-05 |  |  |  |  |  |
| 14 | rs7142648 | 44193052 | A | 0.42 | 0.46 | 6.26e-05 |  |  |  |  |  |
| 14 | rs10484172 | 44213211 | G | 0.42 | 0.46 | 5.18e-05 |  |  |  |  |  |
| 14 | rs2039790 | 44221300 | G | 0.42 | 0.46 | 4.75e-05 |  |  |  |  |  |
| 14 | rs1953856 | 44226867 | G | 0.46 | 0.51 | 2.48e-05 |  |  |  |  |  |
| 14 | rs811722 | 44251087 | A | 0.54 | 0.49 | 2.23e-05 | 0.51 | 0.49 | 0.27 | 1.07 (0.95, 1.20) | 4.75e-03 |
| 14 | rs807326 | 44255590 | C | 0.52 | 0.48 | 5.01e-05 |  |  |  |  |  |
| 14 | rs1869099 | 46075559 | C | 0.28 | 0.32 | 5.79e-05 | 0.3 | 0.32 | 0.04 | 0.88 (0.77, 1.00) | 8.28e-06 |
| 14 | rs6572793 | 51363999 | G | 0.03 | 0.02 | 3.53e-05 |  |  |  |  |  |
| 14 | rs6572795 | 51364147 | G | 0.04 | 0.02 | 2.43e-05 | 0.03 | 0.02 | 0.04 | 1.44 (1.02, 2.04) | 3.1e-06 |
| 14 | rs2983088 | 51371842 | G | 0.03 | 0.02 | 9.89e-05 |  |  |  |  |  |
| 14 | rs4906405 | 103568225 | G | 2.23e-03 | 1.51e-04 | 5.22e-05 | 1.77e-03 | 5.17e-04 | 0.03 | 5.13 (0.83, 31.69) | 5.16e-06 |
| 14 | rs10150865 | 103570180 | A | 2.23e-03 | 1.51e-04 | 5.22e-05 | 1.74e-03 | 5.07e-04 | 0.03 | 5.11 (0.83, 31.55) | 5.16e-06 |
| 15 | rs2289329 | 38492451 | C | 0.44 | 0.39 | 1.45e-06 |  |  |  |  |  |
| 15 | rs2289326 | 38534040 | T | 0.44 | 0.49 | 2.95e-05 | 0.46 | 0.47 | 0.31 | 0.94 (0.84, 1.06) | 6.52e-05 |
| 15 | rs3803357 | 38538847 | C | 0.47 | 0.51 | 7.39e-05 |  |  |  |  |  |
| 15 | rs11638110 | 83623888 | G | 0.16 | 0.19 | 8.41e-05 |  |  |  |  |  |
| 15 | rs12438724 | 83626297 | T | 0.43 | 0.38 | 1.28e-07 | 0.4 | 0.39 | 0.53 | 1.04 (0.92, 1.17) | 2.92e-06 |
| 15 | rs2002642 | 83628748 | C | 0.16 | 0.19 | 7.25e-05 |  |  |  |  |  |
| 15 | rs12917495 | 83630565 | A | 0.16 | 0.19 | 5.74e-05 | 0.19 | 0.2 | 0.55 | 0.96 (0.82, 1.11) | 2.83e-04 |
| 15 | rs8035975 | 83639394 | G | 0.43 | 0.38 | 2.84e-07 |  |  |  |  |  |
| 15 | rs10520583 | 83639906 | A | 0.43 | 0.38 | 2.16e-07 |  |  |  |  |  |
| 15 | rs2048439 | 83643085 | C | 0.43 | 0.38 | 1.82e-07 |  |  |  |  |  |
| 15 | rs7180588 | 83652745 | A | 0.45 | 0.39 | 4.76e-07 |  |  |  |  |  |
| 15 | rs7177311 | 83657094 | G | 0.45 | 0.39 | 4.9e-07 |  |  |  |  |  |
| 15 | rs12591489 | 83671371 | G | 0.44 | 0.39 | 1.1e-06 |  |  |  |  |  |
| 15 | rs16949988 | 83690999 | T | 0.43 | 0.38 | 2.76e-07 |  |  |  |  |  |
| 15 | rs11855785 | 83691589 | G | 0.39 | 0.35 | 3.93e-07 |  |  |  |  |  |
| 15 | rs8029576 | 83695801 | T | 0.43 | 0.38 | 2.0e-07 |  |  |  |  |  |
| 15 | rs7179284 | 83696566 | C | 0.43 | 0.38 | 3.05e-07 |  |  |  |  |  |
| 15 | rs17631906 | 83886124 | G | 0.22 | 0.18 | 2.27e-05 | 0.2 | 0.19 | 0.62 | 1.04 (0.90, 1.20) | 1.77e-04 |
| 15 | rs16941157 | 83887737 | G | 0.32 | 0.28 | 6.8e-06 | 0.31 | 0.29 | 0.17 | 1.09 (0.96, 1.24) | 7.96e-06 |
| 15 | rs16941432 | 83904180 | G | 0.41 | 0.36 | 6.03e-08 | 0.4 | 0.37 | 0.08 | 1.11 (0.99, 1.25) | 5.84e-08 |
| 15 | rs7177107 | 83924368 | A | 0.26 | 0.22 | 1.88e-05 |  |  |  |  |  |
| 15 | rs10520594 | 83929645 | A | 0.26 | 0.22 | 5.07e-05 |  |  |  |  |  |
| 15 | rs10520595 | 83929721 | T | 0.35 | 0.3 | 1.95e-07 | 0.33 | 0.31 | 0.25 | 1.07 (0.95, 1.21) | 8.98e-07 |
| 15 | rs8034676 | 83960266 | G | 0.4 | 0.35 | 1.41e-06 |  |  |  |  |  |
| 15 | rs7181796 | 84034013 | C | 0.26 | 0.22 | 2.54e-05 |  |  |  |  |  |
| 15 | rs7179917 | 84066648 | A | 0.26 | 0.22 | 1.02e-05 | 0.24 | 0.22 | 0.53 | 1.04 (0.91, 1.19) | 7.45e-05 |
| 15 | rs17638180 | 84079399 | C | 0.26 | 0.22 | 1.4e-05 |  |  |  |  |  |
| 15 | rs17576534 | 84086744 | T | 0.26 | 0.22 | 1.55e-05 |  |  |  |  |  |
| 15 | rs7173923 | 84093475 | A | 0.28 | 0.24 | 4.9e-05 |  |  |  |  |  |
| 15 | rs2554 | 84103647 | C | 0.28 | 0.24 | 3.02e-05 |  |  |  |  |  |
| 16 | rs4984902 | 595263 | G | 0.18 | 0.21 | 3.52e-05 | 0.19 | 0.22 | 0.05 | 0.86 (0.74, 1.00) | 6.26e-06 |
| 16 | rs2269556 | 615216 | A | 0.19 | 0.22 | 9.46e-05 |  |  |  |  |  |
| 16 | rs2076142 | 640971 | T | 0.2 | 0.23 | 3.82e-05 |  |  |  |  |  |
| 16 | rs11642546 | 641657 | T | 0.2 | 0.24 | 6.07e-05 | 0.2 | 0.23 | 0.01 | 0.83 (0.72, 0.96) | 2.7e-06 |
| 16 | rs34775748 | 646669 | A | 0.2 | 0.23 | 5.07e-05 |  |  |  |  |  |
| 16 | rs4984907 | 649631 | C | 0.2 | 0.23 | 4.83e-05 |  |  |  |  |  |
| 16 | rs11640115 | 675795 | G | 0.2 | 0.23 | 9.36e-05 |  |  |  |  |  |
| 16 | rs763053 | 675922 | G | 0.2 | 0.23 | 2.41e-05 | 0.21 | 0.23 | 0.12 | 0.89 (0.78, 1.03) | 1.4e-05 |
| 16 | rs4984916 | 689058 | A | 0.19 | 0.23 | 5.12e-05 |  |  |  |  |  |
| 17 | rs12947718 | 40848884 | A | 0.15 | 0.18 | 1.18e-06 |  |  |  |  |  |
| 17 | rs12942666 | 40855622 | G | 0.15 | 0.19 | 1.1e-06 |  |  |  |  |  |
| 17 | rs34018943 | 40864086 | G | 0.15 | 0.18 | 9.02e-07 |  |  |  |  |  |
| 17 | rs2521840 | 40892951 | G | 0.32 | 0.28 | 4.91e-05 |  |  |  |  |  |
| 17 | rs17631676 | 40905309 | G | 0.15 | 0.19 | 7.91e-07 |  |  |  |  |  |
| 17 | rs4523963 | 40926009 | T | 0.28 | 0.24 | 3.44e-05 | 0.27 | 0.26 | 0.35 | 1.06 (0.93, 1.21) | 8.89e-05 |
| 17 | rs1724409 | 41096348 | G | 0.21 | 0.26 | 5.31e-07 |  |  |  |  |  |
| 17 | rs4510068 | 41540611 | T | 0.35 | 0.4 | 6.48e-08 | 0.21 | 0.23 | 0.28 | 0.94 (0.83, 1.06) | 4.82e-07 |
| 17 | rs199454 | 42155294 | G | 0.19 | 0.24 | 6.06e-08 |  |  |  |  |  |
| 17 | rs199449 | 42164086 | A | 0.19 | 0.24 | 6.24e-08 |  |  |  |  |  |
| 17 | rs199444 | 42173441 | C | 0.19 | 0.24 | 7.29e-08 |  |  |  |  |  |
| 17 | rs199442 | 42175290 | A | 0.2 | 0.24 | 2.68e-07 |  |  |  |  |  |
| 17 | rs199536 | 42175593 | T | 0.19 | 0.24 | 1.16e-07 |  |  |  |  |  |
| 17 | rs199530 | 42191820 | G | 0.2 | 0.25 | 2.74e-07 |  |  |  |  |  |
| 17 | rs199529 | 42192384 | C | 0.18 | 0.22 | 5.4e-08 |  |  |  |  |  |
| 17 | rs199520 | 42209035 | G | 0.2 | 0.25 | 9.42e-08 |  |  |  |  |  |
| 17 | rs199501 | 42217772 | A | 0.2 | 0.24 | 3.15e-07 |  |  |  |  |  |
| 18 | rs6508330 | 20633962 | C | 0.03 | 0.02 | 9.34e-06 | 0.02 | 0.03 | 0.22 | 0.78 (0.53, 1.16) | 3.62e-03 |
| 18 | rs11083093 | 20647281 | A | 0.03 | 0.01 | 5.55e-06 | 0.02 | 0.03 | 0.11 | 0.72 (0.48, 1.08) | 5.53e-03 |
| 19 | rs12462642 | 4666016 | A | 0.36 | 0.32 | 1.26e-05 |  |  |  |  |  |
| 19 | rs708686 | 5791619 | T | 0.35 | 0.31 | 8.42e-05 | 0.3 | 0.29 | 0.26 | 1.07 (0.95, 1.22) | 1.12e-04 |
| 19 | rs35791962 | 54875261 | T | 0.04 | 0.06 | 3.61e-06 | 0.05 | 0.04 | 0.63 | 1.08 (0.82, 1.42) | 4.64e-04 |
| 19 | rs10415880 | 54880968 | A | 0.29 | 0.33 | 2.77e-05 | 0.31 | 0.34 | 0.14 | 0.91 (0.80, 1.03) | 1.83e-05 |
| 19 | rs10415869 | 54895397 | A | 0.16 | 0.19 | 9.13e-05 | 0.16 | 0.17 | 0.3 | 0.92 (0.78, 1.08) | 1.49e-04 |
| 19 | rs10416103 | 54895496 | C | 0.16 | 0.19 | 9.32e-05 |  |  |  |  |  |
| 19 | rs306489 | 61174242 | T | 0.46 | 0.42 | 2.61e-05 | 0.45 | 0.44 | 0.65 | 1.03 (0.91, 1.16) | 2.21e-04 |
| 19 | rs306483 | 61179242 | A | 0.46 | 0.42 | 2.99e-05 |  |  |  |  |  |
| 19 | rs306473 | 61183480 | G | 0.46 | 0.42 | 5.94e-05 |  |  |  |  |  |
| 20 | rs6028053 | 59168898 | A | 0.23 | 0.19 | 2.89e-05 |  |  |  |  |  |
| 20 | rs6015928 | 59169114 | C | 0.23 | 0.2 | 1.47e-05 | 0.22 | 0.21 | 0.52 | 1.05 (0.91, 1.21) | 9.27e-05 |
| 21 | rs2828651 | 24251826 | A | 0.27 | 0.24 | 4.6e-05 | 0.28 | 0.26 | 0.11 | 1.11 (0.98, 1.26) | 2.23e-05 |
| 23 | rs7882581 | 79012333 | A | 0.13 | 0.1 | 8.81e-05 |  |  |  |  |  |
| 23 | rs7053926 | 79015548 | A | 0.13 | 0.1 | 8.34e-05 |  |  |  |  |  |
| 23 | rs6418205 | 79018612 | A | 0.13 | 0.1 | 7.75e-05 | 0.11 | 0.1 | 0.73 | 1.04 (0.83, 1.31) | 6.18e-04 |
| 23 | rs6418206 | 79018637 | T | 0.13 | 0.1 | 7.81e-05 |  |  |  |  |  |
| 23 | rs6523474 | 79018912 | G | 0.13 | 0.1 | 7.78e-05 |  |  |  |  |  |
| 23 | rs6523497 | 79034257 | G | 0.15 | 0.11 | 7.93e-06 | 0.14 | 0.13 | 0.25 | 1.13 (0.92, 1.39) | 1.66e-05 |
| 23 | rs6621129 | 79053933 | T | 0.16 | 0.12 | 1.06e-05 | 0.13 | 0.12 | 0.41 | 1.09 (0.88, 1.35) | 4.66e-05 |

**^a^Minor allele frequency among GWAS cases based on imputed counts**

**^b^Minor allele frequency among GWAS controls based on imputed counts**

**^c^P-value from imputation analysis under additive model using discovery GWAS samples**

**^d^Only SNPs sent for replication genotyping have these results; none of the Chromosome 17 SNPs were sent for replication genotyping since all in very high LD with known SNP from original GWAS (Fingerlin et al. 2013).**

**^e^Minor allele frequency among replication cases**

**^f^Minor allele frequency among replication controls**

**^g^P-value from additive model among replication cases and controls**

**^h^Odds ratio and 95% CI among replication cases and controls**

**^i^P-value from meta-analysis of discovery imputation and replication.**

**S3: GWAS-Significant SNPs after Meta-Analysis in All Regions (Most Significant in Region in Bold Type)**

| **Locus** | **Position** | **SNP** | **Minor allele** | **P Meta^a^** | **P Joint^b^** | **OR (95% CI) Joint^c^** |
| --- | --- | --- | --- | --- | --- | --- |
| 3q26 | 170947153 | rs1881984 | C | 4.53e-08 | 4.1e-04 | 1.19 (1.08,1.31) |
|  | 170947636 | rs13097028 | T | 5.61e-09 | 6.74e-05 | 1.20 (1.1,1.32) |
|  | **170963965** | **rs12696304** | **G** | **8.22e-14** | **1.88e-08** | **1.32 (1.20,1.45)** |
|  | 170974795 | rs10936599 | T | 2.51e-11 | 1.54e-08 | 1.35 (1.22,1.49) |
|  | 170992346 | rs1997392 | A | 3.2e-12 | 1.31e-07 | 1.31 (1.19,1.45) |
|  | 171001149 | rs6793295 | C | 8.33e-13 | 4.02e-08 | 1.32 (1.20,1.47) |
|  | 171035075 | rs7647824 | G | 1.43e-08 | 1.14e-06 | 1.28 (1.16,1.42) |
| 4q22 | 90019915 | rs1379932 | T | 8.82e-12 | 4.19e-07 | 1.31 (1.18,1.45) |
|  | 90030218 | rs2609255 | G | 2.2e-11 | 1.7e-06 | 1.30 (1.17,1.45) |
|  | **90054508** | **rs2609261** | **T** | **2.42e-12** | **2.01e-07** | **1.32 (1.19,1.47)** |
|  | 90055842 | rs2609260 | G | 4.38e-10 | 8.06e-07 | 1.32 (1.18,1.48) |
| 5p15 | **1332790** | **rs10069690** | **T** | **1.19e-15** | **4.14e-10** | **0.72 (0.64,0.79)** |
|  | 1339516 | rs2736100 | G | 1.71e-19 | 3.97e-09 | 0.76 (0.7,0.83) |
|  | 1340194 | rs2853677 | C | 3.59e-12 | 1.03e-11 | 0.73 (0.67,0.8) |
|  | 1341547 | rs2853676 | A | 3.31e-08 | 2.87e-04 | 0.82 (0.74,0.91) |
| 6p24 | 7502047 | rs10484325 | C | 4.67e-08 | 3.84e-04 | 1.25 (1.10,1.41) |
|  | 7502247 | rs3799525 | G | 2.3e-08 | 1.82e-04 | 0.82 (0.74,0.91) |
|  | 7503317 | rs10484326 | C | 5.45e-09 | 7.64e-04 | 0.83 (0.75,0.93) |
|  | **7508231** | **rs2076295** | **G** | **1.08e-19** | **3.69e-08** | **1.28 (1.18,1.40)** |
| 6p21 | 31948604 | rs614549 | C | 2.09e-08 | 1.16e-06 | 0.80 (0.73,0.87) |
|  | **31972526** | **rs7887** | **A** | **3.7e-09** | **1.28e-07** | **0.78 (0.71,0.86)** |
|  | 31990003 | rs2844452 | C | 4.55e-08 | 1.63e-06 | 0.81 (0.74,0.88) |
|  | 32002605 | rs3020644 | G | 2.68e-08 | 5.38e-07 | 0.80 (0.73,0.87) |
|  | 32036670 | rs2280774 | T | 3.89e-08 | 8.54e-06 | 0.81 (0.74,0.89) |
|  | 32474995 | rs3117116 | C | 2.65e-08 | 4.72e-04 | 1.25 (1.10,1.41) |
| 7q22 | **99431282** | **rs4727443** | **A** | **1.17e-08** | **4.1e-07** | **1.27 (1.16,1.39)** |
| 10q24 | 105629504 | rs10748858 | G | 2.65e-08 | 2.0e-03 | 0.86 (0.79,0.95) |
|  | 105633124 | rs2067832 | C | 3.67e-08 | 3.15e-04 | 0.85 (0.77,0.93) |
|  | **105635612** | **rs9325507** | **C** | **4.19e-09** | **6.08e-04** | **0.86 (0.79,0.94)** |
|  | 105662832 | rs11191865 | G | 2.44e-08 | 3.2e-04 | 0.85 (0.77,0.93) |
| 11p15 | 1053767 | rs2301160 | C | 1.24e-08 | 2.49e-05 | 1.22 (1.11,1.33) |
|  | 1058900 | rs7942850 | G | 1.71e-12 | 3.92e-05 | 1.22 (1.11,1.33) |
|  | **1083945** | **rs7934606** | **A** | **6.87e-34** | **1.14e-28** | **1.71 (1.55,1.88)** |
|  | 1086494 | rs6421972 | A | 1.44e-33 | 1.31e-28 | 1.71 (1.55,1.87) |
|  | 1091649 | rs7480563 | G | 2.95e-17 | 2.08e-08 | 0.77 (0.70,0.84) |
|  | 1095976 | rs4077759 | C | 2.14e-11 | 3.53e-06 | 0.80 (0.72,0.88) |
|  | 1197581 | rs2672794 | T | 1.77e-17 | 6.97e-19 | 0.65 (0.59,0.72) |
|  | 1199266 | rs868903 | T | 9.18e-26 | 2.4e-15 | 0.69 (0.63,0.75) |
|  | 1207064 | rs2075859 | T | 7.68e-16 | 2.26e-09 | 0.75 (0.68,0.83) |
|  | 1218216 | rs2735733 | T | 2.41e-10 | 4.82e-06 | 0.81 (0.74,0.89) |
|  | 1229434 | rs4963059 | T | 7.6e-13 | 7.78e-08 | 0.76 (0.69,0.84) |
|  | 1237710 | rs2857476 | C | 4.68e-08 | 6.0e-08 | 0.78 (0.71,0.85) |
|  | 1240803 | rs12417955 | G | 4.46e-08 | 3.69e-08 | 0.77 (0.70,0.85) |
|  | 1253225 | rs3168046 | T | 3.81e-18 | 4.44e-14 | 1.41 (1.29,1.54) |
|  | 1256982 | rs3829223 | C | 9.07e-10 | 6.21e-10 | 0.75 (0.68,0.82) |
|  | 1266532 | rs3750920 | T | 1.03e-14 | 2.27e-12 | 1.37 (1.26,1.50) |
|  | 1313639 | rs2334659 | T | 1.22e-12 | 9.75e-07 | 0.72 (0.63,0.82) |
|  | 1331032 | rs7122936 | C | 1.02e-11 | 3.08e-08 | 0.76 (0.69,0.84) |
|  | 1586753 | rs12792005 | A | 7.04e-13 | 1.0e-06* | 1.82 (1.48,2.24) |
|  | 1599178 | rs35678986 | G | 3.56e-17 | 1.0e-06* | 2.22 (1.71,2.87) |
| 13q34 | 112584628 | rs1278769 | A | 6.72e-09 | 2.91e-05 | 0.79 (0.71,0.88) |
| 15q14 | 38439130 | rs1007177 | T | 1.26e-09 | 5.85e-04 | 0.84 (0.76,0.93) |
|  | 38446262 | rs1992272 | A | 2.16e-09 | 2.44e-03 | 0.86 (0.78,0.95) |
|  | 38487314 | rs10518693 | T | 2.32e-08 | 1.41e-03 | 1.16 (1.06,1.27) |
|  | 38504594 | rs2034650 | C | 9.76e-12 | 1.18e-05 | 0.82 (0.75,0.90) |
| 17q21 | 41074926 | rs393152 | G | 3.5e-09 | 6.13e-08 | 0.73 (0.65,0.82) |
|  | 41279910 | rs12373139 | A | 2.68e-13 | 5.09e-09 | 0.71 (0.63,0.8) |
|  | 41281077 | rs17690703 | T | 1.04e-08 | 8.53e-07 | 0.76 (0.68,0.85) |
|  | 41347100 | rs17563986 | G | 1.27e-13 | 3.44e-09 | 0.71 (0.63,0.79) |
|  | **41412603** | **rs1981997** | **A** | **8.87e-14** | **4.87e-09** | 0.71 (0.63,0.79) |
|  | 41436901 | rs8070723 | G | 1.61e-12 | 5.22e-09 | 0.71 (0.63,0.8) |
|  | 41544850 | rs7225002 | G | 3.04e-09 | 1.39e-05 | 0.81 (0.74,0.89) |
|  | 41602941 | rs2532274 | C | 2.43e-12 | 2.26e-09 | 0.70 (0.63,0.79) |
|  | 41605885 | rs2532269 | G | 1.61e-13 | 7.62e-10 | 0.70 (0.62,0.78) |
|  | 41648797 | rs2668692 | T | 3.12e-13 | 2.3e-09 | 0.70 (0.63,0.79) |
|  | 42145386 | rs169201 | G | 1.16e-11 | 5.26e-08 | 0.72 (0.64,0.81) |
|  | 42184098 | rs199533 | T | 1.99e-11 | 1.11e-07 | 0.72 (0.64,0.81) |
|  | 42214305 | rs415430 | G | 1.48e-10 | 4.43e-08 | 0.72 (0.64,0.81) |
| 19p13 | **4668672** | **rs12610495** | **G** | **1.68e-12** | **1.54e-07** | **1.30 (1.18,1.43)** |
|  | 4670443 | rs2109069 | A | 2.42e-11 | 3.98e-07 | 1.28 (1.16,1.41) |

**^a^P-value from meta-analysis of discovery imputation and replication.**

**^b^P-value from joint analysis comparing subset of GWAS cases (1498) and replication cases (878) to replication controls (2017) based on observed genotypes; GWAS cases were genotyped at same time as replication cases and controls.**

**^c^Odds ratio and 95% CI for joint analysis.**

**S4: Table of 70 SNPs (46 original, 24 from imputation) Adjusted for Top SNP in Region if More than One SNP in Region. Top SNP Defined in Bold Type in Table S3^a^. All Cases (Discovery and GWAS) Compared to Replication Controls.**

| **Locus** | **SNP** | **Position** | **P Joint**  **Unadjusted for Best SNP^b^** | **Best SNP**  **in Region^b^** | **P Joint**  **Adjusted for Best SNP^b^** |
| --- | --- | --- | --- | --- | --- |
| 3q26 | rs1881984 | 170947153 | 4.1e-04 | rs12696304 | 0.36 |
|  | rs13097028 | 170947636 | 6.74e-05 | rs12696304 | 0.51 |
|  | rs12696304 | 170963965 | 1.88e-08 |  |  |
|  | rs10936599 | 170974795 | 1.54e-08 | rs12696304 | 0.14 |
|  | rs1997392 | 170992346 | 1.31e-07 | rs12696304 | 0.84 |
|  | rs6793295 | 171001149 | 4.02e-08 | rs12696304 | 0.6 |
|  | rs7647824 | 171035075 | 1.14e-06 | rs12696304 | 0.29 |
| 4q22 | rs1379932 | 90019915 | 4.19e-07 | rs2609261 | 1.0 |
|  | rs2609255 | 90030218 | 1.7e-06 | rs2609261 | 0.17 |
|  | rs2609261 | 90054508 | 2.01e-07 |  |  |
|  | rs2609260 | 90055842 | 8.06e-07 | rs2609261 | 0.32 |
| 5p15 | rs10069690 | 1332790 | 4.14e-10 | rs2736100 | 2.07e-04 |
|  | rs2736100 | 1339516 | 3.97e-09 |  |  |
|  | rs2853677 | 1340194 | 1.03e-11 | rs2736100 | 3.28e-04 |
|  | rs2853676 | 1341547 | 2.87e-04 | rs2736100 | 0.16 |
| 6p24 | rs10484325 | 7502047 | 3.84e-04 | rs2076295 | 0.18 |
|  | rs3799525 | 7502247 | 1.82e-04 | rs2076295 | 0.47 |
|  | rs10484326 | 7503317 | 7.64e-04 | rs2076295 | 0.52 |
|  | rs2076295 | 7508231 | 3.69e-08 |  |  |
| 6p21 | rs614549 | 31948604 | 1.16e-06 | rs7887 | 0.26 |
|  | rs7887 | 31972526 | 1.28e-07 |  |  |
|  | rs2844452 | 31990003 | 1.63e-06 | rs7887 | 0.26 |
|  | rs3020644 | 32002605 | 5.38e-07 | rs7887 | 0.28 |
|  | rs2280774 | 32036670 | 8.54e-06 | rs7887 | 0.83 |
|  | rs3117116 | 32474995 | 4.72e-04 | rs7887 | 0.02 |
| 7q22 | rs4727443 | 99431282 | 4.1e-07 |  |  |
| 10q24 | rs10748858 | 105629504 | 2.0e-03 | rs9325507 | 0.53 |
|  | rs2067832 | 105633124 | 3.15e-04 | rs9325507 | NA |
|  | rs9325507 | 105635612 | 6.08e-04 |  |  |
|  | rs11191865 | 105662832 | 3.2e-04 | rs9325507 | 0.56 |
| 11p15 | rs2301160 | 1053767 | 2.49e-05 | rs35705950 | 0.39 |
|  | rs7942850 | 1058900 | 3.92e-05 | rs35705950 | 0.78 |
|  | rs7934606 | 1083945 | 1.14e-28 | rs35705950 | 0.19 |
|  | rs6421972 | 1086494 | 1.31e-28 | rs35705950 | 0.20 |
|  | rs7480563 | 1091649 | 2.08e-08 | rs35705950 | 0.32 |
|  | rs4077759 | 1095976 | 3.53e-06 | rs35705950 | 0.43 |
|  | rs2672794 | 1197581 | 6.97e-19 | rs35705950 | 0.65 |
|  | rs35705950 | 1197797 | 5.06e-121 |  |  |
|  | rs868903 | 1199266 | 2.4e-15 | rs35705950 | 0.99 |
|  | rs2075859 | 1207064 | 2.26e-09 | rs35705950 | 0.98 |
|  | rs2735733 | 1218216 | 4.82e-06 | rs35705950 | 0.26 |
|  | rs4963059 | 1229434 | 7.78e-08 | rs35705950 | 0.98 |
|  | rs2857476 | 1237710 | 6.0e-08 | rs35705950 | 0.25 |
|  | rs12417955 | 1240803 | 3.69e-08 | rs35705950 | 0.20 |
|  | rs3168046 | 1253225 | 4.44e-14 | rs35705950 | 0.77 |
|  | rs3829223 | 1256982 | 6.21e-10 | rs35705950 | 0.67 |
|  | rs3750920 | 1266532 | 2.27e-12 | rs35705950 | 0.23 |
|  | rs2334659 | 1313639 | 9.75e-07 | rs35705950 | 0.38 |
|  | rs7122936 | 1331032 | 3.08e-08 | rs35705950 | 1.00 |
|  | rs12792005 | 1586753 | 1.0e-06 | rs35705950 | 0.40 |
|  | rs35678986 | 1599178 | 1.0e-06 | rs35705950 | 0.43 |
| 13q34 | rs1278769 | 112584628 | 2.91e-05 |  |  |
| 15q14 | rs1007177 | 38439130 | 5.85e-04 | rs2034650 | 0.72 |
|  | rs1992272 | 38446262 | 2.44e-03 | rs2034650 | 0.95 |
|  | rs10518693 | 38487314 | 1.41e-03 | rs2034650 | 0.94 |
|  | rs2034650 | 38504594 | 1.18e-05 |  |  |
| 17q21 | rs393152 | 41074926 | 6.13e-08 | rs1981997 | 0.34 |
|  | rs12373139 | 41279910 | 5.09e-09 | rs1981997 | NA |
|  | rs17690703 | 41281077 | 8.53e-07 | rs1981997 | 0.56 |
|  | rs17563986 | 41347100 | 3.44e-09 | rs1981997 | NA |
|  | rs1981997 | 41412603 | 4.87e-09 |  |  |
|  | rs8070723 | 41436901 | 5.22e-09 | rs1981997 | NA |
|  | rs7225002 | 41544850 | 1.39e-05 | rs1981997 | 0.44 |
|  | rs2532274 | 41602941 | 2.26e-09 | rs1981997 | 0.13 |
|  | rs2532269 | 41605885 | 7.62e-10 | rs1981997 | 0.06 |
|  | rs2668692 | 41648797 | 2.3e-09 | rs1981997 | NA |
|  | rs169201 | 42145386 | 5.26e-08 |  |  |
|  | rs199533 | 42184098 | 1.11e-07 | rs169201 | 0.43 |
|  | rs415430 | 42214305 | 4.43e-08 | rs169201 | 0.41 |
| 19p13 | rs12610495 | 4668672 | 1.54e-07 |  |  |
|  | rs2109069 | 4670443 | 3.98e-07 | rs12610495 | 0.95 |

**^a^Exception: Chr. 11 SNPs adjusted for rs35705950, which is not on GWAS SNP nor imputation panel but is well-replicated strongest association with IIP across that region.**

**^b^All adjusted for sex; “best SNP” defined by meta-analysis p-value, not “joint” p-value**

**S5: HLA Allele Imputation Accuracy Summary**

|  | **SNP Counts** | | **Posterior Probabilities** | | |
| --- | --- | --- | --- | --- | --- |
| **HLA Locus** | **HIBAG^a^** | **Study Data^b^** | **Mean ±SD^c^** | **Median** | **Mode** |
| A | 273 | 218 | 0.95 ±0.08 | 0.99 | 1.00 |
| B | 341 | 250 | 0.91 ±0.15 | 0.99 | 1.00 |
| C | 356 | 257 | 0.97 ±0.09 | 1.00 | 1.00 |
| DPB1 | 279 | 201 | 0.86 ±0.15 | 0.92 | 1.00 |
| DQA1 | 349 | 256 | 0.89 ±0.15 | 0.94 | 0.99 |
| DQB1 | 356 | 259 | 0.92 ±0.13 | 0.97 | 1.00 |
| DRB1 | 327 | 241 | 0.81 ±0.21 | 0.89 | 1.00 |

**^a^Number of HIBAG European Reference Panel SNPs used to develop imputation probabilities**

**^b^Number of SNPs available from GWAS for HLA allele imputation**

**^c^Mean± Standard Deviation**

**S6: HLA Allele Association with IIP**

|  | **Dosage Frequency** | | **Best Guess Allele Count** | |  |  |
| --- | --- | --- | --- | --- | --- | --- |
| **HLA Allele** | **Cases** | **Controls** | **Cases** | **Controls** | **OR (95% CI) ^a^** | **P-value^b^** |
| A*01:01 | 0.177 | 0.152 | 573 | 1433 | 1.19 (1.06-1.32) | 2.04E-03 |
| A*03:01 | 0.130 | 0.142 | 425 | 1350 | 0.91 (0.80-1.02) | 1.09E-01 |
| A*31:01 | 0.030 | 0.033 | 97 | 317 | 0.84 (0.66-1.07) | 1.59E-01 |
| A*30:02 | 0.008 | 0.010 | 24 | 94 | 0.75 (0.47-1.19) | 2.14E-01 |
| A*24:02 | 0.082 | 0.088 | 275 | 854 | 0.91 (0.78-1.06) | 2.16E-01 |
| A*66:01 | 0.004 | 0.003 | 12 | 25 | 1.55 (0.69-3.47) | 2.88E-01 |
| A*02:01 | 0.274 | 0.288 | 894 | 2726 | 0.96 (0.87-1.05) | 3.31E-01 |
| A*29:02 | 0.041 | 0.038 | 134 | 359 | 1.10 (0.89-1.35) | 3.67E-01 |
| A*68:01 | 0.035 | 0.031 | 114 | 295 | 1.11 (0.88-1.39) | 3.75E-01 |
| A*30:01 | 0.013 | 0.012 | 43 | 108 | 1.16 (0.81-1.67) | 4.12E-01 |
| A*29:01 | 0.002 | 0.003 | 6 | 25 | 0.69 (0.27-1.75) | 4.36E-01 |
| A*69:01 | 0.001 | 0.001 | 2 | 12 | 0.55 (0.10-2.97) | 4.85E-01 |
| A*32:01 | 0.033 | 0.035 | 106 | 326 | 0.92 (0.73-1.16) | 4.86E-01 |
| A*02:05 | 0.008 | 0.009 | 27 | 83 | 0.85 (0.53-1.37) | 5.07E-01 |
| A*11:01 | 0.062 | 0.058 | 203 | 544 | 1.06 (0.89-1.26) | 5.21E-01 |
| A*25:01 | 0.021 | 0.020 | 72 | 201 | 1.10 (0.80-1.51) | 5.54E-01 |
| A*33:01 | 0.008 | 0.007 | 28 | 71 | 1.14 (0.70-1.83) | 6.02E-01 |
| A*26:01 | 0.029 | 0.028 | 92 | 255 | 1.07 (0.82-1.41) | 6.21E-01 |
| A*33:03 | 0.002 | 0.002 | 6 | 22 | 0.81 (0.31-2.09) | 6.56E-01 |
| A*68:02 | 0.007 | 0.006 | 23 | 58 | 1.08 (0.65-1.78) | 7.78E-01 |
| A*23:01 | 0.019 | 0.017 | 60 | 158 | 1.04 (0.76-1.41) | 8.24E-01 |
| A*30:04 | 0.001 | 0.002 | 5 | 16 | 0.96 (0.30-3.12) | 9.48E-01 |
| B*35:03 | 0.011 | 0.018 | 28 | 146 | 0.50 (0.32-0.79) | 2.58E-03 |
| B*07:02 | 0.139 | 0.121 | 455 | 1139 | 1.20 (1.07-1.36) | 3.05E-03 |
| B*15:01 | 0.058 | 0.073 | 187 | 687 | 0.78 (0.66-0.93) | 5.68E-03 |
| B*39:01 | 0.009 | 0.012 | 30 | 123 | 0.55 (0.32-0.94) | 3.03E-02 |
| B*08:01 | 0.126 | 0.113 | 407 | 1059 | 1.12 (0.98-1.27) | 8.94E-02 |
| B*38:01 | 0.013 | 0.016 | 45 | 157 | 0.71 (0.48-1.06) | 9.23E-02 |
| B*35:01 | 0.046 | 0.052 | 153 | 498 | 0.84 (0.69-1.04) | 1.09E-01 |
| B*47:01 | 0.001 | 0.003 | 4 | 26 | 0.41 (0.13-1.28) | 1.26E-01 |
| B*41:02 | 0.006 | 0.004 | 20 | 36 | 1.57 (0.88-2.81) | 1.30E-01 |
| B*55:01 | 0.019 | 0.016 | 59 | 150 | 1.27 (0.93-1.73) | 1.35E-01 |
| B*40:01 | 0.055 | 0.061 | 178 | 572 | 0.87 (0.73-1.05) | 1.39E-01 |
| B*37:01 | 0.016 | 0.013 | 52 | 124 | 1.22 (0.87-1.71) | 2.43E-01 |
| B*13:02 | 0.026 | 0.023 | 87 | 220 | 1.17 (0.90-1.52) | 2.49E-01 |
| B*44:03 | 0.057 | 0.053 | 187 | 507 | 1.11 (0.93-1.33) | 2.65E-01 |
| B*57:01 | 0.040 | 0.037 | 131 | 351 | 1.12 (0.91-1.39) | 2.83E-01 |
| B*39:06 | 0.006 | 0.007 | 20 | 61 | 0.73 (0.40-1.33) | 3.04E-01 |
| B*14:02 | 0.022 | 0.024 | 70 | 230 | 0.86 (0.65-1.14) | 3.05E-01 |
| B*53:01 | 0.002 | 0.003 | 5 | 23 | 0.59 (0.21-1.66) | 3.20E-01 |
| B*44:02 | 0.091 | 0.100 | 297 | 940 | 0.93 (0.81-1.07) | 3.26E-01 |
| B*15:17 | 0.004 | 0.003 | 14 | 27 | 1.37 (0.70-2.71) | 3.62E-01 |
| B*27:05 | 0.039 | 0.037 | 134 | 369 | 1.09 (0.87-1.37) | 4.33E-01 |
| B*15:18 | 0.003 | 0.003 | 10 | 33 | 0.71 (0.28-1.82) | 4.76E-01 |
| B*49:01 | 0.014 | 0.015 | 44 | 138 | 0.88 (0.62-1.26) | 4.93E-01 |
| B*44:05 | 0.004 | 0.005 | 12 | 42 | 0.79 (0.39-1.59) | 5.08E-01 |
| B*51:01 | 0.053 | 0.051 | 179 | 490 | 1.05 (0.87-1.27) | 5.92E-01 |
| B*58:01 | 0.006 | 0.006 | 19 | 59 | 0.88 (0.52-1.49) | 6.41E-01 |
| B*35:08 | 0.005 | 0.005 | 17 | 62 | 0.83 (0.35-1.99) | 6.81E-01 |
| B*50:01 | 0.010 | 0.010 | 31 | 99 | 0.91 (0.59-1.41) | 6.82E-01 |
| B*35:02 | 0.008 | 0.008 | 28 | 87 | 0.90 (0.53-1.54) | 7.01E-01 |
| B*18:01 | 0.046 | 0.045 | 150 | 428 | 1.04 (0.85-1.27) | 7.10E-01 |
| B*56:01 | 0.004 | 0.005 | 18 | 49 | 0.90 (0.45-1.78) | 7.60E-01 |
| B*52:01 | 0.008 | 0.007 | 24 | 63 | 1.07 (0.66-1.73) | 7.89E-01 |
| B*45:01 | 0.006 | 0.006 | 21 | 50 | 1.06 (0.61-1.84) | 8.46E-01 |
| B*41:01 | 0.004 | 0.004 | 13 | 38 | 0.94 (0.45-1.94) | 8.65E-01 |
| B*27:02 | 0.005 | 0.005 | 6 | 18 | 0.97 (0.39-2.41) | 9.39E-01 |
| B*07:05 | 0.003 | 0.003 | 7 | 25 | 1.03 (0.42-2.52) | 9.57E-01 |
| B*40:02 | 0.012 | 0.012 | 43 | 123 | 0.99 (0.67-1.46) | 9.68E-01 |
| B*14:01 | 0.009 | 0.009 | 30 | 87 | 0.99 (0.65-1.51) | 9.71E-01 |
| C*04:01 | 0.087 | 0.105 | 283 | 985 | 0.80 (0.70-0.93) | 2.65E-03 |
| C*07:02 | 0.151 | 0.135 | 487 | 1266 | 1.15 (1.02-1.29) | 2.04E-02 |
| C*03:04 | 0.074 | 0.086 | 234 | 793 | 0.83 (0.71-0.97) | 2.11E-02 |
| C*15:02 | 0.026 | 0.021 | 89 | 204 | 1.30 (0.98-1.71) | 6.66E-02 |
| C*16:01 | 0.043 | 0.038 | 140 | 364 | 1.15 (0.93-1.42) | 1.89E-01 |
| C*06:02 | 0.096 | 0.090 | 310 | 841 | 1.10 (0.95-1.26) | 1.96E-01 |
| C*07:01 | 0.169 | 0.156 | 549 | 1466 | 1.07 (0.96-1.20) | 2.08E-01 |
| C*05:01 | 0.093 | 0.104 | 299 | 978 | 0.92 (0.80-1.05) | 2.24E-01 |
| C*17:01 | 0.009 | 0.007 | 31 | 64 | 1.35 (0.82-2.23) | 2.35E-01 |
| C*12:03 | 0.041 | 0.043 | 131 | 406 | 0.91 (0.74-1.12) | 3.83E-01 |
| C*08:02 | 0.031 | 0.033 | 100 | 307 | 0.92 (0.73-1.17) | 4.93E-01 |
| C*16:02 | 0.002 | 0.003 | 7 | 28 | 0.75 (0.30-1.87) | 5.37E-01 |
| C*14:02 | 0.010 | 0.011 | 31 | 99 | 0.88 (0.57-1.34) | 5.38E-01 |
| C*07:04 | 0.015 | 0.016 | 49 | 145 | 0.91 (0.65-1.27) | 5.76E-01 |
| C*03:02 | 0.001 | 0.001 | 4 | 12 | 0.73 (0.21-2.61) | 6.33E-01 |
| C*12:02 | 0.008 | 0.007 | 25 | 63 | 1.12 (0.70-1.80) | 6.39E-01 |
| C*15:05 | 0.003 | 0.003 | 10 | 27 | 1.16 (0.51-2.63) | 7.23E-01 |
| C*01:02 | 0.034 | 0.034 | 109 | 319 | 1.04 (0.83-1.30) | 7.25E-01 |
| C*02:02 | 0.045 | 0.047 | 149 | 437 | 0.98 (0.81-1.20) | 8.47E-01 |
| C*03:03 | 0.056 | 0.057 | 186 | 545 | 0.99 (0.83-1.19) | 9.22E-01 |
| C*16:04 | 0.002 | 0.002 | 3 | 13 | 0.98 (0.19-5.05) | 9.79E-01 |
| DPB1*19:01 | 0.006 | 0.008 | 19 | 77 | 0.67 (0.40-1.13) | 1.34E-01 |
| DPB1*15:01 | 0.006 | 0.008 | 18 | 76 | 0.69 (0.41-1.17) | 1.68E-01 |
| DPB1*04:01 | 0.433 | 0.420 | 1452 | 4092 | 1.06 (0.98-1.15) | 1.74E-01 |
| DPB1*09:01 | 0.006 | 0.008 | 26 | 85 | 0.66 (0.32-1.35) | 2.53E-01 |
| DPB1*04:02 | 0.107 | 0.113 | 354 | 1082 | 0.93 (0.81-1.07) | 2.98E-01 |
| DPB1*10:01 | 0.013 | 0.015 | 39 | 139 | 0.82 (0.55-1.21) | 3.09E-01 |
| DPB1*03:01 | 0.096 | 0.091 | 379 | 1061 | 1.07 (0.92-1.26) | 3.87E-01 |
| DPB1*05:01 | 0.021 | 0.020 | 68 | 189 | 1.11 (0.84-1.48) | 4.68E-01 |
| DPB1*14:01 | 0.013 | 0.015 | 43 | 139 | 0.86 (0.55-1.33) | 4.91E-01 |
| DPB1*01:01 | 0.051 | 0.054 | 164 | 509 | 0.94 (0.78-1.13) | 5.12E-01 |
| DPB1*11:01 | 0.023 | 0.022 | 75 | 207 | 1.07 (0.81-1.41) | 6.58E-01 |
| DPB1*13:01 | 0.016 | 0.016 | 53 | 148 | 1.04 (0.74-1.44) | 8.39E-01 |
| DPB1*02:01 | 0.132 | 0.132 | 418 | 1206 | 0.99 (0.87-1.13) | 8.49E-01 |
| DPB1*104:01 | 0.016 | 0.015 | 56 | 160 | 0.97 (0.66-1.43) | 8.87E-01 |
| DPB1*16:01 | 0.006 | 0.006 | 22 | 62 | 0.98 (0.55-1.75) | 9.50E-01 |
| DPB1*17:01 | 0.014 | 0.014 | 44 | 127 | 0.99 (0.69-1.42) | 9.51E-01 |
| DQA1*01:02 | 0.218 | 0.183 | 703 | 1725 | 1.28 (1.15-1.42) | 4.84E-06 |
| DQA1*03:01 | 0.085 | 0.097 | 284 | 927 | 0.84 (0.72-0.98) | 3.01E-02 |
| DQA1*03:02 | 0.014 | 0.010 | 49 | 97 | 1.54 (0.98-2.43) | 6.43E-02 |
| DQA1*01:03 | 0.066 | 0.074 | 202 | 665 | 0.86 (0.72-1.03) | 9.81E-02 |
| DQA1*03:03 | 0.072 | 0.081 | 228 | 744 | 0.87 (0.73-1.04) | 1.22E-01 |
| DQA1*01:01 | 0.106 | 0.115 | 336 | 1062 | 0.91 (0.79-1.04) | 1.49E-01 |
| DQA1*06:01 | 0.003 | 0.002 | 8 | 15 | 1.96 (0.71-5.41) | 1.92E-01 |
| DQA1*02:01 | 0.143 | 0.136 | 458 | 1273 | 1.07 (0.95-1.21) | 2.60E-01 |
| DQA1*01:05 | 0.008 | 0.006 | 31 | 66 | 1.43 (0.75-2.72) | 2.77E-01 |
| DQA1*05:05 | 0.115 | 0.121 | 380 | 1148 | 0.94 (0.83-1.08) | 3.89E-01 |
| DQA1*05:01 | 0.119 | 0.123 | 385 | 1154 | 0.95 (0.84-1.08) | 4.59E-01 |
| DQA1*04:01 | 0.026 | 0.026 | 83 | 250 | 0.94 (0.72-1.22) | 6.27E-01 |
| DQA1*01:04 | 0.023 | 0.022 | 83 | 229 | 1.02 (0.72-1.45) | 8.96E-01 |
| DQB1*06:02 | 0.152 | 0.117 | 505 | 1131 | 1.40 (1.24-1.58) | 6.05E-08 |
| DQB1*06:03 | 0.060 | 0.069 | 182 | 629 | 0.81 (0.67-0.99) | 3.60E-02 |
| DQB1*03:02 | 0.090 | 0.102 | 294 | 968 | 0.86 (0.75-0.99) | 4.06E-02 |
| DQB1*03:01 | 0.181 | 0.195 | 596 | 1869 | 0.92 (0.83-1.03) | 1.34E-01 |
| DQB1*06:01 | 0.007 | 0.005 | 23 | 43 | 1.39 (0.81-2.37) | 2.29E-01 |
| DQB1*02:02 | 0.106 | 0.100 | 343 | 929 | 1.08 (0.94-1.24) | 2.61E-01 |
| DQB1*03:03 | 0.051 | 0.046 | 167 | 437 | 1.12 (0.92-1.38) | 2.62E-01 |
| DQB1*05:01 | 0.114 | 0.121 | 370 | 1137 | 0.93 (0.82-1.06) | 2.67E-01 |
| DQB1*05:04 | 0.001 | 0.001 | 2 | 13 | 0.19 (0.01-3.78) | 2.74E-01 |
| DQB1*02:01 | 0.119 | 0.123 | 385 | 1157 | 0.95 (0.84-1.08) | 4.64E-01 |
| DQB1*05:02 | 0.018 | 0.019 | 53 | 156 | 0.92 (0.65-1.29) | 6.18E-01 |
| DQB1*04:02 | 0.027 | 0.027 | 88 | 255 | 0.95 (0.73-1.22) | 6.60E-01 |
| DQB1*06:09 | 0.009 | 0.010 | 27 | 89 | 0.92 (0.56-1.51) | 7.46E-01 |
| DQB1*06:04 | 0.033 | 0.033 | 107 | 304 | 1.03 (0.79-1.33) | 8.43E-01 |
| DQB1*05:03 | 0.027 | 0.027 | 85 | 239 | 1.02 (0.76-1.35) | 9.12E-01 |
| DRB1*15:01 | 0.158 | 0.122 | 513 | 1151 | 1.37 (1.22-1.54) | 1.29E-07 |
| DRB1*04:01 | 0.081 | 0.098 | 283 | 982 | 0.78 (0.66-0.92) | 2.97E-03 |
| DRB1*11:04 | 0.025 | 0.029 | 38 | 156 | 0.67 (0.45-1.00) | 4.77E-02 |
| DRB1*13:01 | 0.057 | 0.065 | 180 | 621 | 0.83 (0.69-1.00) | 5.06E-02 |
| DRB1*01:01 | 0.084 | 0.095 | 293 | 956 | 0.88 (0.76-1.02) | 8.88E-02 |
| DRB1*09:01 | 0.014 | 0.010 | 45 | 98 | 1.39 (0.92-2.10) | 1.21E-01 |
| DRB1*08:04 | 0.002 | 0.001 | 8 | 11 | 1.99 (0.67-5.89) | 2.14E-01 |
| DRB1*15:02 | 0.007 | 0.005 | 23 | 46 | 1.39 (0.82-2.34) | 2.21E-01 |
| DRB1*08:03 | 0.002 | 0.001 | 8 | 12 | 2.11 (0.63-6.99) | 2.24E-01 |
| DRB1*04:04 | 0.030 | 0.033 | 114 | 371 | 0.85 (0.64-1.13) | 2.59E-01 |
| DRB1*10:01 | 0.010 | 0.008 | 34 | 75 | 1.29 (0.83-2.02) | 2.64E-01 |
| DRB1*07:01 | 0.142 | 0.136 | 462 | 1272 | 1.07 (0.95-1.21) | 2.75E-01 |
| DRB1*08:01 | 0.023 | 0.024 | 76 | 241 | 0.88 (0.66-1.16) | 3.58E-01 |
| DRB1*03:01 | 0.120 | 0.124 | 389 | 1159 | 0.96 (0.84-1.09) | 4.77E-01 |
| DRB1*04:08 | 0.005 | 0.004 | 6 | 19 | 1.62 (0.37-7.13) | 5.22E-01 |
| DRB1*11:02 | 0.003 | 0.003 | 8 | 30 | 0.77 (0.28-2.10) | 6.03E-01 |
| DRB1*11:01 | 0.054 | 0.056 | 246 | 731 | 0.96 (0.76-1.20) | 7.05E-01 |
| DRB1*01:03 | 0.009 | 0.008 | 12 | 17 | 1.13 (0.54-2.34) | 7.48E-01 |
| DRB1*16:01 | 0.014 | 0.014 | 45 | 133 | 0.94 (0.65-1.36) | 7.56E-01 |
| DRB1*04:02 | 0.008 | 0.008 | 23 | 72 | 0.91 (0.47-1.73) | 7.64E-01 |
| DRB1*04:05 | 0.007 | 0.007 | 22 | 61 | 1.07 (0.56-2.03) | 8.43E-01 |
| DRB1*04:03 | 0.011 | 0.011 | 15 | 31 | 0.93 (0.42-2.03) | 8.45E-01 |
| DRB1*13:02 | 0.042 | 0.042 | 140 | 402 | 1.02 (0.82-1.27) | 8.50E-01 |
| DRB1*04:07 | 0.014 | 0.014 | 39 | 120 | 0.96 (0.56-1.65) | 8.79E-01 |
| DRB1*13:03 | 0.010 | 0.010 | 31 | 87 | 1.03 (0.67-1.59) | 8.81E-01 |
| DRB1*12:01 | 0.014 | 0.015 | 44 | 133 | 1.01 (0.70-1.47) | 9.45E-01 |
| DRB1*14:01 | 0.025 | 0.026 | 86 | 241 | 0.99 (0.74-1.33) | 9.59E-01 |
| DRB1*01:02 | 0.013 | 0.012 | 40 | 111 | 1.00 (0.68-1.47) | 9.82E-01 |

**^a^Odds ratio and 95% CI**

**^b^Gender, PC1, PC2, PC3 as covariates; required 10-best guess copies to perform test**

| **Start Site** | **End Site** | **Length** | **Genes Covered on AmpliSeq** |
| --- | --- | --- | --- |
| 31304507 | 32893160 | 1588653 | *BAG6;PRRC2A;HLA-DQB1;CLIC1;HCG23;LST1;DDAH2;FKBPL;PPT2;LY6G6E;NFKBIL1;HLA-C;HSPA1L;HLA-DQA2;MIR4646;EGFL8;C2;EHMT2;SNORD48;MICA;NCR3;LY6G6C;LY6G6D;LSM2;VWA7;HLA-B;HLA-DOB;HLA-DQA1;C4B_2;LOC102060414;SNORA38;HLA-DRA;C6orf48;VARS;C4A;LY6G6F;SAPCD1;APOM;CFB;STK19;MICA;LTB;HLA-DQB2;HLA-DRB6;RNF5P1;LTA;AGPAT1;C6orf25;PBX2;APOM;ABHD16A;STK19;RNF5;NEU1;LOC100507547;ZBTB12;MSH5-SAPCD1;ATP6V1G2-DDX39B;CSNK2B;GPSM3;ABHD16A;SNORD117;C6orf10;PRRT1;LY6G5B;TNF;DXO;HSPA1B;TNXB;NELFE;SNORD52;MIR1236;CYP21A1P;HSPA1A;DDX39B;MIR6721;AGER;HLA-DRB1;GPANK1;CYP21A2;MIR6891;HCG26;LY6G5C;NOTCH4;SNORD84;MIR6832;AIF1;PPT2-EGFL8;LST1;ATP6V1G2;EGFL8;AGER;ATF6B;MICB;MIR6833;C6orf47;TNXA;HLA-DRB5;SKIV2L;BTNL2;HCP5;MSH5;DDX39B;SLC44A4;MCCD1;C4B* |

**S7: Chromosome 6p21 genes studied via RNA-seq**

**^a^We defined target region by extending from either side of rs7887. We extended the region sequentially if there was another SNP with P<1x10^-04^ within 500 kb of the current boundary. If either end of a resulting region was within 20 kb of a gene, the bounds were extended 20 kb beyond the end of that gene. Finally, the region was extended an additional 20 kb on either end.**

**S8: Differential Expression by Case-Control Status**

| **Gene** | **P-Value** | **FDR - Q** | **logFC** | **logCPM** | **LR** |  | **gene** | **PValue** | **FDR** | **logFC** | **logCPM** | **LR** |
| --- | --- | --- | --- | --- | --- | --- | --- | --- | --- | --- | --- | --- |
| C6orf25 | 3.90E-17 | 2.57E-15 | -1.93 | 5.08 | 70.83 |  | HSPA1A | 0.02 | 0.04 | 0.51 | 15.02 | 5.21 |
| VWA7 | 8.37E-15 | 2.76E-13 | 1.54 | 9.02 | 60.25 |  | PPT2 | 0.03 | 0.06 | 0.20 | 11.45 | 4.75 |
| LY6G6F | 3.75E-14 | 8.25E-13 | -2.32 | 7.66 | 57.30 |  | DDX39B | 0.03 | 0.06 | -0.18 | 14.69 | 4.48 |
| LTA | 8.63E-14 | 1.42E-12 | 1.42 | 6.37 | 55.66 |  | C6orf48 | 0.04 | 0.07 | -0.17 | 14.06 | 4.29 |
| NOTCH4 | 1.90E-13 | 2.51E-12 | -1.17 | 6.32 | 54.11 |  | BAG6 | 0.05 | 0.08 | -0.15 | 13.02 | 3.96 |
| LTB | 1.26E-12 | 1.39E-11 | 1.31 | 9.66 | 50.39 |  | NCR3 | 0.05 | 0.08 | 0.32 | 6.90 | 3.89 |
| AGER | 2.08E-12 | 1.96E-11 | -1.45 | 16.20 | 49.41 |  | TNXB | 0.06 | 0.10 | 0.70 | 13.71 | 3.57 |
| SLC44A4 | 1.43E-11 | 1.18E-10 | 1.39 | 14.87 | 45.63 |  | HLA-DQA2 | 0.07 | 0.11 | 0.68 | 7.20 | 3.39 |
| HLA-DOB | 3.23E-09 | 2.37E-08 | 1.05 | 10.97 | 35.04 |  | LST1 | 0.08 | 0.12 | -0.26 | 12.45 | 3.16 |
| C2 | 3.55E-08 | 2.34E-07 | -0.62 | 13.45 | 30.38 |  | GPANK1 | 0.09 | 0.13 | -0.15 | 10.34 | 2.90 |
| LY6G5B | 1.23E-07 | 7.39E-07 | -0.52 | 11.76 | 27.97 |  | CSNK2B | 0.09 | 0.13 | -0.12 | 12.95 | 2.92 |
| GPSM3 | 7.96E-06 | 3.75E-05 | -0.43 | 12.60 | 19.95 |  | EHMT2 | 0.14 | 0.21 | 0.12 | 12.00 | 2.17 |
| SAPCD1 | 7.01E-06 | 3.75E-05 | 0.46 | 8.04 | 20.19 |  | HLA-C | 0.15 | 0.21 | -0.36 | 15.20 | 2.11 |
| C4B | 7.51E-06 | 3.75E-05 | 0.61 | 14.51 | 20.06 |  | MSH5 | 0.16 | 0.22 | 0.16 | 9.57 | 2.01 |
| TNXA | 8.59E-06 | 3.78E-05 | 0.74 | 11.42 | 19.80 |  | HLA-DQA1 | 0.24 | 0.34 | 0.15 | 16.04 | 1.35 |
| HSPA1B | 1.75E-05 | 7.20E-05 | 0.74 | 12.51 | 18.45 |  | TNF | 0.28 | 0.38 | 0.18 | 9.24 | 1.16 |
| MICA | 1.15E-04 | 4.21E-04 | -0.34 | 11.46 | 14.88 |  | ABHD16A | 0.34 | 0.45 | -0.08 | 12.56 | 0.90 |
| C4A | 1.14E-04 | 4.21E-04 | 0.64 | 14.85 | 14.89 |  | LY6G6C | 0.37 | 0.48 | -0.20 | 4.57 | 0.80 |
| ATP6V1G2 | 1.41E-04 | 4.88E-04 | 0.51 | 7.87 | 14.50 |  | LSM2 | 0.42 | 0.53 | 0.06 | 13.61 | 0.66 |
| HSPA1L | 2.66E-04 | 8.76E-04 | 0.46 | 6.33 | 13.30 |  | CYP21A1P | 0.44 | 0.54 | -0.34 | 10.51 | 0.61 |
| SNORA38 | 2.90E-04 | 9.10E-04 | 0.47 | 13.92 | 13.14 |  | SKIV2L | 0.45 | 0.55 | 0.07 | 12.40 | 0.56 |
| VARS | 3.35E-04 | 1.01E-03 | -0.32 | 13.45 | 12.86 |  | PBX2 | 0.49 | 0.57 | -0.06 | 12.65 | 0.48 |
| PRRT1 | 7.54E-04 | 2.16E-03 | 0.72 | 8.37 | 11.35 |  | ATF6B | 0.48 | 0.57 | -0.05 | 13.81 | 0.50 |
| MICB | 1.20E-03 | 3.29E-03 | -0.38 | 10.40 | 10.49 |  | HLA-DRA | 0.50 | 0.58 | 0.08 | 18.58 | 0.45 |
| NEU1 | 1.26E-03 | 3.34E-03 | -0.26 | 13.17 | 10.39 |  | HLA-DRB1 | 0.52 | 0.59 | -0.27 | 16.44 | 0.42 |
| APOM | 2.47E-03 | 0.01 | -0.30 | 7.94 | 9.16 |  | STK19 | 0.57 | 0.64 | 0.05 | 11.33 | 0.31 |
| AIF1 | 4.44E-03 | 0.01 | -0.36 | 8.59 | 8.10 |  | RNF5 | 0.63 | 0.69 | 0.05 | 11.80 | 0.24 |
| C6orf47 | 0.01 | 0.02 | -0.23 | 12.17 | 7.29 |  | FKBPL | 0.69 | 0.75 | 0.06 | 6.10 | 0.16 |
| LY6G6D | 0.01 | 0.02 | -0.51 | 4.50 | 7.03 |  | DOM3Z | 0.74 | 0.79 | -0.04 | 5.95 | 0.11 |
| EGFL8 | 0.01 | 0.02 | 0.47 | 8.17 | 7.03 |  | CYP21A2 | 0.84 | 0.88 | -0.05 | 11.15 | 0.04 |
| CFB | 0.01 | 0.02 | 0.48 | 13.71 | 7.04 |  | DDAH2 | 0.85 | 0.88 | -0.03 | 8.37 | 0.04 |
| HLA-DQB2 | 0.01 | 0.02 | 0.68 | 8.59 | 6.71 |  | AGPAT1 | 0.93 | 0.94 | -0.01 | 14.02 | 0.01 |
| RNF5P1 | 0.01 | 0.03 | -0.35 | 9.74 | 6.00 |  | NFKBIL1 | 1.00 | 1.00 | 0.00 | 8.92 | 0.00 |

**S9: Differential Expression by Genotype at rs7887 Among Controls**

| **Gene** | **P-Value** | **Z Statistic** | **FDR (Q-value)** |  | **Gene** | **P-Value** | **Z Statistic** | **FDR (Q-value)** |
| --- | --- | --- | --- | --- | --- | --- | --- | --- |
| ABHD16A | 0.01 | -2.54 | 0.25 |  | NCR3 | 0.32 | -1.00 | 0.60 |
| ATP6V1G2 | 0.01 | -2.78 | 0.25 |  | VWA7 | 0.29 | -1.06 | 0.60 |
| HLA-DQA2 | 0.01 | -2.57 | 0.25 |  | MICA | 0.34 | -0.95 | 0.63 |
| AGER | 0.07 | -1.79 | 0.37 |  | BAG6 | 0.37 | -0.89 | 0.66 |
| ATF6B | 0.03 | -2.19 | 0.37 |  | RNF5P1 | 0.41 | -0.82 | 0.71 |
| C6orf47 | 0.08 | -1.74 | 0.37 |  | APOM | 0.48 | -0.71 | 0.71 |
| EGFL8 | 0.08 | -1.73 | 0.37 |  | C4B | 0.47 | 0.72 | 0.71 |
| HLA-DQA1 | 0.06 | 1.91 | 0.37 |  | LST1 | 0.46 | -0.75 | 0.71 |
| HLA-DRA | 0.04 | 2.10 | 0.37 |  | NEU1 | 0.45 | -0.76 | 0.71 |
| HSPA1L | 0.02 | -2.25 | 0.37 |  | RNF5 | 0.44 | -0.77 | 0.71 |
| MSH5 | 0.04 | -2.03 | 0.37 |  | SLC44A4 | 0.43 | -0.79 | 0.71 |
| PRRT1 | 0.07 | -1.83 | 0.37 |  | AIF1 | 0.52 | 0.64 | 0.74 |
| SKIV2L | 0.08 | -1.74 | 0.37 |  | C2 | 0.51 | -0.65 | 0.74 |
| STK19 | 0.09 | -1.70 | 0.37 |  | DDX39B | 0.55 | -0.60 | 0.74 |
| TNF | 0.06 | 1.85 | 0.37 |  | MICB | 0.55 | 0.60 | 0.74 |
| TNXB | 0.09 | -1.71 | 0.37 |  | SNORA38 | 0.54 | 0.62 | 0.74 |
| LY6G6F | 0.10 | -1.64 | 0.39 |  | GPSM3 | 0.57 | -0.57 | 0.75 |
| HLA-DQB2 | 0.13 | 1.50 | 0.43 |  | LTA | 0.60 | -0.52 | 0.78 |
| LTB | 0.14 | 1.49 | 0.43 |  | VARS | 0.62 | 0.49 | 0.79 |
| LY6G6D | 0.13 | -1.51 | 0.43 |  | HLA-DOB | 0.64 | -0.46 | 0.80 |
| NFKBIL1 | 0.12 | -1.55 | 0.43 |  | CYP21A2 | 0.68 | 0.41 | 0.81 |
| C6orf25 | 0.17 | -1.36 | 0.50 |  | HSPA1A | 0.67 | -0.43 | 0.81 |
| EHMT2 | 0.17 | -1.38 | 0.50 |  | SAPCD1 | 0.69 | -0.40 | 0.81 |
| HLA-C | 0.21 | -1.26 | 0.55 |  | C6orf48 | 0.74 | -0.33 | 0.82 |
| HSPA1B | 0.22 | 1.23 | 0.55 |  | DOM3Z | 0.73 | -0.35 | 0.82 |
| PPT2 | 0.22 | -1.23 | 0.55 |  | FKBPL | 0.74 | -0.33 | 0.82 |
| AGPAT1 | 0.26 | -1.13 | 0.59 |  | LY6G5B | 0.71 | -0.37 | 0.82 |
| C4A | 0.25 | 1.15 | 0.59 |  | CSNK2B | 0.80 | 0.25 | 0.87 |
| PBX2 | 0.25 | -1.15 | 0.59 |  | CYP21A1P | 0.87 | -0.16 | 0.92 |
| CFB | 0.32 | 1.00 | 0.60 |  | HLA-DRB1 | 0.88 | -0.15 | 0.92 |
| DDAH2 | 0.30 | -1.03 | 0.60 |  | LY6G6C | 0.95 | 0.06 | 0.96 |
| GPANK1 | 0.27 | -1.09 | 0.60 |  | NOTCH4 | 0.96 | -0.04 | 0.96 |
| LSM2 | 0.30 | 1.03 | 0.60 |  | TNXA | 0.96 | -0.05 | 0.96 |

**S10: Differential Expression by Imputed Number of Copies of DRB1*15:01^a^ Among Cases (RNA-seq)**

| **Gene** | **P-Value** | **Z Statistic** | **FDR (Q-value)** |  | **Gene** | **P-Value** | **Z Statistic** | **FDR (Q-value)** |
| --- | --- | --- | --- | --- | --- | --- | --- | --- |
| AIF1 | 1.78E-07 | -5.22 | 1.18E-05 |  | HSPA1A | 0.27 | -1.09 | 0.53 |
| HLA-DRA | 3.92E-03 | -2.88 | 0.13 |  | CSNK2B | 0.28 | -1.07 | 0.53 |
| HLA-DRB1 | 0.01 | 2.46 | 0.18 |  | GPANK1 | 0.30 | -1.03 | 0.56 |
| MICA | 0.01 | -2.47 | 0.18 |  | C6orf25 | 0.41 | -0.83 | 0.60 |
| NEU1 | 0.01 | -2.51 | 0.18 |  | EHMT2 | 0.38 | -0.88 | 0.60 |
| C2 | 0.03 | -2.18 | 0.22 |  | LY6G5B | 0.36 | -0.91 | 0.60 |
| C6orf47 | 0.04 | -2.09 | 0.22 |  | LY6G6D | 0.35 | -0.93 | 0.60 |
| DOM3Z | 0.04 | -2.06 | 0.22 |  | PRRT1 | 0.37 | -0.90 | 0.60 |
| FKBPL | 0.03 | -2.19 | 0.22 |  | RNF5 | 0.38 | -0.88 | 0.60 |
| LSM2 | 0.04 | -2.07 | 0.22 |  | RNF5P1 | 0.39 | 0.86 | 0.60 |
| LST1 | 0.02 | -2.32 | 0.22 |  | SAPCD1 | 0.40 | -0.84 | 0.60 |
| STK19 | 0.03 | -2.12 | 0.22 |  | SNORA38 | 0.41 | -0.83 | 0.60 |
| DDAH2 | 0.05 | 1.97 | 0.23 |  | GPSM3 | 0.44 | -0.77 | 0.63 |
| TNXA | 0.05 | 1.98 | 0.23 |  | APOM | 0.49 | -0.70 | 0.68 |
| ABHD16A | 0.06 | -1.85 | 0.27 |  | AGER | 0.56 | -0.59 | 0.75 |
| TNXB | 0.07 | 1.84 | 0.27 |  | ATP6V1G2 | 0.56 | -0.58 | 0.75 |
| C4A | 0.08 | 1.78 | 0.28 |  | HSPA1L | 0.59 | 0.55 | 0.77 |
| C6orf48 | 0.07 | -1.78 | 0.28 |  | CYP21A1P | 0.62 | 0.49 | 0.79 |
| AGPAT1 | 0.11 | -1.61 | 0.35 |  | HLA-DQB2 | 0.63 | -0.48 | 0.79 |
| VARS | 0.11 | -1.61 | 0.35 |  | SKIV2L | 0.62 | 0.50 | 0.79 |
| ATF6B | 0.12 | -1.54 | 0.37 |  | SLC44A4 | 0.65 | -0.46 | 0.79 |
| HSPA1B | 0.12 | -1.55 | 0.37 |  | CYP21A2 | 0.66 | 0.43 | 0.80 |
| HLA-C | 0.13 | 1.51 | 0.38 |  | HLA-DQA2 | 0.70 | 0.39 | 0.82 |
| MSH5 | 0.14 | -1.47 | 0.39 |  | VWA7 | 0.71 | -0.37 | 0.82 |
| NOTCH4 | 0.16 | 1.42 | 0.39 |  | NCR3 | 0.79 | 0.27 | 0.90 |
| PBX2 | 0.15 | -1.44 | 0.39 |  | EGFL8 | 0.82 | 0.23 | 0.92 |
| HLA-DOB | 0.17 | -1.37 | 0.42 |  | CFB | 0.87 | 0.16 | 0.96 |
| DDX39B | 0.18 | -1.34 | 0.42 |  | LTA | 0.95 | -0.07 | 0.99 |
| HLA-DQA1 | 0.19 | -1.30 | 0.44 |  | LY6G6C | 0.93 | -0.08 | 0.99 |
| LTB | 0.22 | 1.22 | 0.46 |  | TNF | 0.92 | 0.10 | 0.99 |
| LY6G6F | 0.22 | -1.23 | 0.46 |  | BAG6 | 0.98 | -0.03 | 0.99 |
| MICB | 0.21 | -1.25 | 0.46 |  | NFKBIL1 | 0.97 | 0.04 | 0.99 |
| C4B | 0.26 | 1.13 | 0.52 |  | PPT2 | 1.00 | -0.01 | 1.00 |

**^a^There were 60 cases with expected number of 15:01 alleles = 0 and 27 cases with expected number of alleles = 1**

**S11: Differential Expression by Imputed Number of Copies of DQB1*06*02^a^ Among Cases (RNA-seq)**

| **Gene** | **P-Value** | **Z Statistic** | **FDR (Q-value)** |  | **Gene** | **P-Value** | **Z Statistic** | **FDR (Q-value)** |
| --- | --- | --- | --- | --- | --- | --- | --- | --- |
| AIF1 | 1.74E-07 | -5.22 | 1.15E-05 |  | CSNK2B | 0.28 | -1.09 | 0.52 |
| HLA-DRA | 3.62E-03 | -2.91 | 0.12 |  | HSPA1A | 0.28 | -1.09 | 0.52 |
| HLA-DRB1 | 0.01 | 2.48 | 0.17 |  | GPANK1 | 0.31 | -1.02 | 0.56 |
| MICA | 0.01 | -2.48 | 0.17 |  | C6orf25 | 0.41 | -0.83 | 0.60 |
| NEU1 | 0.01 | -2.54 | 0.17 |  | EHMT2 | 0.38 | -0.87 | 0.60 |
| LST1 | 0.02 | -2.35 | 0.21 |  | LY6G5B | 0.38 | -0.88 | 0.60 |
| C2 | 0.03 | -2.20 | 0.23 |  | LY6G6D | 0.36 | -0.92 | 0.60 |
| C6orf47 | 0.04 | -2.10 | 0.23 |  | PRRT1 | 0.37 | -0.89 | 0.60 |
| DOM3Z | 0.04 | -2.03 | 0.23 |  | RNF5 | 0.38 | -0.88 | 0.60 |
| FKBPL | 0.03 | -2.18 | 0.23 |  | RNF5P1 | 0.40 | 0.85 | 0.60 |
| LSM2 | 0.04 | -2.06 | 0.23 |  | SAPCD1 | 0.41 | -0.82 | 0.60 |
| STK19 | 0.04 | -2.10 | 0.23 |  | SNORA38 | 0.41 | -0.82 | 0.60 |
| DDAH2 | 0.05 | 1.95 | 0.24 |  | GPSM3 | 0.42 | -0.80 | 0.61 |
| TNXA | 0.05 | 1.96 | 0.24 |  | APOM | 0.48 | -0.71 | 0.67 |
| ABHD16A | 0.06 | -1.86 | 0.27 |  | AGER | 0.54 | -0.62 | 0.74 |
| TNXB | 0.06 | 1.85 | 0.27 |  | ATP6V1G2 | 0.55 | -0.59 | 0.75 |
| C4A | 0.07 | 1.79 | 0.29 |  | HSPA1L | 0.58 | 0.55 | 0.77 |
| C6orf48 | 0.08 | -1.76 | 0.29 |  | CYP21A1P | 0.61 | 0.51 | 0.78 |
| AGPAT1 | 0.11 | -1.60 | 0.36 |  | SKIV2L | 0.61 | 0.51 | 0.78 |
| VARS | 0.11 | -1.61 | 0.36 |  | HLA-DQB2 | 0.63 | -0.48 | 0.79 |
| ATF6B | 0.12 | -1.54 | 0.37 |  | CYP21A2 | 0.65 | 0.45 | 0.79 |
| HSPA1B | 0.12 | -1.54 | 0.37 |  | SLC44A4 | 0.67 | -0.42 | 0.81 |
| HLA-C | 0.13 | 1.51 | 0.38 |  | HLA-DQA2 | 0.69 | 0.40 | 0.81 |
| MSH5 | 0.15 | -1.45 | 0.39 |  | VWA7 | 0.72 | -0.36 | 0.83 |
| PBX2 | 0.15 | -1.45 | 0.39 |  | NCR3 | 0.79 | 0.26 | 0.90 |
| NOTCH4 | 0.16 | 1.40 | 0.41 |  | EGFL8 | 0.82 | 0.22 | 0.92 |
| HLA-DOB | 0.17 | -1.36 | 0.42 |  | CFB | 0.85 | 0.19 | 0.93 |
| DDX39B | 0.19 | -1.32 | 0.44 |  | BAG6 | 0.97 | -0.04 | 0.98 |
| HLA-DQA1 | 0.19 | -1.30 | 0.44 |  | LTA | 0.93 | -0.09 | 0.98 |
| MICB | 0.20 | -1.28 | 0.44 |  | LY6G6C | 0.95 | -0.06 | 0.98 |
| LY6G6F | 0.21 | -1.25 | 0.45 |  | NFKBIL1 | 0.96 | 0.06 | 0.98 |
| LTB | 0.22 | 1.22 | 0.46 |  | TNF | 0.95 | 0.07 | 0.98 |
| C4B | 0.26 | 1.13 | 0.51 |  | PPT2 | 0.99 | 0.01 | 0.99 |

**^a^There were 60 cases with expected number of 06:02 alleles = 0 and 27 cases with expected number of alleles = 1**

**S12: 214 Novel SNPs Meeting Carry-forward Threshold at P<1x10^-4^ in Imputation Analysis using 1000 Genomes Reference.**

| **Chr** | **SNP** | **Position** | **Minor Allele Frequency^a^** | **.info^b^** | **P Imputed^c^** |
| --- | --- | --- | --- | --- | --- |
| 1 | rs12737202 | 3323025 | 0.118 | 0.833 | 9.84E-05 |
| 1 | rs2483222 | 3323109 | 0.199 | 0.859 | 6.46E-06 |
| 1 | rs2493303 | 3324209 | 0.218 | 0.856 | 3.89E-07 |
| 1 | rs12063572 | 3325260 | 0.206 | 0.848 | 5.21E-06 |
| 1 | rs12065021 | 3325494 | 0.219 | 0.852 | 1.85E-06 |
| 1 | rs35396425 | 3325659 | 0.193 | 0.844 | 7.68E-06 |
| 1 | rs36070715 | 3326094 | 0.217 | 0.85 | 1.06E-06 |
| 1 | rs60965010 | 3326142 | 0.206 | 0.846 | 6.24E-06 |
| 1 | rs59831144 | 3326188 | 0.217 | 0.85 | 1.05E-06 |
| 1 | chr1:3326478:D | 3326478 | 0.199 | 0.837 | 4.69E-06 |
| 1 | rs1106270 | 3327549 | 0.367 | 0.888 | 5.12E-05 |
| 1 | rs72851311 | 3330390 | 0.172 | 0.808 | 2.32E-05 |
| 1 | rs10914570 | 30353286 | 0.354 | 0.96 | 4.89E-05 |
| 1 | rs10458371 | 30353829 | 0.353 | 0.96 | 5.46E-05 |
| 1 | rs11161702 | 86352203 | 0.225 | 0.898 | 5.69E-05 |
| 1 | chr1:116097822:I | 116097822 | 0.107 | 0.923 | 5.51E-05 |
| 1 | rs17486085 | 116100598 | 0.102 | 0.95 | 9.10E-05 |
| 1 | rs12024928 | 116139525 | 0.101 | 0.968 | 7.87E-05 |
| 1 | rs72701620 | 116151729 | 0.054 | 0.859 | 5.42E-05 |
| 1 | rs61799000 | 116154748 | 0.098 | 0.993 | 7.75E-05 |
| 1 | rs4451544 | 116174985 | 0.098 | 0.998 | 9.75E-05 |
| 1 | rs61453587 | 116192382 | 0.098 | 0.989 | 9.77E-05 |
| 1 | rs4440850 | 116197810 | 0.095 | 0.997 | 8.79E-05 |
| 1 | rs4132224 | 116200935 | 0.095 | 0.998 | 6.77E-05 |
| 1 | rs6658884 | 116202811 | 0.093 | 0.983 | 9.91E-05 |
| 1 | rs3738484 | 150552330 | 0.458 | 0.987 | 6.30E-05 |
| 1 | rs3738485 | 150552392 | 0.458 | 0.987 | 7.50E-05 |
| 1 | rs9803935 | 150552622 | 0.458 | 0.987 | 6.44E-05 |
| 1 | rs4970918 | 150553348 | 0.46 | 0.981 | 5.49E-05 |
| 1 | rs12125672 | 150554470 | 0.458 | 0.987 | 6.34E-05 |
| 1 | rs61820165 | 150555987 | 0.458 | 0.987 | 6.66E-05 |
| 1 | rs12139563 | 150578241 | 0.459 | 0.962 | 8.70E-05 |
| 1 | rs77375303 | 205921799 | 0.079 | 0.859 | 4.62E-05 |
| 1 | rs74144114 | 205922403 | 0.076 | 0.861 | 3.54E-05 |
| 1 | chr1:209865963:D | 209865963 | 0.278 | 0.536 | 4.33E-05 |
| 1 | rs116200489 | 210040497 | 0.073 | 0.785 | 3.69E-05 |
| 1 | rs12133086 | 210062043 | 0.087 | 0.87 | 7.05E-05 |
| 1 | rs6695101 | 210098466 | 0.163 | 0.958 | 8.59E-05 |
| 1 | rs227199 | 210265384 | 0.356 | 0.991 | 7.91E-05 |
| 1 | chr1:210267382:I | 210267382 | 0.359 | 0.982 | 5.13E-05 |
| 1 | chr1:210267383:I | 210267383 | 0.359 | 0.982 | 5.27E-05 |
| 1 | rs9659836 | 210279938 | 0.357 | 0.995 | 8.32E-05 |
| 1 | rs4844949 | 210306846 | 0.358 | 0.998 | 5.53E-05 |
| 1 | chr1:214638383:D | 214638383 | 0.175 | 0.837 | 9.36E-05 |
| 1 | rs72737287 | 215636099 | 0.076 | 0.935 | 8.34E-05 |
| 1 | rs28494113 | 236668601 | 0.085 | 0.746 | 8.83E-05 |
| 2 | rs493875 | 13624276 | 0.151 | 0.631 | 6.86E-05 |
| 2 | rs2637993 | 113853444 | 0.253 | 0.869 | 6.15E-05 |
| 2 | rs11893733 | 114359909 | 0.268 | 0.549 | 1.51E-05 |
| 2 | rs11888391 | 114360223 | 0.261 | 0.556 | 5.05E-05 |
| 2 | rs76723748 | 179024166 | 0.07 | 0.972 | 2.85E-05 |
| 2 | chr2:179024358:D | 179024358 | 0.069 | 0.987 | 4.03E-05 |
| 2 | chr2:179024363:D | 179024363 | 0.068 | 0.987 | 4.20E-05 |
| 2 | rs74579095 | 179026871 | 0.068 | 0.991 | 4.67E-05 |
| 3 | rs62271731 | 102837577 | 0.24 | 0.988 | 6.14E-05 |
| 3 | rs62271732 | 102839329 | 0.24 | 0.987 | 6.16E-05 |
| 4 | rs4693442 | 83392129 | 0.439 | 0.97 | 9.92E-05 |
| 4 | rs4693445 | 83394290 | 0.492 | 0.966 | 8.21E-05 |
| 4 | rs10014975 | 83398575 | 0.44 | 0.943 | 8.92E-05 |
| 4 | rs10003479 | 83398777 | 0.446 | 0.94 | 2.63E-05 |
| 4 | chr4:127432348:I | 127432348 | 0.393 | 0.972 | 9.36E-05 |
| 5 | rs115873585 | 12331261 | 0.062 | 0.703 | 9.85E-05 |
| 5 | rs77614178 | 73202854 | 0.05 | 0.587 | 8.77E-05 |
| 5 | chr5:164219338:D | 164219338 | 0.137 | 0.78 | 3.17E-05 |
| 6 | rs78639325 | 5496482 | 0.064 | 0.828 | 4.09E-05 |
| 6 | rs2025983 | 21974070 | 0.098 | 0.619 | 1.66E-05 |
| 6 | rs114671705 | 28719755 | 0.266 | 0.995 | 2.64E-05 |
| 6 | rs147881296 | 28721534 | 0.26 | 0.992 | 1.08E-05 |
| 6 | rs116358736 | 28727593 | 0.266 | 0.979 | 1.91E-05 |
| 6 | rs115401572 | 28728329 | 0.266 | 0.993 | 1.83E-05 |
| 6 | chr6:28866209:D | 28866209 | 0.174 | 0.995 | 6.32E-06 |
| 6 | chr6:29048680:I | 29048680 | 0.223 | 0.883 | 4.57E-05 |
| 6 | rs115221895 | 29328834 | 0.334 | 0.997 | 2.37E-05 |
| 6 | rs114788767 | 29352631 | 0.407 | 0.995 | 8.71E-05 |
| 6 | rs115938288 | 29372323 | 0.096 | 0.996 | 7.82E-05 |
| 6 | rs137966363 | 29375802 | 0.096 | 0.996 | 7.75E-05 |
| 6 | rs115620922 | 29376385 | 0.096 | 0.996 | 7.73E-05 |
| 6 | rs116813346 | 29477414 | 0.102 | 0.991 | 3.71E-05 |
| 6 | rs138291794 | 29480224 | 0.103 | 0.996 | 8.36E-05 |
| 6 | rs115972880 | 29483620 | 0.103 | 0.996 | 8.37E-05 |
| 6 | rs116819367 | 29484110 | 0.101 | 0.996 | 7.99E-05 |
| 6 | rs112340183 | 31228410 | 0.368 | 0.969 | 3.12E-05 |
| 6 | rs116767653 | 31247414 | 0.381 | 0.997 | 2.50E-05 |
| 6 | rs115417199 | 31303176 | 0.242 | 0.915 | 8.36E-05 |
| 6 | rs116378918 | 31307885 | 0.402 | 0.925 | 5.27E-06 |
| 6 | rs116112318 | 31308268 | 0.247 | 0.91 | 7.11E-05 |
| 6 | rs114288467 | 31308963 | 0.264 | 0.882 | 5.65E-05 |
| 6 | rs115810398 | 31308983 | 0.255 | 0.892 | 3.73E-05 |
| 6 | rs115113619 | 31309113 | 0.261 | 0.884 | 5.67E-05 |
| 6 | rs147027370 | 31309423 | 0.267 | 0.903 | 6.58E-05 |
| 6 | rs115511653 | 31309604 | 0.33 | 0.89 | 8.30E-05 |
| 6 | chr6:31309966:I | 31309966 | 0.309 | 0.901 | 5.95E-05 |
| 6 | rs116048584 | 31310056 | 0.257 | 0.884 | 3.23E-05 |
| 6 | rs114962812 | 31310075 | 0.259 | 0.881 | 2.98E-05 |
| 6 | rs114030444 | 31310098 | 0.24 | 0.913 | 3.06E-05 |
| 6 | rs140930322 | 31310378 | 0.251 | 0.897 | 4.89E-05 |
| 6 | rs112882745 | 31311355 | 0.447 | 0.94 | 2.31E-05 |
| 6 | rs112126319 | 31311533 | 0.452 | 0.946 | 3.17E-05 |
| 6 | rs112106462 | 31312058 | 0.459 | 0.956 | 2.94E-05 |
| 6 | rs112940434 | 31313733 | 0.353 | 0.959 | 3.57E-05 |
| 6 | rs115288305 | 31316760 | 0.359 | 0.925 | 2.78E-05 |
| 6 | rs116282186 | 31316762 | 0.371 | 0.958 | 2.57E-05 |
| 6 | rs115567562 | 31316764 | 0.371 | 0.957 | 2.25E-05 |
| 6 | rs116297016 | 31316880 | 0.402 | 0.995 | 1.02E-05 |
| 6 | rs114696672 | 31317115 | 0.402 | 0.994 | 1.14E-05 |
| 6 | rs115104719 | 31317182 | 0.402 | 0.995 | 1.03E-05 |
| 6 | rs111816871 | 31317446 | 0.402 | 0.995 | 1.03E-05 |
| 6 | chr6:31317750:D | 31317750 | 0.394 | 0.977 | 1.20E-05 |
| 6 | rs116153152 | 31317820 | 0.402 | 0.994 | 1.17E-05 |
| 6 | rs114661985 | 31317976 | 0.402 | 0.993 | 1.17E-05 |
| 6 | rs114551728 | 31318164 | 0.402 | 0.993 | 1.19E-05 |
| 6 | chr6:31318394:D | 31318394 | 0.402 | 0.989 | 1.15E-05 |
| 6 | rs115816431 | 31318978 | 0.401 | 0.973 | 6.49E-06 |
| 6 | rs116169317 | 31318985 | 0.401 | 0.971 | 7.06E-06 |
| 6 | rs112026421 | 31319620 | 0.5 | 0.941 | 8.52E-05 |
| 6 | rs9266075 | 31319754 | 0.351 | 0.905 | 1.38E-06 |
| 6 | rs35278939 | 31319780 | 0.164 | 0.935 | 5.13E-05 |
| 6 | chr6:31320005:D | 31320005 | 0.318 | 0.942 | 1.76E-05 |
| 6 | rs943079 | 43826224 | 0.268 | 0.99 | 6.44E-05 |
| 6 | rs148792542 | 68918782 | 0.062 | 0.594 | 3.47E-05 |
| 6 | rs5024230 | 117268483 | 0.251 | 0.86 | 9.10E-05 |
| 6 | rs6570767 | 146871621 | 0.207 | 0.959 | 8.18E-05 |
| 7 | chr7:55735418:D | 55735418 | 0.213 | 0.876 | 8.93E-05 |
| 7 | rs10953351 | 101437863 | 0.339 | 0.913 | 7.90E-05 |
| 7 | rs28526688 | 117650345 | 0.2 | 0.984 | 7.22E-05 |
| 7 | rs11771150 | 117650783 | 0.2 | 0.984 | 7.18E-05 |
| 7 | rs10249165 | 117652319 | 0.2 | 0.984 | 7.49E-05 |
| 7 | rs10278862 | 117652427 | 0.203 | 0.979 | 6.55E-05 |
| 7 | rs10235515 | 117652447 | 0.2 | 0.984 | 7.31E-05 |
| 7 | rs28455850 | 117652848 | 0.2 | 0.984 | 7.10E-05 |
| 7 | rs722900 | 117653094 | 0.2 | 0.984 | 7.17E-05 |
| 7 | rs13226962 | 117656762 | 0.2 | 0.984 | 7.14E-05 |
| 7 | rs13227352 | 117657060 | 0.2 | 0.984 | 7.30E-05 |
| 7 | rs28882150 | 117657855 | 0.201 | 0.982 | 8.22E-05 |
| 7 | rs1548458 | 117662656 | 0.2 | 0.982 | 8.38E-05 |
| 7 | rs10241640 | 117664640 | 0.2 | 0.982 | 9.17E-05 |
| 7 | chr7:117665002:I | 117665002 | 0.2 | 0.982 | 8.61E-05 |
| 7 | rs10215788 | 117665525 | 0.2 | 0.982 | 8.80E-05 |
| 7 | rs763890 | 117666732 | 0.2 | 0.982 | 8.82E-05 |
| 7 | rs10243335 | 117669007 | 0.194 | 0.962 | 7.80E-05 |
| 7 | rs10258900 | 117669204 | 0.2 | 0.982 | 9.33E-05 |
| 7 | rs10246625 | 117669416 | 0.2 | 0.982 | 9.33E-05 |
| 7 | rs4419753 | 117669601 | 0.2 | 0.982 | 9.33E-05 |
| 7 | rs7456706 | 117669962 | 0.2 | 0.982 | 9.33E-05 |
| 7 | rs28412919 | 117673134 | 0.199 | 0.973 | 7.73E-05 |
| 7 | chr7:124898685:D | 124898685 | 0.275 | 0.815 | 9.95E-05 |
| 8 | rs4360320 | 6127609 | 0.243 | 0.919 | 6.03E-05 |
| 8 | rs12674939 | 126479907 | 0.226 | 0.961 | 7.89E-05 |
| 8 | rs185024604 | 136446894 | 0.134 | 0.601 | 9.43E-05 |
| 9 | chr9:14344410:I | 14344410 | 0.068 | 0.68 | 9.35E-05 |
| 9 | rs10971408 | 33105124 | 0.139 | 0.977 | 3.31E-05 |
| 9 | rs28641537 | 82770292 | 0.299 | 0.996 | 6.98E-05 |
| 9 | rs620889 | 90730148 | 0.465 | 0.686 | 9.91E-05 |
| 9 | rs1514776 | 120668782 | 0.415 | 0.991 | 1.37E-05 |
| 9 | rs1849815 | 120671273 | 0.421 | 0.989 | 1.06E-05 |
| 10 | rs4584477 | 4345383 | 0.386 | 0.99 | 9.82E-05 |
| 10 | rs10764438 | 24263477 | 0.304 | 0.959 | 5.68E-05 |
| 11 | rs10831658 | 2279372 | 0.227 | 0.9 | 7.63E-05 |
| 11 | rs4756890 | 17509400 | 0.287 | 0.994 | 1.43E-05 |
| 11 | rs4756891 | 17509530 | 0.282 | 0.983 | 4.29E-05 |
| 11 | rs4756893 | 17509733 | 0.286 | 0.995 | 1.34E-05 |
| 11 | rs7926717 | 118121399 | 0.193 | 0.983 | 7.04E-05 |
| 11 | rs11216839 | 118129583 | 0.198 | 0.995 | 6.92E-05 |
| 11 | chr11:118130540:I | 118130540 | 0.196 | 0.992 | 5.35E-05 |
| 11 | rs2120268 | 118132705 | 0.198 | 0.99 | 6.91E-05 |
| 11 | rs78389301 | 126872005 | 0.108 | 0.951 | 1.31E-05 |
| 11 | rs7115473 | 126873226 | 0.119 | 0.967 | 3.35E-05 |
| 11 | rs12279007 | 126877035 | 0.114 | 0.987 | 3.89E-05 |
| 11 | rs73577014 | 126881360 | 0.114 | 0.987 | 4.48E-05 |
| 11 | rs4245135 | 134348134 | 0.44 | 0.538 | 5.82E-05 |
| 12 | rs28434700 | 4822465 | 0.391 | 0.815 | 7.01E-05 |
| 12 | rs12817813 | 121743682 | 0.271 | 0.955 | 6.46E-05 |
| 12 | rs146018829 | 121758491 | 0.275 | 0.972 | 7.29E-05 |
| 12 | chr12:123842166:D | 123842166 | 0.495 | 0.625 | 7.57E-05 |
| 13 | rs9552770 | 23504567 | 0.158 | 0.585 | 2.17E-05 |
| 13 | rs9552771 | 23504568 | 0.158 | 0.585 | 2.17E-05 |
| 13 | rs4770341 | 23507313 | 0.227 | 0.592 | 9.46E-06 |
| 13 | rs4770342 | 23507317 | 0.229 | 0.593 | 9.70E-06 |
| 13 | rs4770343 | 23507369 | 0.234 | 0.581 | 1.21E-05 |
| 13 | rs9554177 | 28430981 | 0.393 | 0.994 | 9.58E-05 |
| 13 | rs7994327 | 34357182 | 0.298 | 0.971 | 3.98E-05 |
| 13 | rs7491126 | 110201558 | 0.062 | 0.921 | 8.94E-05 |
| 14 | rs4901737 | 57247331 | 0.1 | 0.803 | 5.78E-05 |
| 14 | chr14:103034541:I | 103034541 | 0.231 | 0.951 | 8.52E-05 |
| 14 | rs4325492 | 103039430 | 0.213 | 0.987 | 9.64E-05 |
| 14 | rs4900545 | 103051132 | 0.237 | 0.955 | 8.47E-05 |
| 15 | chr15:45023268:I | 45023268 | 0.139 | 0.606 | 6.25E-05 |
| 15 | rs6493123 | 45042907 | 0.095 | 0.645 | 8.03E-06 |
| 15 | chr15:55519525:D | 55519525 | 0.235 | 0.923 | 4.17E-05 |
| 16 | chr16:7932925:I | 7932925 | 0.123 | 0.978 | 4.47E-05 |
| 16 | rs7200075 | 7933478 | 0.121 | 0.995 | 5.67E-05 |
| 16 | rs12446367 | 7934218 | 0.121 | 0.993 | 6.01E-05 |
| 16 | rs35932145 | 7948364 | 0.122 | 0.944 | 8.56E-05 |
| 16 | rs9673178 | 28258151 | 0.298 | 0.989 | 9.26E-05 |
| 16 | rs9673810 | 28258384 | 0.298 | 0.989 | 9.11E-05 |
| 16 | rs9673197 | 28258418 | 0.298 | 0.989 | 9.10E-05 |
| 16 | rs4453501 | 28260001 | 0.296 | 0.985 | 9.36E-05 |
| 16 | rs11865651 | 28260638 | 0.298 | 0.99 | 8.44E-05 |
| 16 | rs4412973 | 28261270 | 0.298 | 0.992 | 8.93E-05 |
| 16 | rs4624180 | 28261511 | 0.298 | 0.992 | 8.89E-05 |
| 16 | rs4293368 | 82963443 | 0.308 | 0.955 | 2.78E-05 |
| 16 | chr16:82964078:I | 82964078 | 0.349 | 0.962 | 4.86E-05 |
| 16 | rs6565083 | 82964541 | 0.339 | 0.995 | 3.98E-05 |
| 16 | rs74034596 | 86391472 | 0.084 | 0.952 | 9.05E-05 |
| 18 | rs66790221 | 656904 | 0.342 | 0.766 | 5.91E-05 |
| 18 | rs8092511 | 3384486 | 0.447 | 0.748 | 8.17E-05 |
| 19 | rs12462166 | 41857404 | 0.325 | 0.989 | 8.08E-05 |
| 19 | rs8105450 | 41886859 | 0.444 | 0.935 | 2.78E-05 |
| 20 | rs3790304 | 17926263 | 0.222 | 0.866 | 3.50E-05 |
| 20 | chr20:33672445:D | 33672445 | 0.167 | 0.905 | 3.29E-05 |
| 20 | chr20:35698883:D | 35698883 | 0.096 | 0.817 | 2.97E-05 |
| 20 | rs35902944 | 62308395 | 0.24 | 0.756 | 3.18E-06 |
| 21 | rs2223173 | 29293258 | 0.446 | 0.638 | 1.68E-05 |
| 23 | rs4898353 | 154169075 | 0.313 | 0.866 | 4.23E-05 |

**^a^Minor allele frequency**

**^b^Quality score (.info) for imputation**

**^c^Genomic-control corrected p-value from imputation analysis under additive model using discovery GWAS samples**

**Figures:**

**S1: Q-Q plots of P-values from SNPTest using Imputed Dosage for a) Genotyped SNPs and b) Imputed SNPs Prior to Genomic Control Adjustment Based on Genotyped SNP P-values from GEMMA analysis (see manuscript statistical methods for details).**

**
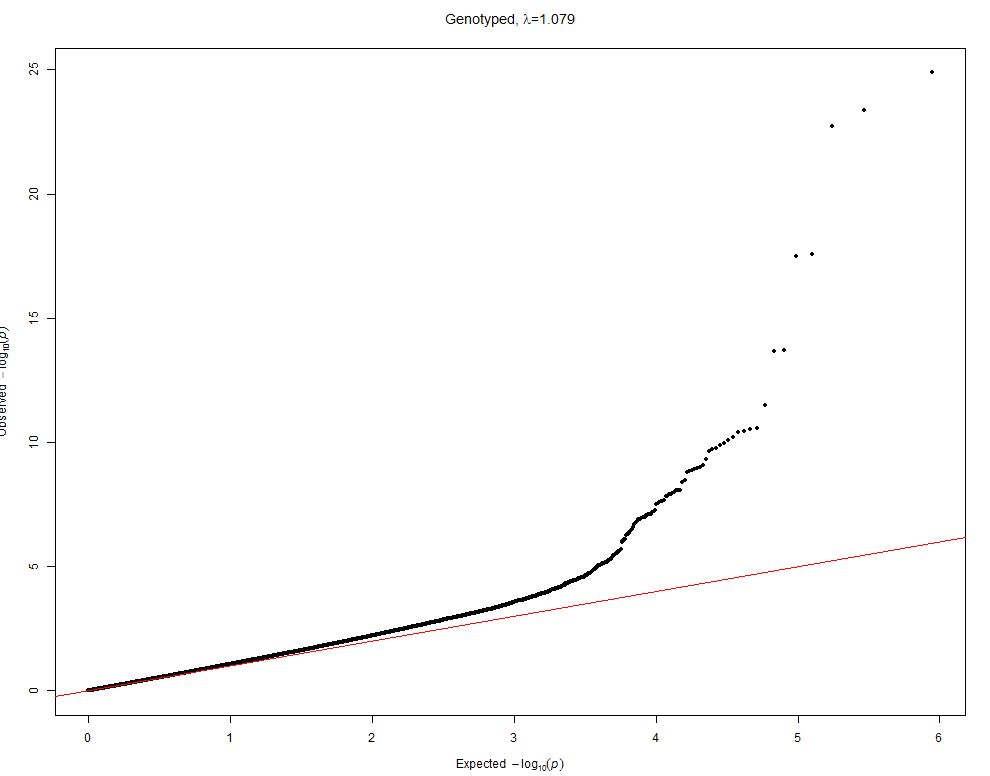

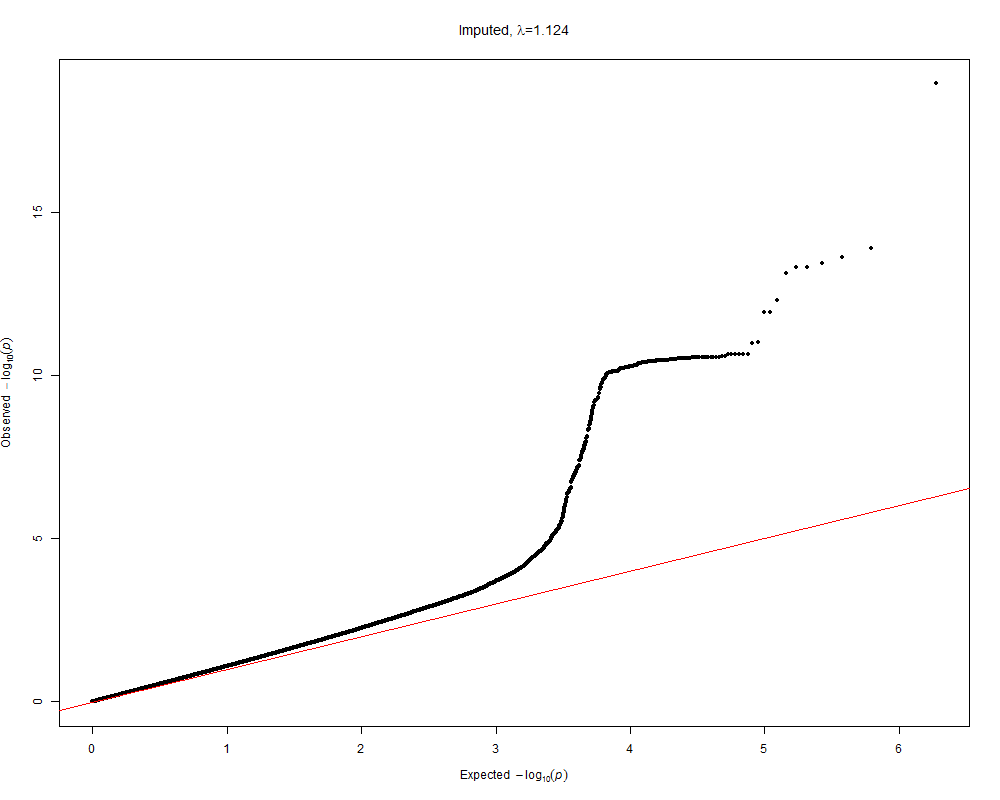
**

**S2: Q-Q plot of imputed (adjusted) p-values (inflation factor = 1.04) after genomic control correction**

**
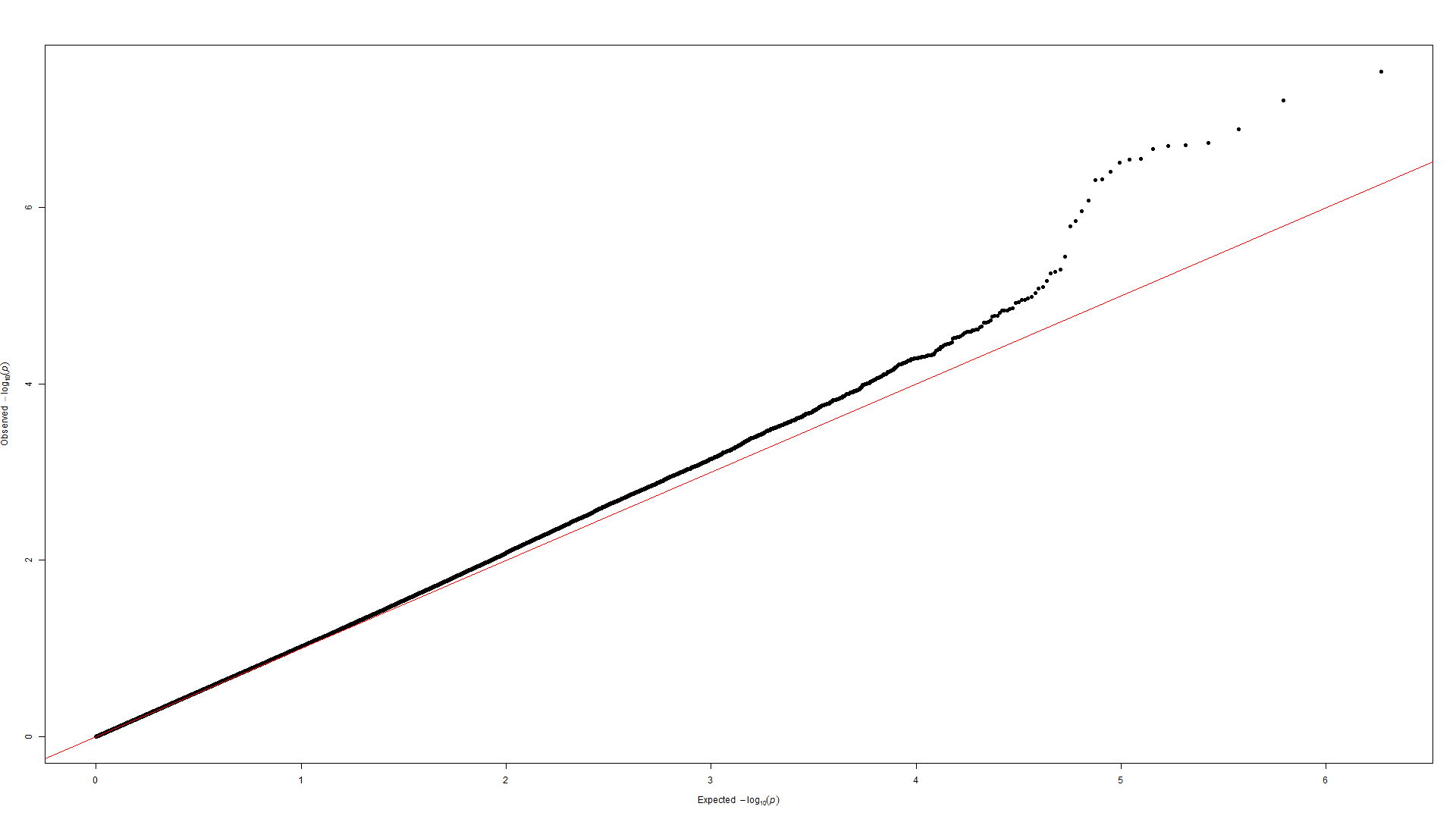
**

**S3: Imputation GWAS Results Figure 1:** Imputation GWAS results with 1616 cases and 4683 controls under additive model. SNPs above red line were genome-wide significant at *P* < 5x10^-8.^ A subset of these SNPs and SNPs between red and blue lines, corresponding to 5x10^-8^ < *P*-value <.0001, were selected for follow-up and genotyped in 878 cases and 2017 controls.

**
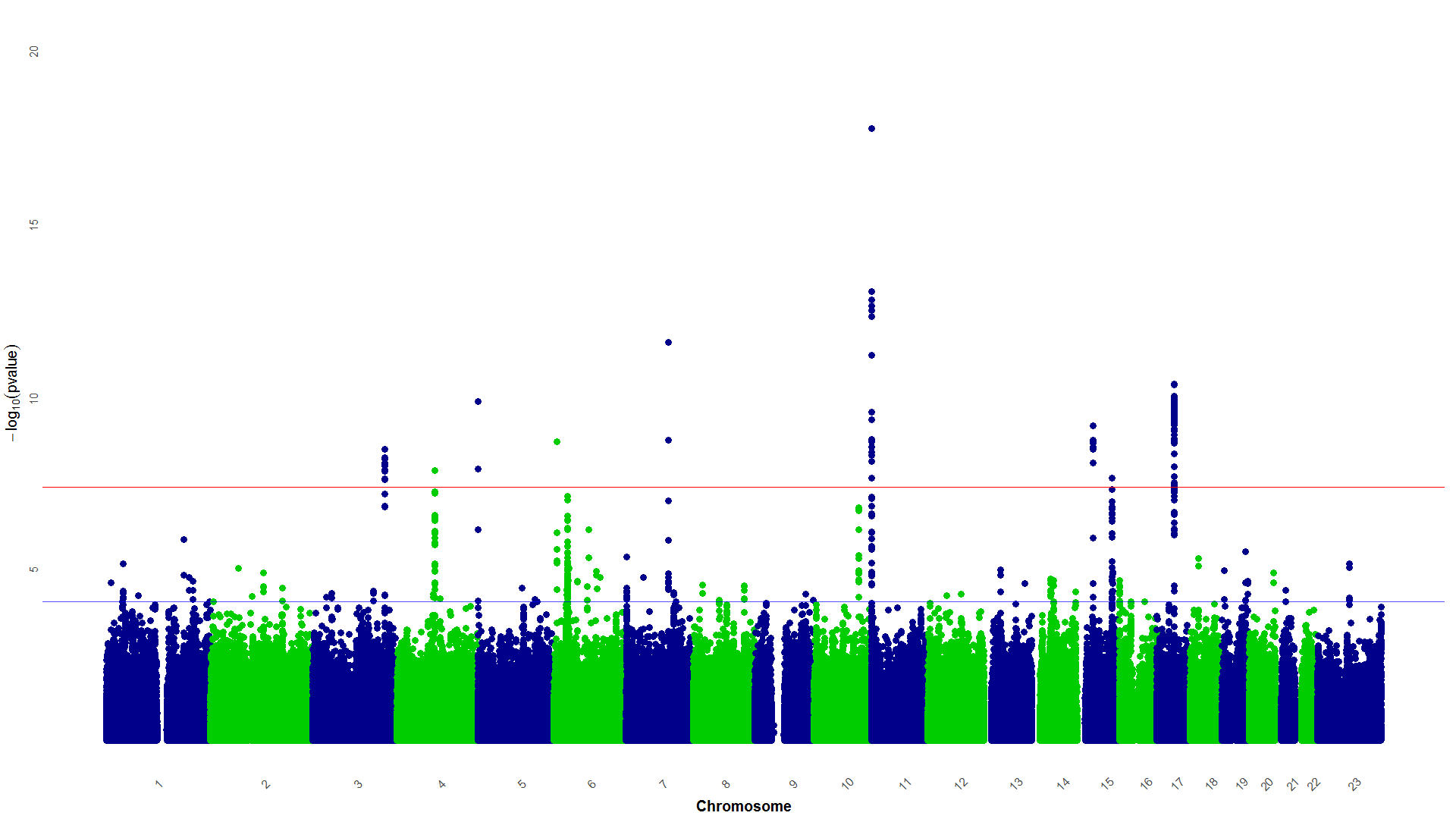
**

**S4: Histogram of .INFO Scores from SNPTest. Only SNPs with .INFO >0.5 Included.**


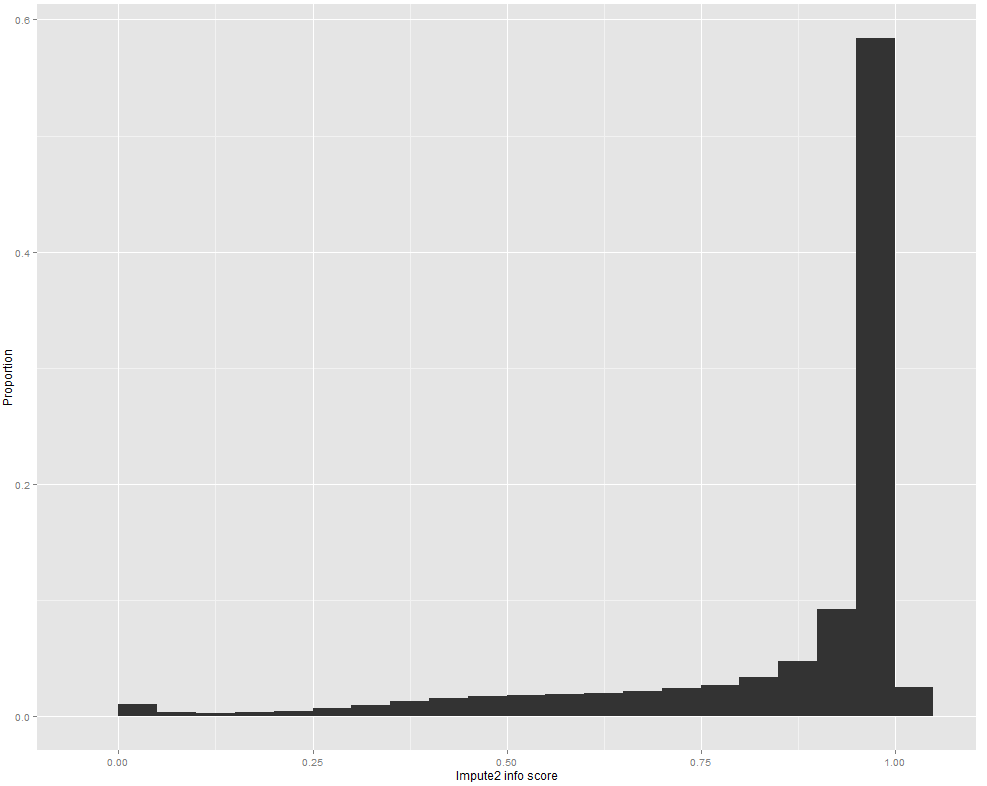


**S5: Comparison of SNPTest p-values after genomic control (see manuscript statistical methods) and GEMMA dosage-values. GEMMA dosage p-values computed after initial study complete.**

**
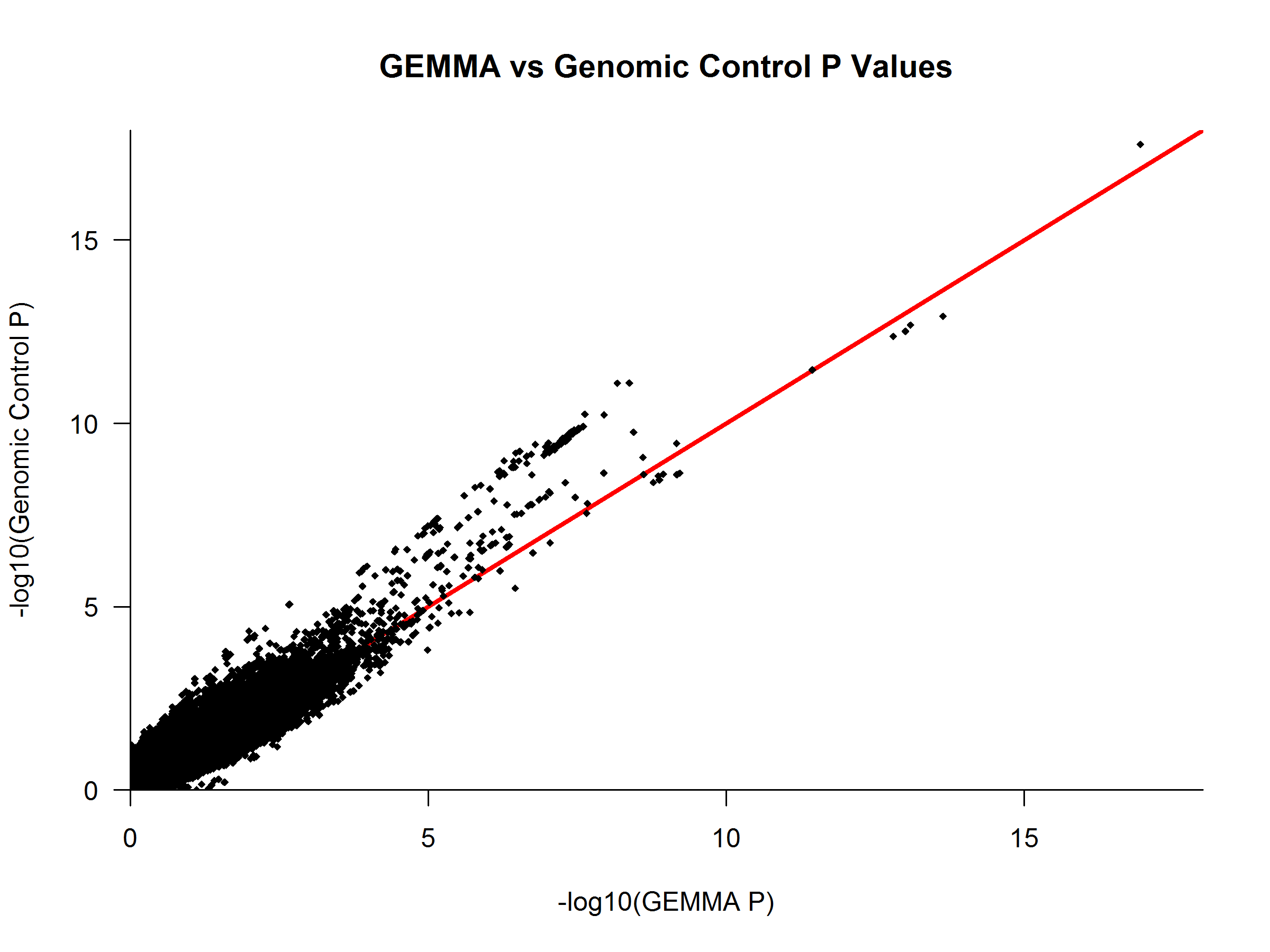
**
